# How well does molecular simulation reproduce environment-specific conformations of the intrinsically disordered peptides PLP, TP2 and ONEG?†

**DOI:** 10.1039/d1sc03496k

**Published:** 2022-01-20

**Authors:** Lauren M. Reid, Ileana Guzzetti, Tor Svensson, Anna-Carin Carlsson, Wu Su, Tomas Leek, Lena von Sydow, Werngard Czechtizky, Marija Miljak, Chandra Verma, Leonardo De Maria, Jonathan W. Essex

**Affiliations:** School of Chemistry, University of Southampton Highfield Southampton SO17 1BJ UK jwe1@soton.ac.uk; Bioinformatics Institute (A*STAR) 30 Biolpolis Street Matrix 138671 Singapore; MedChemica Ltd Alderley Park Macclesfield Cheshire SK10 4TG UK; Medical Chemistry, Research and Early Development, Respiratory & Immunology, BioPharmaceuticals R&D AstraZeneca Gothenburg Sweden; Early Chemical Development, Pharmaceutical Sciences, BioPharmaceuticals R&D AstraZeneca Gothenburg Sweden; Department of Biological Sciences, National University of Singapore 16 Science Drive 4 117558 Singapore; School of Biological Sciences, Nanyang Technological University 60 Nanyang Dr 637551 Singapore

## Abstract

Understanding the conformational ensembles of intrinsically disordered proteins and peptides (IDPs) in their various biological environments is essential for understanding their mechanisms and functional roles in the proteome, leading to a greater knowledge of, and potential treatments for, a broad range of diseases. To determine whether molecular simulation is able to generate accurate conformational ensembles of IDPs, we explore the structural landscape of the PLP peptide (an intrinsically disordered region of the proteolipid membrane protein) in aqueous and membrane-mimicking solvents, using replica exchange with solute scaling (REST2), and examine the ability of four force fields (ff14SB, ff14IDPSFF, CHARMM36 and CHARMM36m) to reproduce literature circular dichroism (CD) data. Results from variable temperature (VT) ^1^H and Rotating frame Overhauser Effect SpectroscopY (ROESY) nuclear magnetic resonance (NMR) experiments are also presented and are consistent with the structural observations obtained from the simulations and CD. We also apply the optimum simulation protocol to TP2 and ONEG (a cell-penetrating peptide (CPP) and a negative control peptide, respectively) to gain insight into the structural differences that may account for the observed difference in their membrane-penetrating abilities. Of the tested force fields, we find that CHARMM36 and CHARMM36m are best suited to the study of IDPs, and accurately predict a disordered to helical conformational transition of the PLP peptide accompanying the change from aqueous to membrane-mimicking solvents. We also identify an α-helical structure of TP2 in the membrane-mimicking solvents and provide a discussion of the mechanistic implications of this observation with reference to the previous literature on the peptide. From these results, we recommend the use of CHARMM36m with the REST2 protocol for the study of environment-specific IDP conformations. We believe that the simulation protocol will allow the study of a broad range of IDPs that undergo conformational transitions in different biological environments.

## Abstract 2

A protocol for simulating intrinsically disordered peptides in aqueous and hydrophobic solvents is proposed. Results from four force fields are compared with experiment. CHARMM36m performs the best for the simulated IDPs in all environments.

## Graphical abstract

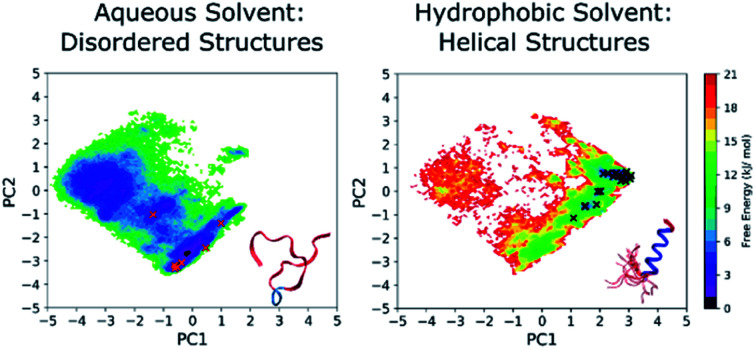

## Introduction

Intrinsically disordered proteins and peptides (IDPs) do not adopt stable secondary or tertiary structures, and are best structurally characterised as ensembles of flexible conformations.^1^ While the traditional paradigm holds that biological processes are mediated by macromolecules with fixed three-dimensional structures, it is now becoming increasingly evident that IDPs play a very important role.^2^ In the absence of a stable structure and characterised by high levels of flexibility, IDPs can easily interconvert between multiple conformations. This enables them to bind to different macromolecules, resulting in diverse functionality.^2–6^ For example, the intrinsically disordered N-terminal transactivation domain of the protein p53 is structured into a helix upon binding to a hydrophobic cleft in the proteins MDM2 or p300.^3,7–9^ Similarly the disordered C-terminal region of p53 has been shown to adopt diverse secondary structures when complexed to different protein partners.^10^ The study of the structural dynamics of IDPs and their participation in protein–protein interactions (PPIs) using computational and experimental studies is increasingly illuminating the subtle yet complex nature of molecular interactions governing biomolecular mechanisms.^10^

The ability of IDPs to assume different shapes depending on the interacting partner or the environment may also enable them to play a significant role in the multimerisation of membrane proteins. For example, the 19-residue C-terminus of the membrane proteolipid protein (referred to as the PLP peptide henceforth) is intrinsically disordered and resides on the cytoplasmic side of the membrane.^6^ Structural studies of the PLP peptide in different biologically-relevant solvent environments have suggested that it is involved in the multimerisation of the full protein through its flexible rearrangement into a membrane-associated helix, followed by its aggregation and β-sheet formation with the C-terminal peptides from other PLP monomers.^6^ The aggregation of IDPs is also known to be associated with several diseases,^11^ including Alzheimer's disease in which intrinsically disordered amyloid β (Aβ) peptides form misfolded oligomers that lead to the formation of fibrils.^12^ Studies have shown that the conformational ensembles of Aβ monomers are very sensitive to osmolyte concentrations, and it is thought that such environment-dependent conformational transitions promote oligomerisation.^13^

There are many known cell-penetrating and antimicrobial peptides (CPPs and AMPs, respectively) that are intrinsically disordered in aqueous solution but adopt ordered structures upon binding to, or insertion into, cell membranes.^6,14,15^ CPPs are a class of small peptides that are found to translocate across cell membranes and can deliver large, polar molecules into cells,^16^ while AMPs are found to interact with and destabilise bacterial cell membranes, causing cell death.^17^ The conformational sensitivity of such peptides to their biological environment is essential for the membrane-penetration process, since the arrangement of hydrophobic and hydrophilic residue side chains and the shielding of the polar backbone through the formation of intramolecular hydrogen bonds, allow the peptides to interact favourably with the polar headgroups and the lipid chain regions of cell membranes. There is much interest in deducing the exact mechanisms by which CPPs and AMPs interact with cell membranes, as this knowledge could prove crucial in aiding the design of intracellular delivery systems, biomedical imaging agents and/or new antimicrobial treatments in the fight against antibiotic resistance.^16–19^

From the examples given above, it is obvious that IDPs are involved in a broad range of biological processes and offer an exciting line of study. However, the very nature of IDPs (their flexibility and lack of defined structure) makes them a difficult target for conventional experimental techniques, such as X-ray crystallography that is used to capture the crystalline structure of proteins, and instead techniques that enable the measurement of dynamic structures are necessary. Examples of such techniques that have been used to study IDPs include nuclear magnetic resonance (NMR),^20^ circular dichroism (CD),^20^ Raman spectroscopy,^21^ Fourier transform infrared spectroscopy (FT-IR),^22^ small-angle X-ray scattering (SAXS)^23^ and static and dynamic light scattering (SLS/DLS).^24^ NMR is particularly popular in the study of IDPs as it can provide atomistic detail and detect the presence or absence of structure in solution-phase peptides; however, interpretation of NMR output can be challenging and time-consuming, especially since the diverse conformational ensembles of IDPs lead to noisy signals. CD is also frequently used for IDP characterisation and provides spectra that are characteristic of secondary structure or disorder within the peptide; however, only global structural descriptors can be identified, making CD a low-resolution technique. For a recent review of the structural characterisation techniques used to study IDPs, see ref. 25.

In light of these challenges, atomistic molecular dynamics (MD) simulation, which involves modelling biological systems at the atomistic level and calculating the dynamic evolution of the atoms, provides an alternative or complementary approach to help elucidate the dynamic structures and mechanisms of IDPs.^26,27^ However, biomolecular MD comes with its own set of challenges suggesting that simulation results must be interpreted with caution and validated against experimental data.^28^ One of the biggest challenges is the computational expense involved in modelling biological systems atomistically, with the implication that simulations of large systems can only access μs timescales with currently available hardware. In reality, many biological processes occur over much longer timescales as they can involve multiple complex macromolecular folding, aggregation, binding and insertion events, which are often associated with large free energy barriers.^28^ The simulation of IDPs presents a further challenge since the conformational space available to the peptides is vast and long timescales may be needed to exhaustively sample the full structural landscape. Recent studies have improved IDP conformational sampling by applying enhanced sampling techniques, such as parallel tempering^29^ or replica exchange,^30^ or by incorporating experimental data to restrain the simulation or reweight the resulting structural ensembles.^31^

Another important consideration for biomolecular MD is the choice of force field. Force field accuracy greatly depends on the data and protocol used in the parameterisation procedure and it is often reported that different force fields are better suited to different systems and properties of interest.^32,33^ It is currently accepted that many standard protein force fields are better suited to globular proteins than IDPs, due to the greater availability of globular experimental data for parameterisation.^34–36^ With increasing interest in IDPs, the correction of force fields to better sample disordered structures is a hot topic.^34–40^ Established by Best *et al.*,^39^ one technique has been to refine the protein–water Lennard-Jones parameters to improve solvation and reduce the sampling of compact structures, which was originally applied to the Amber force field ff03w. The resulting force field, ff03ws, was shown to sample more extended and flexible conformations.^35,39^ Similar approaches, where the protein–water interactions were scaled, were adopted by Robustelli *et al.*^36^ and Huang *et al.*^38^ to improve IDP sampling of the Amber ff99SB-ILDN and CHARMM36m force fields, respectively. Approaches that tackle the protein interaction parameters directly have also been utilised to improve IDP sampling, for example some recent force fields include a CMAP correction term (a matrix of energy corrections applied to the ϕ/ψ backbone dihedral space) to encourage the simulation to sample disordered structures.^34,37,38^ This approach has been applied in multiple iterations of the CHARMM force fields,^41,42^ resulting in the most recent CHARMM36m,^38^ and to correct the Amber ff14SB force field^43^ for IDP sampling to create ff14IDPSFF.^44^ However, as has been discussed, many IDPs are involved in environment-dependent conformational transitions, so it is also important to test force fields in a variety of biologically-relevant environments to determine their conformational sensitivity.

In this work, we test the ability of the enhanced conformational sampling approach replica exchange with solute scaling (REST2),^45^ and a number of force fields, to explore the conformations accessed by IDPs in aqueous and hydrophobic solvents, as would be relevant in the study of membrane proteins, CPPs, AMPs, and even for peptides that bind to hydrophobic pockets in other proteins (*e.g.* the p53–MDM2 interaction). With validation in mind, we apply the simulation protocol to the PLP peptide, which has literature circular dichroism (CD) data in aqueous and a variety of membrane-mimicking/hydrophobic environments.^6^ To provide additional experimental indications of the different PLP peptide conformations, variable temperature (VT) ^1^H and Rotating frame Overhauser Effect SpectroscopY (ROESY) nuclear magnetic resonance (NMR) experiments were recorded in aqueous and membrane-mimicking solvent mixtures. Following this, we apply the validated simulation protocol to TP2, a spontaneous membrane translocating peptide (SMTP) that is known to penetrate cells and artificial lipid vesicles as a monomer at low concentrations, and compare the results against a negative control peptide, ONEG, which has a similar amino acid sequence but does not possess membrane-translocating properties.^46^ We show that the CHARMM36 ^42^ and CHARMM36m^38^ force fields are best able to reproduce the conformational transition that occurs in the PLP peptide as the solvent changes from aqueous to membrane-mimicking. ff14SB^43^ overpredicts the helical structure of the PLP peptide and TP2 in aqueous solvent, and ff14IDPSFF^44^ is unsuccessful in predicting the PLP peptide structure in any of the tested solvents. In contrast to a previously reported experimental CD study,^47^ we identify an α-helical structure of TP2 in the membrane-mimicking solvents and discuss the possible mechanistic significance of this observation; such a contradiction, and the additional mechanistic detail that is inferred from simulations, highlights the importance of complementing experimental structural studies with simulation results.

## Methods

### Computational methods

#### System setups

Initial coordinate files for the PLP peptide, TP2 and ONEG were built in extended conformations using the Amber tleap program.^48^ The sequences of the peptides are as follows:^6,47^

PLP: Ac-IAATYNFAVLKLMGRGTKF

TP2: PLIYLRLLRGQF-NH_2_

ONEG: PLGRPQLRRGQF-NH_2_

Where Ac – represents an acetyl N-terminal cap and –NH_2_ represents an amide C-terminal cap.

A single monomer of each peptide was solvated with three different solvent systems to model the different biological environments the peptides encounter during membrane association and penetration:

• Water, neutralising Cl^−^ ions and 0.15 M Na^+^Cl^−^ ions were used to model the extracellular aqueous medium.

• Trifluoroethanol (TFE) : water, ∼1 : 1 mol% (∼8 : 2 vol%) + neutralising Cl^−^ ions were used as a model of the hydrophobic interior of a cell membrane.

• Chloroform : methanol : water, ∼0.25 : 0.48 : 0.27 mol% (∼4 : 4 : 1 vol%) + neutralising Cl^−^ ions were used as a second model of the hydrophobic interior of a cell membrane for the Amber force fields.

• Chloroform : methanol : water, ∼0.24 : 0.64 : 0.11 mol% (∼4.1 : 5.5 : 0.4 vol%) + neutralising Cl^−^ ions were used with the CHARMM force fields.

The choices of solvent models and ratios are justified in the discussion.

Four different force fields were used to model the PLP peptide in each solvent system:

• ff14SB^43^

• ff14IDPSFF^44^

• CHARMM36 ^42^

• CHARMM36m^38^

Two force fields were subsequently used to model TP2 and ONEG in each solvent system:

• ff14SB

• CHARMM36m

For the Amber force fields (ff14SB and ff14IDPSFF), GAFF parameters of TFE were taken from ref. 49 and GAFF parameters for chloroform and methanol were taken from ref. 50. TIP3P water was used with the Amber force fields in all cases, except in the TFE : water mixture where the TIP4P-ew model was used, since this was one of the water models used in the optimisation and validation of the TFE model.^49^ The standard Amber ion parameters were used with the Amber force fields.^43^ For the CHARMM force fields, CGenFF parameters of TFE and methanol were taken from ref. 51 and the Dietz–Heinzinger (DH) model of chloroform^52^ was used (as tested for CHARMM force fields in reference^53^). The CHARMM modified TIP3P water model and the standard CHARMM ion parameters were used with the CHARMM force fields in all cases.^42^

To assess simulation convergence, two simulations were performed for each peptide/force field/solvent combination; (i) one starting from the extended conformation built in tleap, and (ii) one starting from a helical structure found for each peptide in this study. Details of each system setup and simulation length can be found in Table S1.†

#### Simulation protocol

GROMACS-5.1.4 ^54^ patched with Plumed2-2.2 ^55^ was used for all simulations. The leap-frog integrator was used with a time step of 2 fs and periodic boundary conditions were employed. Coulomb interactions were treated with the Particle Mesh Ewald (PME) method, with a real space cutoff of 1.0 nm for the Amber force fields and 1.2 nm for the CHARMM force fields, as is common practice for these force fields. van der Waals interactions were treated with cutoffs of 1.0 nm and 1.2 nm for the Amber and CHARMM force fields respectively. The systems were first energy minimised using the steepest-descent algorithm until the maximum force was <1000 kJ mol^−1^ nm^−1^ and subsequently using the conjugate gradient method until the maximum force was <50 kJ mol^−1^ nm^−1^. The LINCS algorithm was used to constrain all hydrogen-containing bonds except when the DH chloroform was used, in which case the SHAKE algorithm was employed. NVT equilibration was performed on each replica with the Nose–Hoover thermostat^56,57^ at 300 K for 1 ns. This was followed by NPT equilibration with the Parrinello–Rahman barostat^58^ at 1 bar for 1 ns. Each system was simulated using the REST2 protocol described in the ESI,† with the replicas spaced according to Tables S2 and S3† for the PLP peptide and TP2/ONEG respectively. Replica coordinate exchanges were attempted every 1 ps. Each simulation was run until structural convergence was observed (convergence criteria discussed below) or until 1 μs was reached, resulting in the simulation lengths given in Table S1.† The resulting total simulation time reported here is over 225 μs.

#### Analysis methods

The following analyses were performed on the base replica of each simulation:

• Dictionary of protein secondary structure (DSSP)^59^ was implemented using mdtraj,^60^ which assigns to each residue of each frame a secondary structure identifier based on the atomic coordinates of the backbone. These were plotted against simulation time to monitor convergence and were used to calculate the structural content from the equilibrated parts of each trajectory. The average structural content percentages and standard errors were calculated from the two independent simulations of each system.

• Principal component analysis (PCA)^61^ was performed on the backbone ϕ and ψ dihedral angles (using a (sin(*x*), cos(*x*)) transformation on the angles to eradicate wrap around prior to PCA) taken from the full set of base replica data for the given peptide using PyEMMA.^62^ PCA is a data dimensionality reduction technique that assembles a series of linear, non-correlated coordinates that is used to extract the most extreme motions in the data. By combining the base replica data from the different simulation conditions, a generic set of PCs were built for each peptide, such that the results obtained under different simulation conditions for the given peptide could be compared using the same PCs; for example, the 24 base replicas for the PLP peptide (Table S1†) were combined to produce PCs that could be used to compare the conformational space visited by the PLP peptide in the different solvents. The base replica trajectories were divided in half and projected onto the first two principal components (PC1 and PC2) in order to assess convergence.

• Two-dimensional free energy surfaces (FESs) were built from the equilibrated parts of the trajectories with respect to PC1 and PC2, using PyEMMA that implements the following relation:*F*(*x*) = −*k*_B_*T* ln *P*(*x*)where *P*(*x*) is the probability distribution of coordinate *x*, taken as the 2 dimensional histogram of PC1 and PC2.

• MDASH-3 was used to employ the DASH clustering algorithm on the combined equilibrated parts of the base replica trajectory of each peptide/force field/solvent combination.^63^ The DASH algorithm clusters molecular conformations based on the ϕ and ψ backbone dihedral angles explored throughout the simulation. First, the dihedral angle histograms are binned by placing bin edges midway between maxima, which are identified when they are >2.4% of the trajectory frames. Each trajectory frame can then be described by a sequence of dihedral angle states, creating discrete conformational states. Conformational states present in >1% of the trajectory frames are retained as clusters. In this work, DASH clusters have been further clustered into macrostates based on their location on the PC FES.

Simulation convergence was assessed and equilibration times chosen using several criteria:

• When the DSSP plots from the two simulation repeats showed similar secondary structure content.

• When the first and second halves of the equilibrated portions of the two simulations explored a similar PC1 and PC2 space, assessed using histograms.

• When the percentage structural assignment of the two simulations provided an average with a minimal standard error.

### Experimental methods

#### PLP peptide preparation

2-Chlorotrityl chloride resin (300 mg, 0.3 mmol, 1.0–1.8 mmol g^−1^) was swelled in DCM (5 mL) for 1 h. A solution of Fmoc-Phe-OH (81 mg, 0.21 mmol) and DIPEA (209 μL, 1.20 mmol) in DCM (5 mL) was added. The reaction was shaken for 21 h at room temperature. The resin was filtered off and washed with DCM (1×), DMF (1×) and DCM (1×). Subsequently, the resin was added DCM/MeOH/DIPEA (17 : 2 : 1, 10 mL) and the reaction was shaken for 30 min at room temperature. The resin was filtered off and washed with DCM (2×), DMF (1×), DCM (1×), MeOH (1×), DCM (1×), MeOH (1×), DCM (2×) and Et_2_O (2×), and the resin was dried *in vacuo*. The rest of the peptide was synthesised using the Biotage Alstra synthesiser (automated microwave peptide synthesiser) in a 30 mL reactor vial by swelling of Rink Amide ChemMatrix resin (298 mg, 0.2 mmol, 0.48 mmol g^−1^) in DCM at 40 °C for 30 min using orthogonally protected Fmoc-amino acids (0.15 M, 4 eq.) and 1-[bis(dimethylamino)methylene]-1*H*-1,2,3-triazolo[4,5-*b*]pyridinium 3-oxide hexafluorophosphate (0.5 M, 3.92 eq.) in DMF and *N*,*N*-diisopropylethylamine (2 M, 8 eq.) in NMP. After the last Fmoc-deprotection an *N*-acetylation was carried out using Ac_2_O (5 M, 40 eq.) in DMF and *N*,*N*-diisopropylethylamine (2 M, 8 eq.) in NMP. The purification was done by preparative HPLC using a Waters Atlantis T3 ODB column (5 μm, 19 × 150 mm) with a gradient of 5% B for 0.5 min, 5–21% B in 1.5 min, 21–26% B in 14 min (A: H_2_O/HOAc 100 : 0.5, B: MeCN) and a flow of 30 mL min^−1^ at room temperature. The volume injected was 650 μL with a concentration of 95 mg mL^−1^. Fractions were collected at 230 nm. Pure compound was identified by analytical HPLC with a Waters Acquity BEH C18 column (1.7 μm, 2.1 × 50 mm) using a linear gradient 5–60% in 9.3 min (A: H_2_O/TFA 100 : 0.1, B: MeCN/H_2_O/TFA 95 : 5 : 0.1) and a flow of 0.4 mL min^−1^ at 45 °C. Detection was done at 230 nm. The pure product (amount 42 mg, yield 6.6%, UV purity 97%) was found to be a white solid. ESI MS (+) (*m*/*z*): calculated for C_100_H_159_N_25_O_25_S = 1072.8, found = 1072.7 (M + 2H)^2+^ and ESI MS (−) (*m*/*z*): calculated for C_100_H_159_N_25_O_25_S = 715.5, found = 715.5 (M − 3H)^3−^.

#### PLP peptide NMR

All experiments were collected on a Bruker AVANCE NEO 600 MHz spectrometer equipped with a 5 mm detection cryoprobe (CP TCI H-C/N-D-05 Z). The PLP peptide was dissolved in two different solvents: 90%-H_2_O/10%-D_2_O and 80%-TFE/20%-H_2_O (v/v), at 1 mM concentration,^64^ due to observed aggregational behaviour at higher concentration in aqueous solution. Deuterated trifluoroethanol was obtained from Alfa Aesar, Karlsruhe Germany. For the experiments performed in aqueous solution, 3-(trimethylsilyl)-propionate-d4, sodium salt (TSP) was used as external standard;^65^ tetramethylsilane (TMS) was used as internal standard for the experiments in TFE/H_2_O. ^1^H 2D DQF-COSY, TOCSY, ROESY and HSQC were recorded at 298 K using the following Bruker pulse programs, cosydfesgpph, mlevesgpph, roesyesgpph and hsqcedetgpsisp2. ROESY spectra were recorded using 512 increments of 8 K data points and 64 scans per increment. A mixing time of 200 ms was used for the ROESY spectra and a mixing time of 120 ms was used for TOCSY spectra. Excitation sculpting sequence was used for water suppression in ^1^H 2D DQF-COSY, TOCSY and ROESY. Spectra were processed in Topspin 4.0.8. For ROESY spectra, t1 and t2 dimensions were both zero-filled respectively to 1 K and 8 K real data points, sine-bell squared window functions were applied in both dimensions. The spectra were baseline corrected, using Bernstein Polynomial and Polynomial fit methods, and analyzed in MestReNova 14.1.1.

## Results and discussion

### The PLP peptide

In order to assess the ability of different force field/solvent combinations to predict aqueous and membrane-associated peptide conformational ensembles, we chose the PLP peptide as a test case.^6^ As discussed in the introduction, the PLP peptide is part of the C-terminus of the PLP protein, which is a myelin protein that spans the membrane.^66^ The C-terminus resides on the cytoplasmic side of the membrane and has been shown to be a random coil in aqueous medium but to adopt structure in membrane-mimicking environments.^6,66^ Specifically, CD data indicate that the PLP peptide adopts an α-helix in TFE : water mixtures and a combination of α- and 3_10_-helices in the presence of lipid vesicles at low peptide concentrations (Table 1).^6^ The peptide is also shown to adopt a β-sheet structure in the presence of lipid vesicles at high peptide concentrations but this is not relevant for this study, since we simulate only one peptide in each system.^6^

**Table 1:** Secondary structure content of the PLP peptide with four force fields in three solvents, taken as a percentage of the DSSP assignment of all the residues in all the equilibrated trajectory frames, with error bars calculated from the two simulations conducted for each force field/solvent combination. For simplicity, the DSSP assignment is categorised into helical elements (H) containing α-, π- or 3/10-helices (although the majority of the helical sampling in this study was α-helical), β elements (B) containing isolated β-bridges or full β-ladders, and coil elements (C) containing hydrogen bonded turns, bends, loops and irregular elements. Standard errors are taken between the two simulations for each force field/solvent combination. The structural observations made from the NMR experiments (Fig. 3) and the CD experiments in ref. 6 are included for comparison

	% Secondary structure content			
	Water	TFE : water (∼8 : 2 vol%)	Chloroform : methanol : water (∼4 : 4 : 1 vol% (Amber); ∼4.1 : 5.5 : 0.4 vol% (CHARMM))	Lipid vesicles
ff14SB	H = 28.9 ± 2.1	H = 34.7 ± 5.0	H = 59.7 ± 0.2	—
	B = 1.3 ± 0.8	B = 0.6 ± 0.6	B = 0.0 ± 0.0	
	C = 69.7 ± 1.3	C = 64.7 ± 4.4	C = 35.4 ± 0.2	
ff14IDPSFF	H = 1.4 ± 0.7	H = 1.0 ± 0.0	H = 0.7 ± 0.3	—
	B = 25.4 ± 3.5	B = 7.7 ± 3.6	B = 8.0 ± 3.0	
	C = 73.2 ± 2.7	C = 91.3 ± 3.7	C = 91.4 ± 3.4	
CHARMM36	H = 7.9 ± 1.0	H = 58.8 ± 2.8	H = 64.9 ± 2.6	—
	B = 4.0 ± 0.1	B = 0.0 ± 0.0	B = 0.0 ± 0.0	
	C = 88.1 ± 1.0	C = 41.1 ± 2.8	C = 35.1 ± 2.6	
CHARMM36m	H = 13.3 ± 0.8	H = 52.9 ± 0.1	H = 57.9 ± 2.0	—
	B = 2.1 ± 0.5	B = 0.1 ± 0.0	B = 0.0 ± 0.0	
	C = 84.5 ± 0.3	C = 47.0 ± 0.1	C = 42.1 ± 2.0	
NMR (ROESY)	Conformationally heterogenous (possible presence of helical content)	Ordered helical structure	—	—
CD expt.^6^	Random coil with small amount of helicity	α-helix	—	α-/3_10_-helix

The PLP peptide was simulated using the REST2 protocol in three solvent systems, with four force fields (Table 1). The choice of TFE : water (∼8 : 2 vol%) and chloroform : methanol : water (∼4 : 4 : 1 vol%) as membrane-mimicking solvents was made because the use of these solvent mixtures for membrane–protein extraction, CD and/or NMR has precedent in the experimental literature.^6,67–70^ Hydrophobic solvents have also been used to mimic the low polarity of protein binding pockets for CD and NMR experiments of intrinsically disordered protein-binding peptides.^4,71^ Following initial simulations, it was observed that the chloroform : methanol : water (∼4 : 4 : 1 vol%) mixture separates into two phases when using the CGenFF/DH models (Fig. S1†) but not when using the GAFF models (Fig. S2†). The biphasic nature of the solvent is not necessarily incorrect, since inspection of the associated experimental phase diagram^72^ reveals that the ∼4 : 4 : 1 vol% ratio falls on the boundary between monophasic and biphasic behaviour. However, we chose to slightly alter the solvent ratio for the CGenFF/DH models to create a monophasic mixture, as this is more consistent with the solvent behaviour observed in NMR and CD experiments (as noted in ref. 69). Therefore, a monophasic ratio of ∼4 : 4 : 1 vol% was used for the GAFF solvent and a monophasic ratio of ∼4.1 : 5.5 : 0.4 vol% was used for the CGenFF/DH solvent (Fig. S3†).

NMR experiments were also conducted to investigate the conformational propensity of the PLP peptide in H_2_O : D_2_O (9 : 1 vol%) and TFE : H_2_O (8 : 2 vol%), in order to provide further experimental data to compare against our simulation results. Specifically, the H_2_O : D_2_O (9 : 1 vol%) NMR solvent was used to provide an experimental benchmark for the simulations in water, and the TFE : H_2_O (8 : 2 vol%) NMR solvent was used to provide an experimental benchmark for the simulations in TFE : water.

Given the vast amounts of simulation data produced in this study, the following sub-sections will firstly present the results from two of the PLP peptide simulations as examples, followed by a comparison with the results from the NMR experiments, before providing a concise comparison of the results from each force field/solvent combination with the NMR and literature CD data.^6^ Full analyses of each trajectory can be found in the ESI.†

#### Simulation analysis

**Fig. 1:**
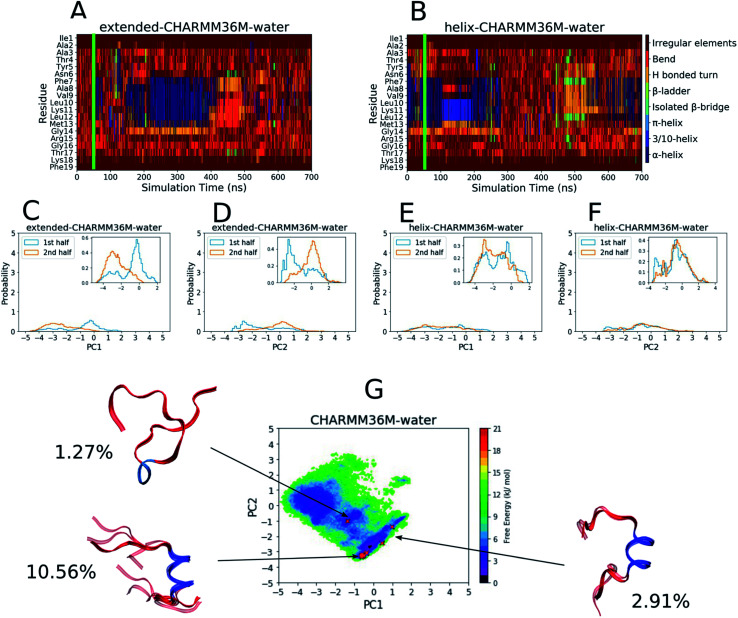
Results from the PLP peptide CHARMM36m water simulations. (A and B) DSSP analysis of the simulations starting from (A) an extended conformation and (B) a helical conformation. The green bar on each plot indicates the time from which equilibrium analysis was performed. (C–F) Histograms of the projections of the simulation coordinates onto PC1 (C and E) and PC2 (D and F) built from the first and second halves of the equilibrated part of the extended (C and D) and helical (E and F) trajectories. The *y* axes' maxima are set to 5 to allow comparison between all of the PLP peptide histograms (Fig. 2C–F and S19–S28C–F†), however zoomed in inserts are provided when the largest peak is less than 2. (G) The FES with respect to PC1 and PC2 built from the combined equilibrated parts of the two trajectories. The energy minimum is set to 0 and the colourbar range is fixed at 0–21 kJ mol^−1^ to allow comparison between all of the PLP peptide FESs (Fig. 2G and S19–S28G†). The DASH clusters are overlayed on the surface as red crosses, and those that occupy a similar PC and DSSP space are grouped into macrostates with the corresponding structural representatives shown as a superposition of the cluster centroids. The structures are shown as ribbons with random coil, turn and bend residues shown in red, α-helical residues shown in dark blue, 3_10_-helical residues shown in light blue, β-bridge residues shown in cyan and β-ladder residues shown in lime. The percentage of trajectory frames occupied by each macrostate is also shown.

Fig. 1 shows analyses of the PLP peptide simulations using the CHARMM36m force field in water. The figure includes results from the two simulations, one starting from an extended conformation and the other starting from a helical conformation. The two simulations were performed to assess convergence: if both simulations explored a similar conformational space after an initial equilibration period (determined using the criteria described in the methods), they were taken to be in equilibrium.

To define the conformational space, DSSP assignment and the first two PCs from dihedral backbone PCA were used. The DSSP assignment throughout both simulations can be seen in Fig. 1A and B with a green bar showing the point at which the equilibration periods end and from which the remaining analyses were performed. The plots show that the peptide mainly visited random coil structures but some helicity was occasionally observed between residues 7–13 in both simulations. The PC histograms in Fig. 1C–F show that, although the simulation starting from an extended conformation visited a different PC space in the first and second half of the trajectory, the full PC space covered by both trajectories was roughly similar, and the simulation starting from a helical conformation visited a similar PC space in both halves of the trajectory. This can also be seen in the DSSP plot (Fig. 1B), where the peptide unfolds and then refolds towards the end of the simulation. Although the exploration of conformational space is changing throughout both simulations, we believe that it is fair to end the equilibration periods at 50 ns to include both the random coil and helical sampling that is consistent in both simulations.

The FES with respect to the PLP peptide PC1 and PC2 coordinates was built from the combined equilibrated trajectory frames and can be seen in Fig. 1G, where there are several wide and shallow energy minima as a result of the diffuse sampling of PC space. The DASH clusters are labelled on the FES and grouped into macrostates based on their DSSP assignment and PC values, and the percentage of trajectory frames that each macrostate represents is shown. The macrostate structures are shown on the FES as overlayed DASH cluster centroids. Only about 15% of the trajectory frames belong to a given cluster, since most of the trajectory is occupied by disordered structures that are too sparsely populated for the DASH clustering algorithm to identify; for example the shallow energy minimum at [PC1 ∼ −2 to −4, PC2 ∼ −1 to 1] does not contain any clusters because this is a disordered region of PC space and the energy minimum is too diffuse to contain heavily populated states. The macrostates reside in or near the remaining two shallow FES minima, two of which have a central α-helix region (dark blue) with random coil residues (red) on either side, while the third is mainly random coil with a short 3_10_-helical region in the middle.

**Fig. 2:**
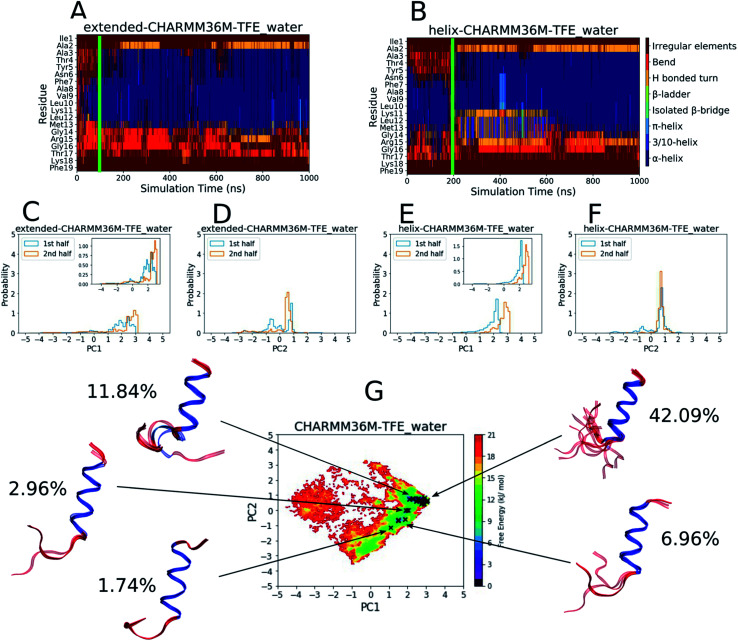
Results from the PLP peptide CHARMM36m TFE : water (∼8 : 2 vol%) simulations. (A and B) DSSP analysis of the simulations starting from (A) an extended conformation and (B) a helical conformation. The green bar on each plot indicates the time from which equilibrium analysis was performed. (C–F) Histograms of the projections of the simulation coordinates onto PC1 (C and E) and PC2 (D and F) built from the first and second halves of the equilibrated part of the extended (C and D) and helical (E and F) trajectories. The *y* axes maxima are set to 5 to allow comparison between all of the PLP peptide histograms (Fig. 1C–F and S19–S28C–F†), however zoomed in inserts are provided when the largest peak is less than 2. (G) The FES with respect to PC1 and PC2 built from the combined equilibrated parts of the two trajectories. The energy minimum is set to 0 and the colourbar range is fixed at 0–21 kJ mol^−1^ to allow comparison between all of the PLP peptide FESs (Fig. 1G and S19–S28G†). The DASH clusters are overlayed on the surface as black crosses, and those that occupy a similar PC and DSSP space are grouped into macrostates with the corresponding structural representatives shown as a superposition of the cluster centroids. The structures are shown as ribbons with random coil, turn and bend residues shown in red, α-helical residues shown in dark blue, 3_10_-helical residues shown in light blue, β-bridge residues shown in cyan and β-ladder residues shown in lime. The percentage of trajectory frames occupied by each macrostate is also shown.

As a comparison, the equivalent analyses of the PLP peptide simulations using the CHARMM36m force field in TFE : water (∼8 : 2 vol%) are shown in Fig. 2. In this case, simulation convergence was easier to establish because the DSSP plots (Fig. 2A and B) and PC histograms (Fig. 2C–F) have even greater similarity between the two simulations than those for the water simulations (Fig. 1A–F). The DSSP plots reveal a longer and more pronounced α-helix that was maintained throughout most of both simulations. The FES in Fig. 2G is less diffuse and has a more-defined and deeper minimum at [PC1 ∼ 2.8, PC2 ∼ 0.6], where longer helical conformations reside, compared to that in water (Fig. 1G). 21 DASH clusters were identified and grouped into five macrostates. In this case, the DASH clusters/macrostates represent a total of about 65% of the trajectory, showing that a higher percentage of the trajectory is occupied by well-defined structures, compared to the 15% in water. The macrostate structures have longer α-helical regions and fewer random coil residues than those found in water.

The results in Fig. 1 and 2 show that the CHARMM36m PLP peptide adopted longer and more-populated α-helical structures in TFE : water than in pure water. Although the water simulations still predicted some weak and short-lived helicity, the overall trend is consistent with the observation taken from experimental CD: the PLP peptide increases in helicity in the membrane-mimicking environment.^6^ These results suggest that the CGenFF TFE : water solvent provides a good membrane-mimic for CHARMM36m simulations.

#### NMR results

**Fig. 3:**
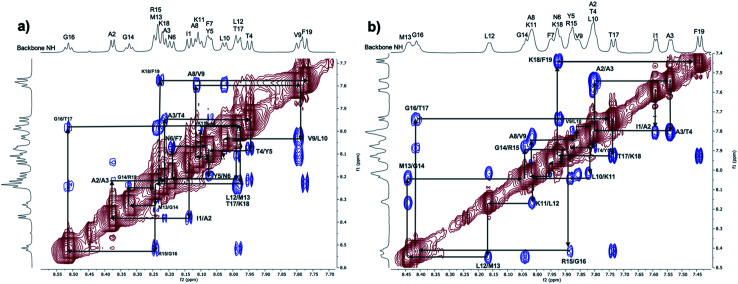
ROESY spectra (normalised by largest peak, value: 100) at 298 K: expansion of the NH/NH region for the 1 mM PLP peptide sample in aqueous solution (a) and in mixed solvent TFE/H_2_O (b).

Fig. 3 shows an expansion of the NH/NH region of the ROESY spectra obtained for the PLP peptide in aqueous solution (a) and in TFE/H_2_O mixture (b). In both solvents, from the analysis of the amide proton regions, *d*_NN_ ROE connectivities can be observed and were used for the sequential walk assignment (Fig. S12/Table S4 and Fig. S17/Table S6†). *d*_NN_ ROE connectivities, even if not always sufficient, are necessary evidence of helical content.^73^ In the TFE/H_2_O mixture, all of the amide protons have observed *d*_NN_ ROE connectivities with strong to medium intensities (Fig. 3b), indicating a uniform conformational behaviour and suggestive of helical content. For the peptide in aqueous solution, even though *d*_NN_ ROE connectivities are present, their intensities are more varied; weak, medium and strong intensity ROE can be observed (Fig. 3a). This suggests a greater conformational heterogeneity of the peptide in water.^74^ Temperature coefficients (Δ*δ*/Δ*T*) were also calculated for the amide protons of the peptide in aqueous solution (Fig. S14/Table S5†). High absolute values of Δ*δ*/Δ*T* were found, suggesting that none of the amide protons are involved in stable intramolecular H-bonds^75^ and providing supporting evidence for the greater conformational heterogeneity of the peptide in H_2_O/D_2_O.

To more closely compare the simulation results with the ROESY spectra, distance heat maps between the backbone amide protons from the CHARMM36m simulations in water and TFE : water are shown in the ESI (Fig. S4–S7†), revealing the presence of a more ordered signal for the PLP peptide in TFE : water compared to water. The presence of a strong *i* to *i* + 3 signal in the TFE : water heat map (Fig. S5†), along with the associated single peak histograms (Fig. S7†), results from the long and stable helix that is sampled, and this corresponds to the strong and ordered *d*_NN_ connectivities in the TFE : water ROESY spectrum (Fig. 3b). However, the presence of a weak *i* to *i* + 3 signal in the water heat map (Fig. S4†), along with the associated multiple peak histograms (Fig. S6†), suggests that a short and unstable helix is sampled as well as other disordered states, and this is in agreement with the conformational heterogeneity suggested by the *d*_NN_ connectivities observed in the water ROESY spectrum (Fig. 3a) and the high absolute values of the temperature coefficients, Δ*δ*/Δ*T* (Fig. S14/Table S5†).

An analysis of ROE correlations in the NH/aliphatic proton regions (Fig. S13 and S18†) was also performed for the PLP peptide in both aqueous solution and TFE/H_2_O. ROEs due to sequential and medium-range distances, like *d*_αN_ (*i*, *i* + 3) and *d*_αN_ (*i*, *i* + 4), are observable for the PLP peptide in TFE/H_2_O, whereas only ROEs due to sequential distances are present for the peptide in aqueous solution, except for a few, weak correlations for some central residues. These regions of the ROESY spectra can be compared directly with the simulations using the distance heat maps shown in Fig. S8 and S9† that show the interactions between the backbone NH and aliphatic protons found in the CHARMM36m simulations of the PLP peptide in water and TFE : water. The heat maps correlate well with the experimental ROESY: the presence of a strong, medium-range signal (*d*_αN_ (*i*, *i* + 3) and *d*_αN_ (*i*, *i* + 4)) can be seen in TFE : water, where a helical structure is present, and only a weak, medium-range signal can be observed for the central residues of the peptide in water, where a short-lived helix is formed in the simulation.

Overall, the observations made by NMR, along with the comparisons made between the ROESY spectra and the simulation heat maps, are consistent with what has been observed in the previously reported CD data^6^ and the CHARMM36m simulations discussed in the previous section (Fig. 1 and 2), confirming that the PLP peptide does indeed adopt a more ordered helix in the membrane-mimicking than the aqueous environment.

#### Comparison of solvent models and force fields with CD and NMR experiments

The simulation analyses shown in Fig. 1 and 2 were applied to each force field/solvent combination and are reported in the ESI (Fig. S19–S28†). Table 1 shows a concise evaluation of the PLP peptide results, with the secondary structure content of each combination taken as a percentage of the DSSP assignment of all the residues in all the equilibrated trajectory frames.

Inspection of the percentage secondary structure content predicted by the ff14SB force field shows that the PLP peptide has a greater helical propensity in chloroform : methanol : water than in water (59.7 ± 0.2% as opposed to 28.9 ± 2.1%, respectively) (Table 1). However, the helical content of the ff14SB PLP peptide only very slightly (and with low statistical certainty due to the overlapping error bars) increased in TFE : water (34.7 ± 5.0%), which does not reflect the structural distinction found between the aqueous and membrane-mimicking solvents in the NMR and CD experiments (Fig. 3).^6^ The absence of this distinction in our simulations is likely to be a consequence of the known oversampling of secondary structures by the ff14SB force field, due to the fact that generic protein force fields (including the Amber series) have historically been parameterised using globular protein structures,^44^ meaning that our water simulations were unable to sufficiently sample the disordered states.

The ff14IDPSFF force field, which was developed to add a CMAP correction term to ff14SB to remove the bias towards globular structures and increase the sampling of intrinsically disordered structures,^44^ performed badly for the PLP peptide in the given solvents (Table 1). A fairly significant β-ladder content was observed for the peptide in water (25.4 ± 3.5%) and the random coil content actually increased from 73.2 ± 2.7% to ∼91.0 ± 4.0% in the membrane-mimicking solvents, which is inconsistent with the experimental NMR and CD data (Fig. 3).^6^ There are no significant helical structures found in any of the simulations. Although ff14IDPSFF was developed to allow greater sampling of IDP structures, our results using ff14SB and ff14IDPSFF in water reveal that the CMAP term actually biased the sampling towards β-ladder structures and the random coil content remained roughly the same (Table 1). A closer look at the ff14IDPSFF parameterisation procedure reveals that parameters were fitted to coil/loop regions of protein crystal structures,^44^ which may not be appropriate for the description of standalone intrinsically disordered peptides that do not have a structured protein environment. Furthermore, X-ray crystallography captures static structures that may not be the most appropriate way to model dynamic coil and loop regions of proteins. Static crystal structures of dynamic protein regions are often not well defined.^76^

The CHARMM36 and CHARMM36m results were comparable, with both force fields correlating well with the experimental NMR and CD data (Table 1). The secondary structure content in water was mainly random coil (88.1 ± 1.0% for CHARMM36 and 84.5 ± 0.3% for CHARMM36m) and mainly helical in TFE : water (58.8 ± 2.8% for CHARMM36 and 52.9 ± 0.1% for CHARMM36m) and chloroform : methanol : water (64.9 ± 2.6% for CHARMM36 and 57.9 ± 2.0% for CHARMM36m). CHARMM36 was developed as a correction to the CHARMM22/CMAP force field for better protein backbone and side chain sampling,^42^ and CHARMM36m provided a further refinement to the CMAP potential for better sampling of IDP structures.^38^ This is noticeable in the membrane-mimicking solvents, where there was a slightly greater random coil content predicted by CHARMM36m compared to CHARMM36. Despite this, it is pleasing that CHARMM36m was able to correctly sample disordered structures and weak helicity of the PLP peptide in water and a more-ordered α-helix in the membrane-mimicking solvents. Additional radius of gyration (*R*(*g*)) and root mean square deviation (RMSD) analyses of the simulations of the PLP peptide in water and TFE–water with the CHARMM36m force field are provided in Fig. S43 and S44.†

At this stage, it is necessary to note that conformation-dependent replica trapping was observed as an effect of using the REST2 protocol in the membrane-mimicking solvents, and was most noticeable when using the CHARMM force fields. An example of the phenomenon is explained in more detail in the ESI (ESI Section: Replica Trapping Effect; Fig. S41 and S42†) but, briefly, we observed a reduction in the ability of the base replica to travel across the full replica space upon folding of the PLP peptide in the membrane-mimicking solvents. While it is important to consider that this effect reduces the conformational sampling in the simulation, we believe that the simulation procedure still provides a useful approach for the investigation of membrane-associated peptide conformations because the conformational sampling before the system relaxed to a folded structure was sufficient. Furthermore, preliminary simulations revealed that using hydrophobic solvent systems as membrane-mimicking models, rather than phospholipid bilayers, reduced the replica trapping effect and increased conformational sampling. Using REST2 with hydrophobic solvent models, therefore, provides an effective compromise between membrane-model accuracy and conformational sampling.

In summary, the PLP benchmark study revealed that REST2 can be used with certain force fields and membrane-mimicking solvents to reproduce membrane-associated IDP conformations. ff14IDPSFF had the worst correlation with the NMR and CD experiments, ff14SB behaved reasonably well with the chloroform : methanol : water solvent, but less well in water or TFE : water, and CHARMM36/CHARMM36m showed the best correlation with the experimental data. Considering that conformational prediction simulations of membrane-active peptides are likely to be used as a precursor to simulations that involve lipid bilayers, CHARMM36m is the better choice of force field because both its protein and lipid parameters were tested with the CHARMM modified TIP3P water model.^77^ CHARMM36, however, could be problematic for peptide–lipid simulations because its protein parameters were shown to work best with the standard TIP3P model, while the lipid parameters were parameterised using the CHARMM modified TIP3P model,^78^ meaning it could be difficult to choose an appropriate water model for simulations that include both peptides and lipids.

For these reasons, we chose to use the ff14SB and CHARMM36m force fields to study TP2 and ONEG (discussed in the next section).

### TP2 and ONEG

Having established a satisfactory protocol for investigating the environment-specific effects on the structural dynamics of the disordered PLP peptide, we next extend our investigation to a CPP called TP2.^46^ TP2 was identified in a high-throughput screen (HTS) as a spontaneous membrane translocating peptide (SMTP) that penetrates through artificial and cell membranes.^46,79^ We also investigate ONEG, a peptide that has a sequence similar to that of TP2 and yet was found to not penetrate through artificial or cell membranes and this will serve as a negative control.^47^ We hope that the structural differences between the two peptides in aqueous and membrane-mimicking environments could help identify the properties that are important for SMTP activity.

The secondary structure of TP2 has previously been characterised in aqueous buffer and in the presence of POPC vesicles by CD, which showed a transition from mainly random coil in water to an unknown secondary structure as the peptide interacts with POPC.^47^ Oriented-CD (OCD) of TP2 in a multi-bilayer POPC stack has also been performed and resulted in the observation of a β-sheet-like spectrum.^47^ However, the authors of the characterisation study noted that TP2 binds to and translocates membranes in a concentration-independent manner, meaning that it is likely that TP2 acts as a monomer and is unlikely to form β-sheet oligomers during translocation.^47^ This raises uncertainty as to the functional structure of TP2, which is why we chose to study it using MD simulations; in contrast, ONEG has been characterised as a random coil in both aqueous and membrane environments.^47^

#### Comparison of solvent models and force fields with CD experiments

REST2 was performed using the ff14SB and CHARMM36m force fields in all three solvent systems (water, chloroform : methanol : water and TFE : water) for TP2 and ONEG (Table S1†). Full analyses of the results can be found in the ESI (Fig. S29–S40†), while a concise evaluation of the secondary structure content is given in Tables 2 and 3 for TP2 and ONEG respectively.

For TP2, ff14SB predicted a strong helical content in water (47.4 ± 0.6%) that actually decreased in TFE : water (23.7 ± 2.1%) and only increased very slightly in chloroform : methanol : water (51.9 ± 1.6%) (Table 2). This is inconsistent with the CD data that shows that the peptide is mainly a random coil in water but gets increasingly structured as it interacts with the membrane.^47^ No significant β structures were found with the ff14SB force field in any of the solvents, and this is not in agreement with the OCD data.^47^

**Table 2:** Secondary structure content of TP2 with two force fields in three solvents, taken as a percentage of the DSSP assignment of all the residues in all the equilibrated trajectory frames, with error bars calculated from the two simulations conducted for each force field/solvent combination. For simplicity, the DSSP assignment is categorised into helical elements (H) containing α-, π- or 3/10-helices (although the majority of the helical sampling in this study was α-helical), β elements (B) containing isolated β-bridges or full β-ladders, and coil elements (C) containing hydrogen bonded turns, bends, loops or irregular elements. Standard errors are taken between the two simulations for each force field/solvent combination. The experimental CD assignment taken from ref. 47 is included for comparison

	% Secondary structure content			
	Water	TFE : water (∼8 : 2 vol%)	Chloroform : methanol : water (∼4 : 4 : 1 vol% (Amber); ∼4.1 : 5.5 : 0.4 vol% (CHARMM))	POPC
Amber14SB	H = 47.4 ± 0.6	H = 23.7 ± 2.1	H = 51.9 ± 1.6	—
	B = 0.7 ± 0.1	B = 1.4 ± 0.8	B = 0.4 ± 0.4	
	C = 51.9 ± 0.7	C = 74.9 ± 1.3	C = 47.7 ± 1.2	
CHARMM36m	H = 11.3 ± 0.1	H = 22.3 ± 10.7	H = 32.2 ± 22.1	—
	B = 0.6 ± 0.1	B = 0.1 ± 0.1	B = 0.5 ± 0.4	
	C = 88.1 ± 0.0	C = 77.6 ± 10.7	C = 67.3 ± 21.6	
CD expt.^47^	Random coil with “small amount of unidentifiable secondary structure”	—	—	Increased secondary structure (CD)
				β-sheet-like (OCD)

The CHARMM36m force field predicted a mainly random coil structure in water (88.1 ± 0.0%) with a very small amount of helical content (11.3 ± 0.1%) and vanishingly small β content (0.6 ± 0.1%); this provided a better correlation with the TP2 experiments (Table 2).^47^ The helical structure increased to 22.3 ± 10.7% in TFE : water and 32.2 ± 22.1% in chloroform : methanol : water, which agrees with the observed increase in secondary structure as the peptide interacts with POPC in the CD study.^47^ However, the β content did not increase in either of the membrane-mimicking solvents, which does not agree with the β-sheet-like structure found by OCD.^47^ A discussion of this contradiction and what it might suggest for the translocation structure of TP2 is included later.

As can be seen in Table 2, the standard errors for the structural percentages of the CHARMM36m TP2 are large, which is due to the large differences in conformational sampling that is seen between the two independent simulations for these systems. This can be seen in Fig. S32–S34,† where it is obvious that the structural content of TP2 continues to change throughout the 1 μs simulations, suggesting that the simulations are not yet converged. With computational expense in mind, the decision was taken not to continue any of the simulations past 1 μs and, where conformational convergence could not be established, to set a standard equilibration cutoff of 100 ns. Despite the poor convergence and large error bars, it can be noted that the membrane-mimicking solvents stabilise the helical structure of the CHARMM36m TP2, as can be seen from the slower unfolding of the helix in Fig. S33B and S34B, compared to that in Fig. S32B.†

Comparing the results for TP2 with those for the PLP peptide highlights how different force fields appear to be suited to different biological systems. For example, although it is likely that Amber14SB overpredicted the PLP peptide helicity in water, an obvious increase in helicity occurred as the solvent changed to chloroform : methanol : water (Table 1). However, the same force field predicted similar helical contents for TP2 in both solvents. The ability of the force field to accurately predict a structural transition of one peptide but not another is a reminder that there is not a “best” generic force field, at least for biopolymers.

The results for ONEG (Table 3) appear to be less force-field-dependent and more closely aligned with the experimental CD data than for TP2. As expected, the peptide is mainly a random coil with both force fields in all three solvents. Despite the prediction of a small amount of helicity in water with the ff14SB force field (8.0 ± 2.10%), both ff14SB and CHARMM36m were successful in reproducing the experimental differences showing that ONEG is less structured than TP2 in aqueous and membrane-mimicking environments.^47^ Additional *R*(*g*) and RMSD analyses of the simulations of TP2 and ONEG in water and TFE–water with the CHARMM36m force field are provided in Fig. S45–S48.†

**Table 3:** Secondary structure content of ONEG with two force fields in three solvents, taken as a percentage of the DSSP assignment of all the residues in all the equilibrated trajectory frames, with error bars calculated from the two simulations conducted for each force field/solvent combination. For simplicity, the DSSP assignment is categorised into helical elements (H) containing α-, π- or 3/10-helices (although the majority of the helical sampling in this study was α-helical), β elements (B) containing isolated β-bridges or full β-ladders, and coil elements (C) containing hydrogen bonded turns, bends, loops or irregular elements. Standard errors are taken between the two simulations for each force field/solvent combination. The experimental CD assignment taken from ref. 47 is included for comparison

	% Secondary structure content			
	Water	TFE : water (∼8 : 2 vol%)	Chloroform : methanol : water (∼4 : 4 : 1 vol% (Amber); ∼4.1 : 5.5 : 0.4 vol% (CHARMM))	POPC
Amber14SB	H = 8.0 ± 2.1	H = 2.4 ± 1.0	H = 2.8 ± 1.1	—
	B = 2.2 ± 0.3	B = 0.7 ± 0.4	B = 0.1 ± 0.0	
	C = 89.8 ± 1.8	C = 97.0 ± 1.4	C = 97.2 ± 1.0	
CHARMM36m	H = 0.4 ± 0.2	H = 0.4 ± 0.3	H = 3.0 ± 0.7	—
	B = 0.2 ± 0.0	B = 0.0 ± 0.0	B = 0.2 ± 0.2	
	C = 99.4 ± 0.2	C = 99.5 ± 0.3	C = 96.8 ± 0.9	
CD expt.^47^	Random coil	—	—	Random coil

#### Comments on the TP2 structure

It is already known experimentally that TP2 can penetrate through artificial and cellular membranes in a concentration-independent manner and without causing significant membrane disruption.^46^ This suggests that TP2 interacts favourably with the membrane as a monomer and partakes in a monomeric translocation pathway. The β-sheet-like spectrum of TP2 in POPC identified by OCD^47^ would suggest that the monomeric structure is a β-hairpin but we were unable to identify any significant β-hairpin structures in our simulations. In contrast, we consistently observed α-helical structures in the membrane-mimicking solvents.

Our helical observation is in agreement with implicit membrane simulations performed by Lazaridis *et al.* on TP2, which revealed that the helical conformation of the peptide in the membrane was characterised by a lower energy compared to an extended structure.^80^ They did, however, identify a low energy β-hairpin at the membrane interface and suggested that TP2 flips from a β-hairpin at the interface to an α-helix inside the membrane.^80^ It is possible that the β-sheet-like OCD spectrum of TP2 ^47^ is actually a measurement of the peptide at the membrane surface.

Alternatively, the β-sheet-like spectrum could result from unexpected TP2 aggregation in the OCD experiment. Macchi *et al.* investigated the effect of covalent cargo on the self-aggregation of TP2, showing that the critical micelle concentration (CMC) (the concentration above which aggregation occurs) increases when the fluorescent dye, TAMRA, is attached.^81^ TAMRA was covalently attached to TP2 in the translocation assays^46^ but not in the OCD experiments,^47^ so it could be possible that a monomeric TP2–TAMRA complex translocated in the cell penetration assays but a β-sheet TP2 oligomer formed in the OCD experiment. If this is the case, our monomeric simulations are relevant for TP2 below the CMC and it would be interesting to extend our simulation protocol to study peptide aggregation in the future.

Our hypothesis that TP2 adopts a helix inside the membrane gains further confidence because the peptide contains a specific motif made up of hydrophobic (Φ) and arginine (R) residues (ΦRΦΦR) that is also present in the S4 helix of the voltage-gated potassium channel, K_V_AP.^82^ Despite containing many positive charges, the S4 helix has been shown to move across the hydrophobic membrane during channel gating and to act as an SMTP as a stand-alone peptide.^82^ Studies have revealed the importance of this specific arrangement of hydrophobic and arginine residues in inducing SMTP behaviour,^83^ so it is reasonable to assume that TP2 has a similar structure and membrane-translocating mechanism as the S4 helix.

**Fig. 4:**
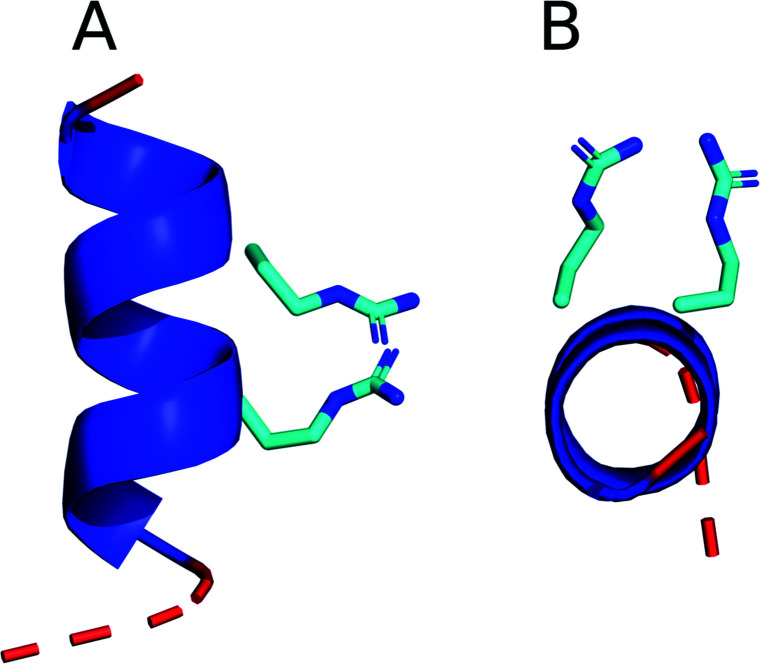
A structural cluster identified in the CHARMM36m TP2 simulation in chloroform : methanol : water, viewed from (A) the side of the helix and (B) down the axis of the helix. The backbone is represented as a cartoon with coil residues shown in red and α-helical residues shown in dark blue. The arginine side chains are shown in stick representation using the standard CPK colouring.

Furthermore, upon inspection of some of the TP2 structures identified in this study, it is obvious that the alignment of the two arginines in the α-helix allows the guanidinium groups to form a pair (Fig. 4). Guanidinium pairs are thermodynamically stable^84^ and are predicted to improve CPP activity because they result in non-additive membrane disruption, *i.e.* the overall water defect caused by a guanidinium pair moving into a membrane is less than that caused by two separate guanidinium groups.^84^ Additionally, the other TP2 residues are mainly hydrophobic, suggesting that the peptide amphipathicity is increased with the two positively charged residues pointing in the same direction, and this is known to be an important property for CPP activity.^85^ These observations support the theory that the TP2 monomer translocates as a helix.

In contrast, the simulation results suggest that ONEG does not form a stable secondary structure in the membrane, which is in agreement with the experimental CD and OCD data.^47^ The absence of a helical structure in ONEG could result from the presence of the central proline residue that disrupts the ability of the backbone to partake in ordered hydrogen bonding. The disordered structure of ONEG in the membrane would leave its polar backbone atoms open to the hydrophobic environment, which is unfavourable. Additionally, the inability of ONEG to form amphipathic secondary structures reduces its ability to orient itself to interact favourably with both the hydrophilic and hydrophobic parts of the membrane. Furthermore, ONEG has three arginine residues, as opposed to the two arginines present in TP2, and its disordered structure reduces its ability to form guanidinium pairs. To further test the hypothesis that helical structure is important for SMTP activity, it would be interesting to study the structures of the different hydrophobic/arginine motifs tested in ref. 83 to observe whether changing the arrangement of residues affects their ability to form stable, amphipathic helical structures and/or guanidinium pairs.

## Conclusion

IDPs are an important part of the proteome and are paramount in a range of biological processes including PPIs, the multimerisation of membrane proteins, amyloid aggregation, cell penetration and the disruption of bacterial membranes. The unique flexible nature of IDPs sensitises their conformational ensembles to the biological environment, enabling them to easily assume different functional conformations and bind to different macromolecules. For PPIs, this may involve the folding of an intrinsically disordered peptide upon binding to another protein, or for membrane-active peptides (such as membrane proteins, CPPs or AMPs), upon penetrating into the membrane. In both cases, the IDPs transition from an aqueous to a hydrophobic environment, resulting in a different conformational ensemble that allows them to perform their biological function.

MD is often used to study biological systems but it is challenging to simulate the conformational transitions of IDPs because of the long timescales required to sample all of the relevant disordered conformational space and to overcome the free energy barriers associated with peptide folding. In this paper, we tested the ability of four force fields to predict the conformational ensembles of aqueous and membrane-associated peptides, using the enhanced sampling method REST2 in aqueous and membrane-mimicking solvents, and conducted NMR experiments for the PLP peptide in two solvent systems to add information to be compared with the data obtained from the simulation protocol. There is also experimental precedent for using hydrophobic solvents to mimic the low polarity of protein binding sites,^4,71^ suggesting that our simulation protocol could be applicable for the conformational study of IDPs involved in PPIs. Furthermore, it would be interesting to use the protocol to simulate the conformational ensembles of cell-permeable macrocyclic drugs, which are also known to change their conformations as they pass through the hydrophobic cell membrane.^86^

We chose to benchmark the simulation protocol and force fields using the PLP peptide, which has relevant experimental CD data reported in the literature,^6^ and for which we produced additional experimental data with NMR. CD experiments have previously indicated that the PLP peptide is intrinsically disordered in the cytoplasm and binds to the membrane as an α- or 3_10_-helix.^6^ The NMR results presented in this paper suggest a more ordered helix in TFE/H_2_O than in H_2_O/D_2_O, which further validates the hypothesis that the peptide adopts a helical structure upon binding to the membrane. Out of the force fields tested in this study, we found that the CHARMM force fields were best able to capture the transition from disordered structures in water to helical structures in the membrane-mimicking solvents. It is likely that the ff14SB force field overstabilised the helix in water, however it was successful in capturing the transition to a more helical structure in chloroform : methanol : water, and the ff14IDPSFF force field was unable to predict a helical structure in the membrane-mimicking solvents. It would also be interesting to extend the simulation protocol to include multiple copies of the peptide in order to test the ability of the force fields to capture the transition from helical monomers to β-sheet oligomers observed in the CD experiments.^6^

Following the validation study on the PLP peptide, we applied the simulation protocol to TP2, an SMTP that is found to penetrate through artificial and cellular membranes at low concentrations. Our simulations led to an α-helical assignment of the peptide in the membrane, which contradicts the β-sheet-like OCD spectrum reported in the literature.^47^ We have included a discussion of this contradiction and have suggested mechanistic reasoning to support the possible α-helical assignment of TP2. Our simulations also predicted that the negative control peptide, ONEG, does not form a significant secondary structure, which could explain the difference in SMTP ability between the two peptides.

In conclusion, the results presented in this paper show that, with enhanced sampling and the right force fields, MD is a useful tool to study the conformational transitions of IDPs in different biological environments. We recommend the use of the CHARMM36m force field with the water, TFE : water or chloroform : methanol : water solvent models used in this study for the investigation of IDP conformations in aqueous and hydrophobic environments. Although we have observed conformation-dependent replica trapping with the use of the REST2 protocol in membrane-mimicking solvents, we note the proficiency of the method in enhancing the conformational sampling of IDPs without having to provide user-defined collective variables that may bias the simulation towards preconceived ideas of the peptide structure. Although we have tested the protocol on membrane-active peptides, the possible scope of this approach could extend to any IDP that undergoes environment-dependent structural transitions, and we hope to utilise this in the future to increase our understanding of the interesting mechanisms of IDPs.

## Data availability

Data for this paper, including simulation trajectory datasets, are available at Zenodo at https://doi.org/10.5281/zenodo.5830484.

## Author contributions

Lauren Reid: Investigation, Formal Analysis, Writing – original draft; Ileana Guzzetti: Investigation, Formal Analysis, Writing – original draft; Tor Svensson: Investigation; Anna-Carin Carlsson: Investigation; Wu Su: Conceptualisation; Supervision; Writing – review & editing; Tomas Leek: Conceptualization; Supervision; Writing – review & editing; Lena von Sydow: Investigation; Werngard Czechtizky: Funding acquisition; Conceptualisation; Writing – review & editing; Marija Miljak: Software; Chandra Verma: Supervision, Funding acquisition, Conceptualisation, Project Administration; Leonardo De Maria: Conceptualisation; Project Administration; Supervision; Writing – review & editing; Jonathan W. Essex: Supervision, Funding acquisition, Conceptualisation. Project Administration, Writing – review & editing.

## Conflicts of interest

JWE's research is part funded by AstraZeneca. LMR is currently employed by MedChemica Ltd.

## Supplementary Material

SC-013-D1SC03496K-s001
