# Supplementary material for: How well does molecular simulation reproduce environment-specific conformations of the intrinsically disordered peptides PLP, TP2 and ONEG?
Source: Chem Sci. 2022 Jan 20;13(7):1957–71. doi: 10.1039/d1sc03496k (PMC8848758; doi:10.1039/d1sc03496k)
Supplement: SC-013-D1SC03496K-s001 [file SC-013-D1SC03496K-s001.pdf]

Electronic Supplementary Material (ESI) for Chemical Science.
This journal is © The Royal Society of Chemistry 2022

How Well Does Molecular Simulation Reproduce Environment-Specific Conformations of the Intrinsically Disordered Peptides PLP, TP2 and ONEG?

# - Supporting Information

Table S1) System setups and simulation times

Peptide	Force field	Initial conf.	Solvent system: Water + neutralising Cl^- ions + Na^+ Cl^- ions at 0.15 M ionic conc.	Solvent system: TFE:Water (~ 8:2 vol%) + neutralising Cl^- ions	Solvent system: Chloroform: Methanol: Water (~ 4:4:1 vol% (Amber); ~4.1:5.5:0.4 vol% (CHARMM)) + neutralising Cl^- ions
**PLP**	**14SB**	**Extended**	1 PLP, 18769 water, 54 Na^+ , 56 Cl^-, 500 ns	1 PLP, 3587 TFE, 3962 waters, 2 Cl^- , 300 ns	1 PLP, 1746 chloroform, 3711 methanol, 2219 waters, 2 Cl^-, 300 ns
		**Helix**	1 PLP, 18629 water, 53 Na^+ , 55 Cl^-, 500 ns	1 PLP, 3533 TFE, 3967 waters, 2 Cl^-, 300 ns	1 PLP, 1724 chloroform, 3666 methanol, 2185 waters, 2 Cl^-, 300 ns
	**14SB-IDPS**	**Extended**	1 PLP, 18769 water, 54 Na^+ , 56 Cl^-, 300 ns	1 PLP, 3587 TFE, 3962 waters, 2 Cl^-, 300 ns	1 PLP, 1746 chloroform, 3711 methanol, 2219 waters, 2 Cl^-, 300 ns
		**Helix**	1 PLP, 18629 water, 53 Na^+, 55 Cl^-, 300 ns	1 PLP, 3533 TFE, 3967 waters, 2 Cl^-, 300 ns	1 PLP, 1724 chloroform, 3666 methanol, 2185 waters, 2 Cl^-, 300 ns

	**C36**	**Extended**	1 PLP, 18769 water, 54 Na^+, 56 Cl^-, 300 ns	1 PLP, 3545 TFE, 3421 waters, 2 Cl^-, 700 ns	1 PLP, 1488 chloroform, 4949 methanol, 932 waters, 2 Cl^-, 300 ns
		**Helix**	1 PLP, 18629 water, 53 Na^+, 55 Cl^-, 500 ns	1 PLP, 3533 TFE, 3343 waters, 2 Cl^-, 300 ns	1 PLP, 1470 chloroform, 4892 methanol, 920 waters, 2 Cl^-, 300 ns
	**C36M**	**Extended**	1 PLP, 18769 water, 54 Na^+, 56 Cl^-, 500 ns	1 PLP, 3044 TFE, 3425 waters, 2 Cl^-, 1000 ns	1 PLP, 1488 chloroform, 4949 methanol, 932 waters, 2 Cl^-, 1000 ns
		**Helix**	1 PLP, 18629 water, 53 Na^+, 55 Cl^-, 600 ns	1 PLP, 3533 TFE, 3343 waters, 2 Cl^-, 1000 ns	1 PLP, 1470 chloroform, 4892 methanol, 920 waters, 2 Cl^-, 300 ns
**TP2**	**14SB**	**Extended**	1 TP2, 7068 waters, 20 Na^+, 23 Cl^-, 300 ns	1 TP2, 1300 TFE, 1467 waters, 3 Cl^-, 700 ns	1 TP2, 595 chloroform, 1411 methanol, 893 waters, 3 Cl^-, 300 ns
		**Helix**	1 TP2, 6940 waters, 20 Na^+, 23 Cl^-, 300 ns	1 TP2, 1237 TFE, 1419 waters, 3 Cl^-, 700 ns	1 TP2, 586 chloroform, 1365 methanol, 852 waters, 3 Cl^-, 300 ns
	**C36M**	**Extended**	1 TP2, 7068 waters, 20 Na^+, 23 Cl^-, 1000 ns	1 TP2, 1292 TFE, 1230 waters, 3 Cl^-, 1000 ns	1 TP2, 591 chloroform, 1716 methanol, 350 waters, 3 Cl^-, 1000 ns
		**Helix**	1 TP2, 6807 waters, 19 Na^+, 22 Cl^-, 1000 ns	1 TP2, 1247 TFE, 1147 waters, 3 Cl^-, 1000 ns	1 TP2, 572 chloroform, 1624 methanol, 323 waters, 3 Cl^-, 1000 ns
**ONEG**	**14SB**	**Extended**	1 ONEG, 5611 waters, 16 Na^+, 20 Cl^-, 700 ns	1 ONEG, 992 TFE, 1191 waters, 4 Cl^-, 1000 ns	1 ONEG, 473 chloroform, 1103 methanol, 636 waters, 4 Cl^-, 300 ns
		**Helix**	1 ONEG, 6528 waters, 19 Na^+, 23 Cl^-, 700 ns	1 ONEG, 1196 TFE, 1376 waters, 4 Cl^-, 1000 ns	1 ONEG, 565 chloroform, 1311 methanol, 798 waters, 4 Cl^-, 300 ns

	**C36M**	**Extended**	1 ONEG, 5611 waters, 16 Na^+, 20 Cl^-, 300 ns	1 ONEG, 1001 TFE, 990 waters, 4 Cl^-, 300 ns	1 ONEG, 471 chloroform, 1342 methanol, 239 waters, 4 Cl^-, 700 ns
		**Helix**	1 ONEG, 6527 waters, 19 Na^+, 23 Cl^-, 300 ns	1 ONEG, 1214 TFE, 1148 waters, 4 Cl^-, 300 ns	1 ONEG, 562 chloroform, 1582 methanol, 309 waters, 4 Cl^-, 700 ns

REST2 Protocol

Replica exchange with solute scaling (REST2)[14] was used to enhance the conformational sampling of the peptides. The method involves simulating multiple replicas of the system in parallel with varyingly scaled Hamiltonians. The Hamiltonian of each replica is split into protein-protein (E_{pp}), protein-solvent (E_{ps}) and solvent-solvent (E_{ss}) terms, where the total energy of replica m is equivalent to:

$$E_m = \frac{\beta_m}{\beta_0} E_{pp} + \sqrt{\frac{\beta_m}{\beta_0}} E_{ps} + E_{ss} \qquad \textbf{Equation S1)}$$

where β_0 is $1/k_B T_0$ and β_m is $1/k_B T_m$, with T_0 being the base temperature of the system and T_m being the "effective temperature" of replica m. $T_0 = T_m$ in the base replica and T_m is scaled up in the higher order replicas; this has the effect of simulating a "hot" protein in a "cold" solvent [14]. Exchanges between replicas are attempted every 1ps, according to a Metropolis acceptance criteria with the delta term being equivalent to:

$$\Delta_{mn} = (\beta_m - \beta_n)\left[(E_{pp}(X_n) - E_{pp}(X_m)) + \frac{\sqrt{\beta_0}}{\sqrt{\beta_m}+\sqrt{\beta_n}}(E_{ps}(X_n) - E_{ps}(X_m))\right] \qquad \textbf{Equation S2)}$$

where X_n and $X_m$are the coordinates of replica n and m respectively. Thus, the base replica is able to "jump" over energy barriers and provides an unbiased ensemble of peptide conformations. The absence of the solvent-solvent term in the delta expression increases the efficiency of the exchanges compared to other replica exchange approaches [14].

Ten replicas with “effective temperatures” exponentially spaced between 300-600 K were used for the PLP peptide (**Table S2**), whereas eight replicas exponentially spaced between 300-600 K were used for the smaller TP2 and ONEG peptides (**Table S3**). In all cases, the peptide atoms were selected as the “hot” portion of the system and the remaining solvent and ions made up the “cold” solvent.

Table S2) Replicas used for the PLP peptide simulations

**Replica id**	0	1	2	3	4	5	6	7	8	9
**Effective temp/ K**	300.0	324.0	350.0	378.0	408.2	440.9	476.2	514.3	555.5	600.0

Table S3) Replicas used for the TP2 and ONEG simulations

**Replica id**	0	1	2	3	4	5	6	7
**Effective temp/ K**	300.0	331.2	365.7	403.8	445.8	492.2	543.4	600.0

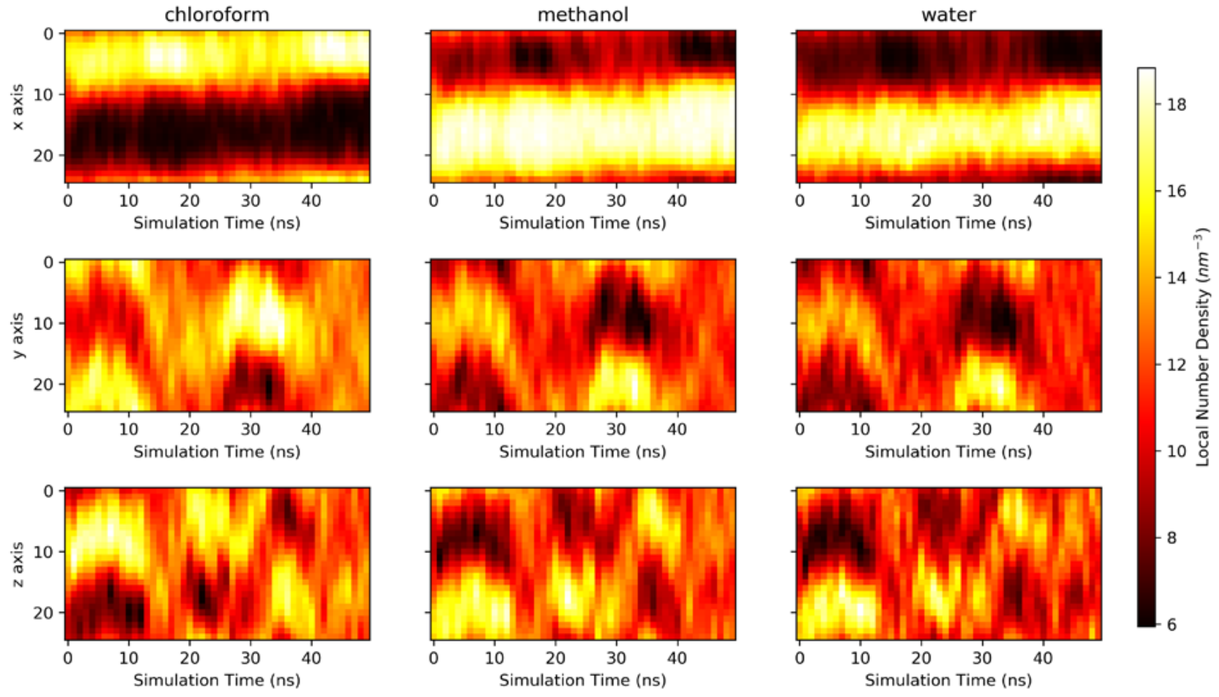

Figure S1) Local number density of chloroform, methanol and water in the x, y and z axes throughout a 50 ns simulation using the ~4:4:1 (vol%) chloroform:methanol:water ratio with the CGenFF/DH solvent models.

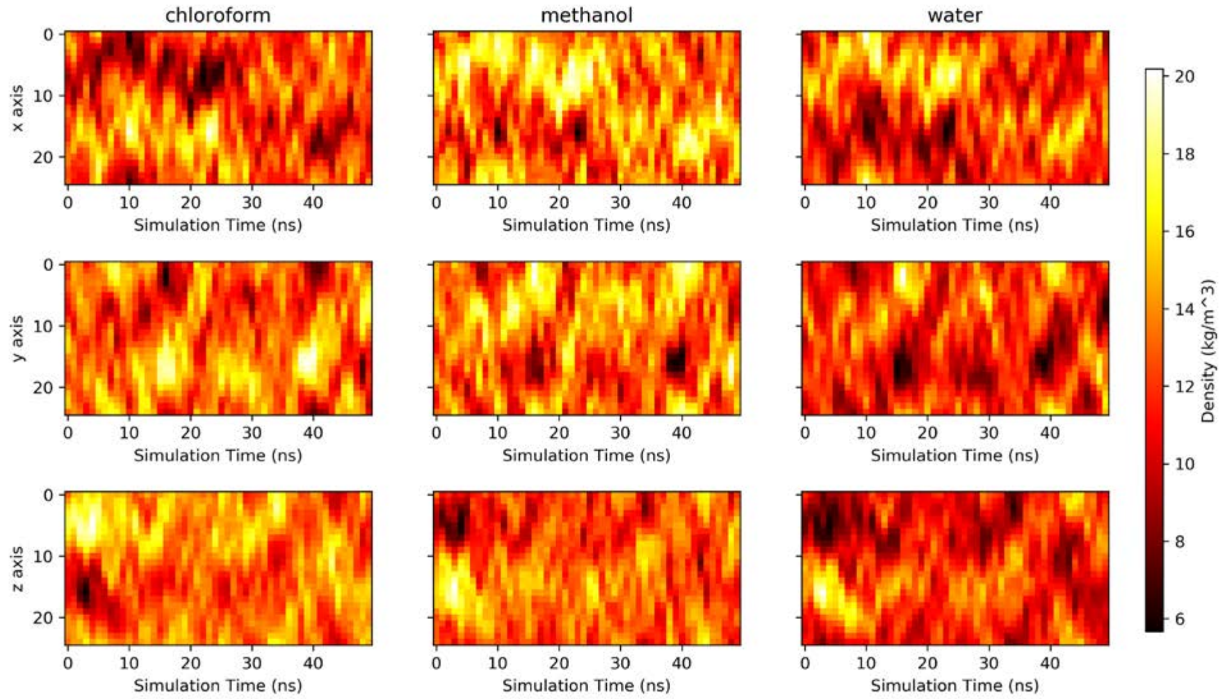

Figure S2) Local number density of chloroform, methanol and water in the x, y and z axes throughout a 50 ns simulation using the ~4:4:1 (vol%) chloroform:methanol:water ratio with the GAFF solvent models.

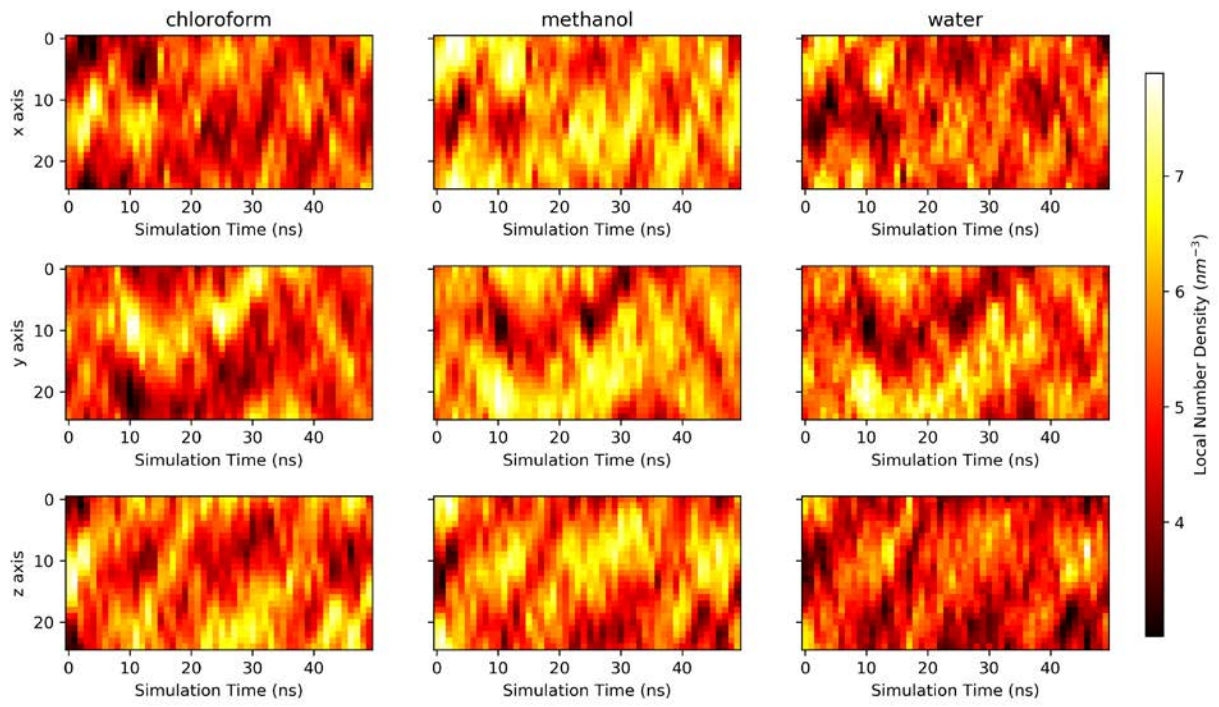

Figure S3) Local number density of chloroform, methanol and water in the x, y and z axes throughout a 50 ns simulation using the ~4.1:5.5:0.4 (vol%) chloroform:methanol:water ratio with the CGenFF/DH solvent models.

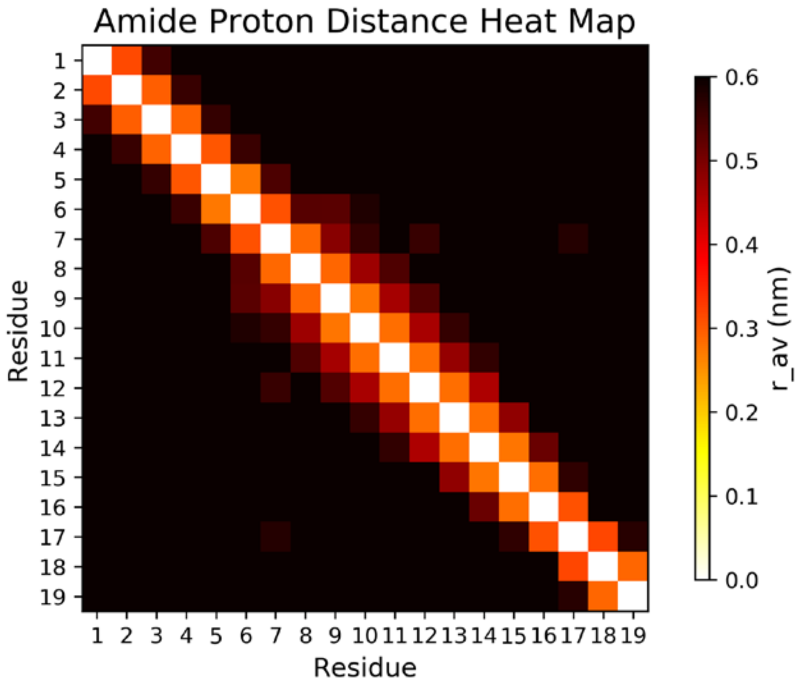

Figure S4) Heat map of the per-residue amide proton distances (r) averaged according to r_av = $<r^{-6}>^{(-1/6)}$ for the CHARMM36m simulation of the PLP peptide in water. Only r_av signals that are less than 0.6 nm are included. The weak i to i+3 signal in the central residues (6-11) suggests the presence of an unstable helix present in this region.

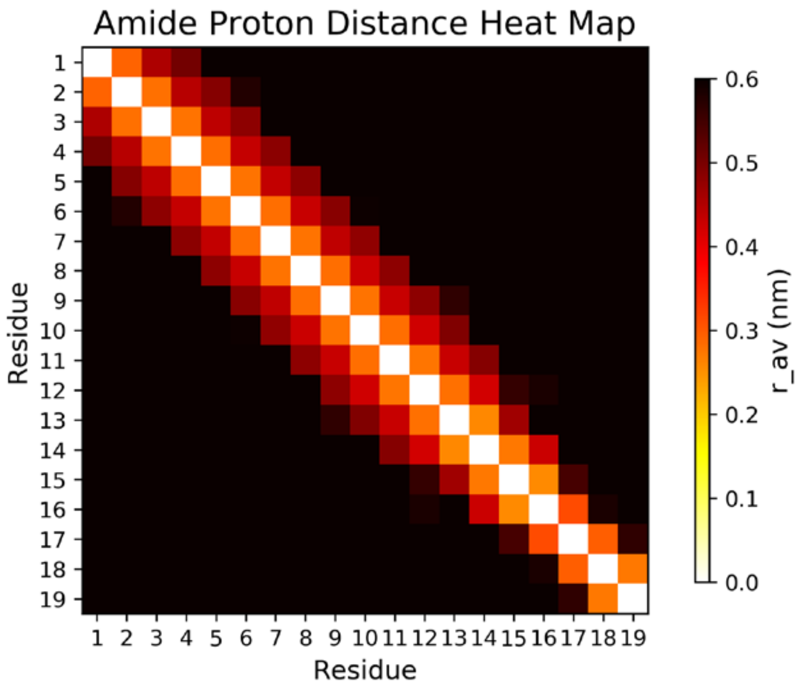

Figure S5) Heat map of the per-residue amide proton distances (r) averaged according to r_av = $<r^{-6}>^{(-1/6)}$ for the CHARMM36m simulation of the PLP peptide in TFE:water. Only r_av signals that are less than 0.6 nm are included. The strong i to i+3 signal in residues 1 to 12 suggests the presence of a stable helix in this region.

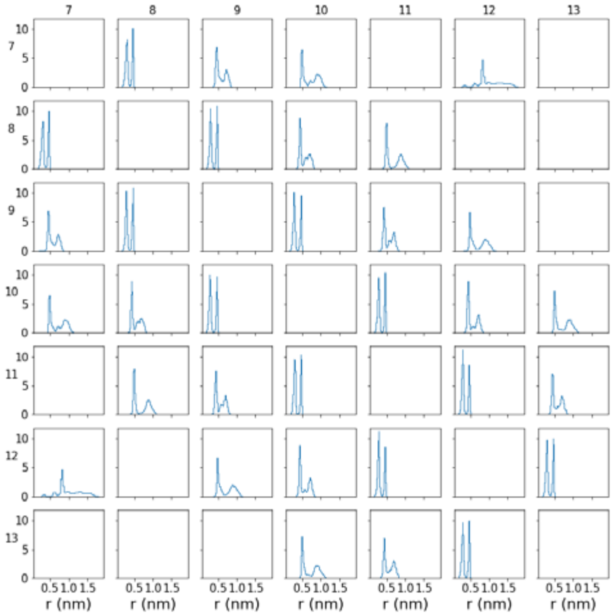

Figure S6) Histograms of the per-residue amide proton distances (r) for residues 7-13 for the CHARMM36m simulation of the PLP peptide in water. The presence of multiple peaks shows that the structure was heterogeneous.

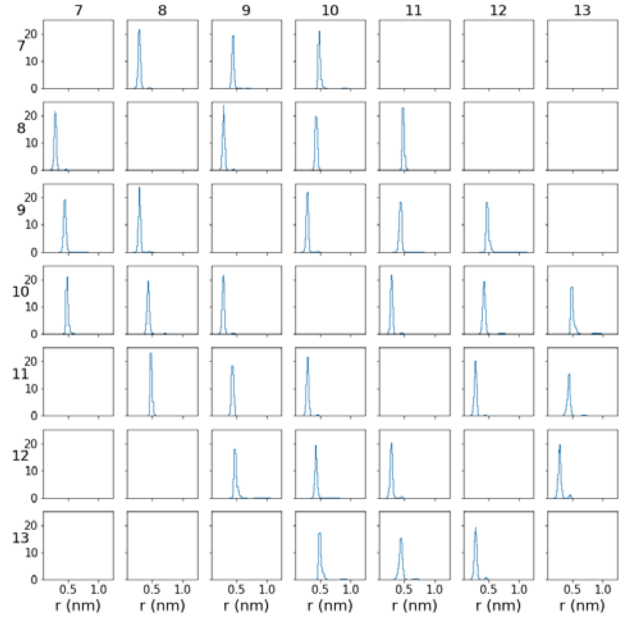

Figure S7) Histograms of the per-residue amide proton distances (r) for residues 7-13 for the CHARMM36m simulation of the PLP peptide in TFE:water. The presence of a single peak for most signals shows that the structure was ordered and stable.

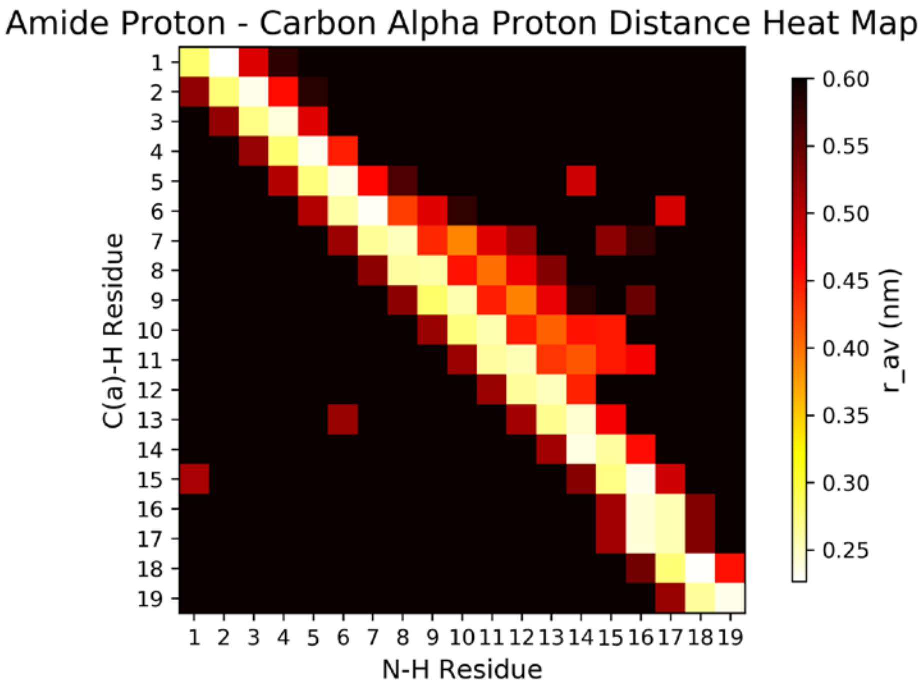

Figure S8) Heap map of the per-residue amide proton / alpha proton distances (r) averaged according to r_av = $<r^{-6}>^{(-1/6)}$ for the CHARMM36m simulation of the PLP peptide in water. Only r_av signals that are less than 0.6 nm are

included. The presence of medium-range interactions (dαN (i, i+3) and dαN (i, i+4)) can be observed for central residues only, where a weak and short-lived helix is occasionally visited.

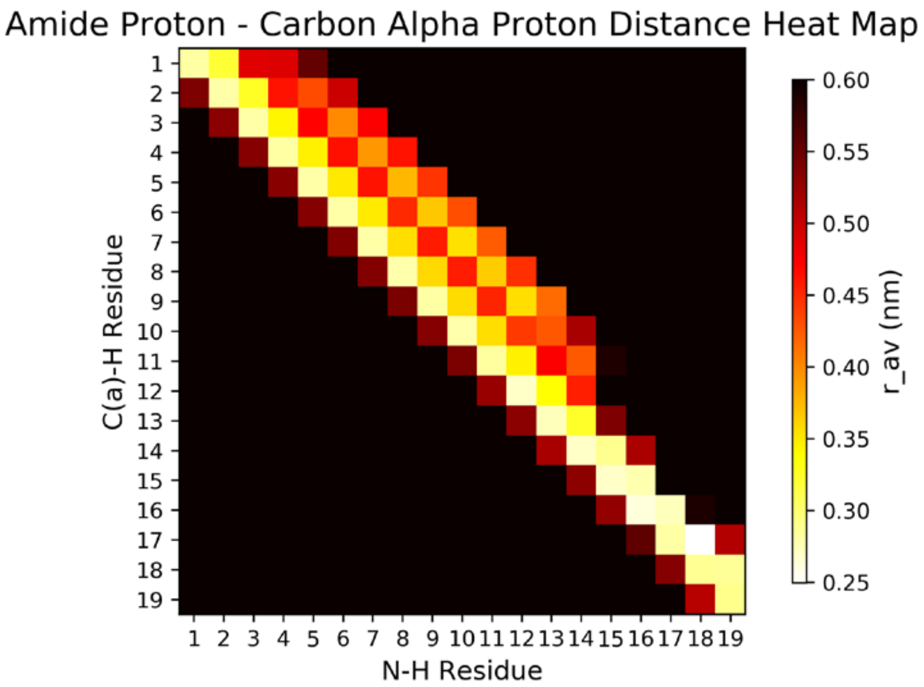

Figure S9) Heap map of the per-residue amide proton / alpha proton distances (r) averaged according to $r\_av = \langle r^{-6} \rangle^{(-1/6)}$ for the CHARMM36m simulation of the PLP peptide in TFE:water. Only r_av signals that are less than 0.6 nm are included. A strong presence of medium-range interactions (dαN (i, i+3) and dαN (i, i+4)) can be observed for residues 1-13, where a helix is present throughout most of the simulation.

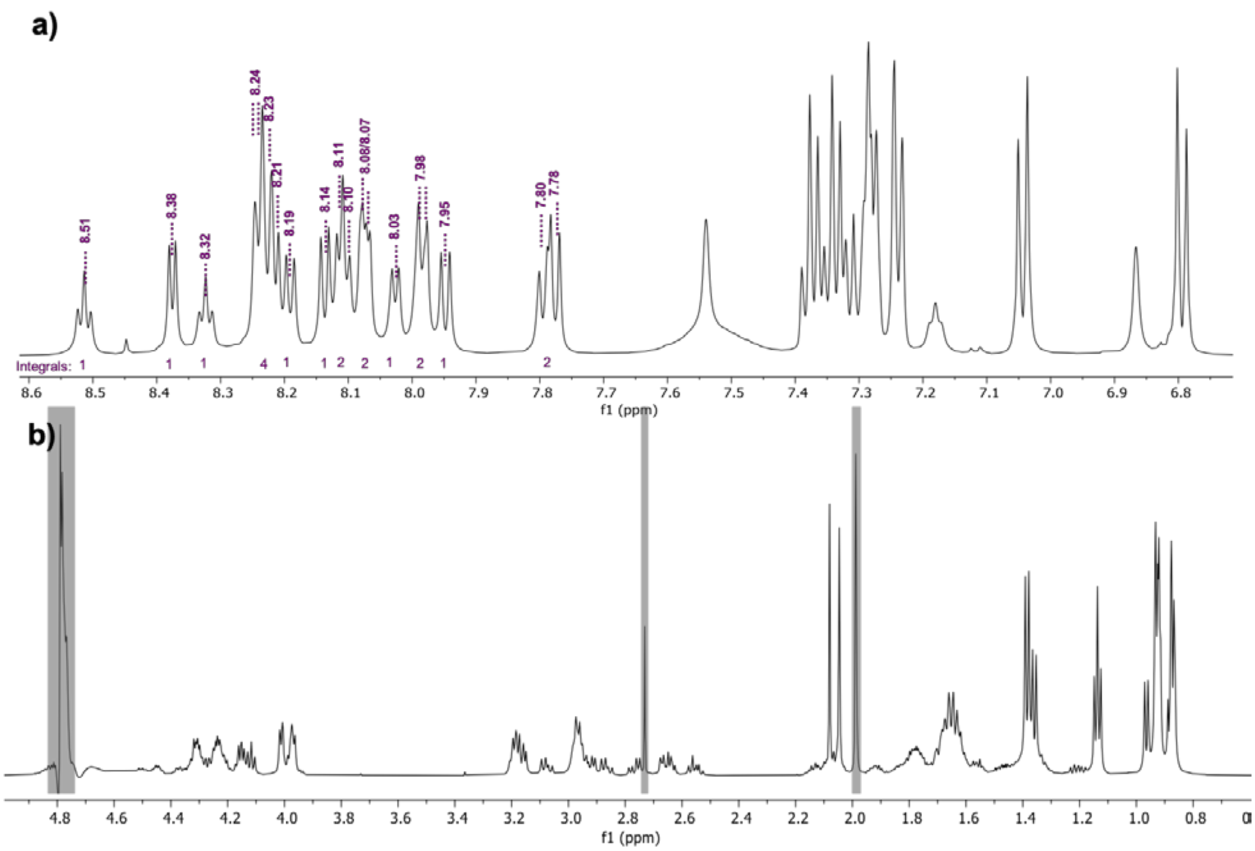

Figure S10) a) Expanded region (amide and aromatic protons) and **b)** expanded region (backbone and side chain aliphatic protons) of the ^{1}H NMR spectrum of the PLP peptide (1 mM) in 90%- H_2O /10%-D_2O (v/v) recorded at 600 MHz at 298 K. Impurities and solvent signals are highlighted in gray.

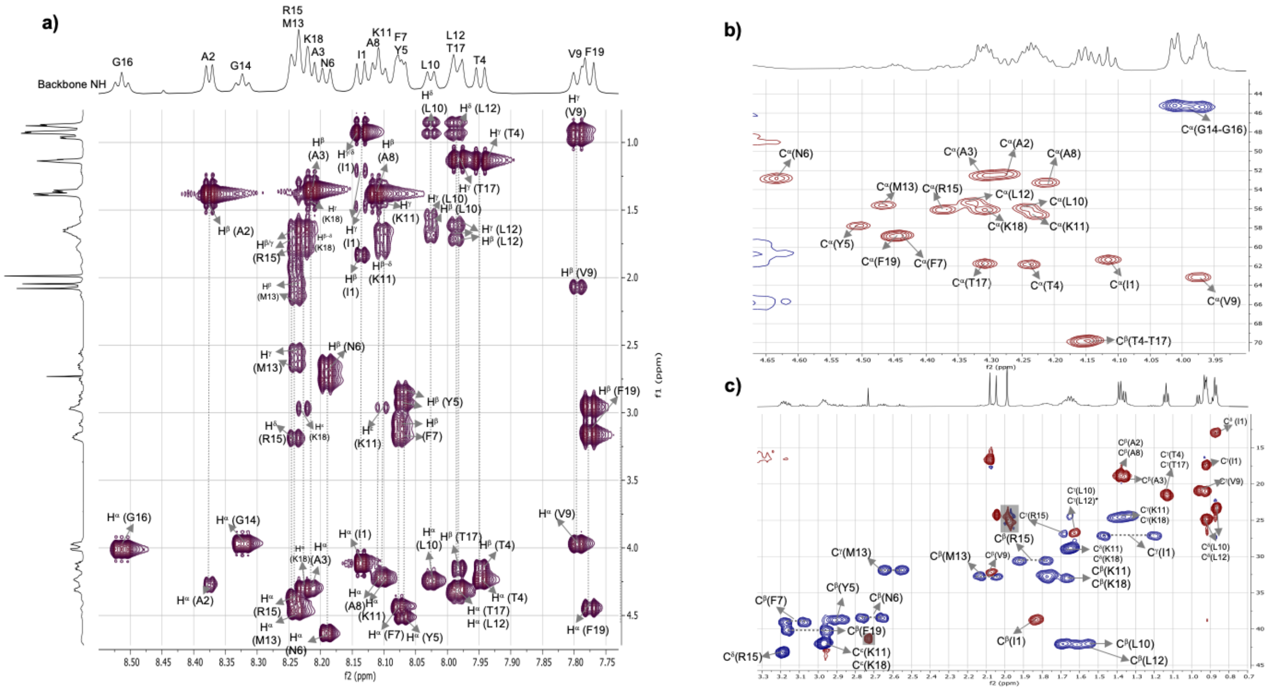

Figure S11) a) Expanded region (backbone NH) of the TOCSY spectrum with assigned spin systems for each amino acid; **b)** expanded region (Cα) and **c)** expanded region (side chain C) of HSQC spectrum of the PLP peptide (1 mM) in 90%- H_2O /10%-D_2O (v/v) recorded at 600 MHz at 298 K.

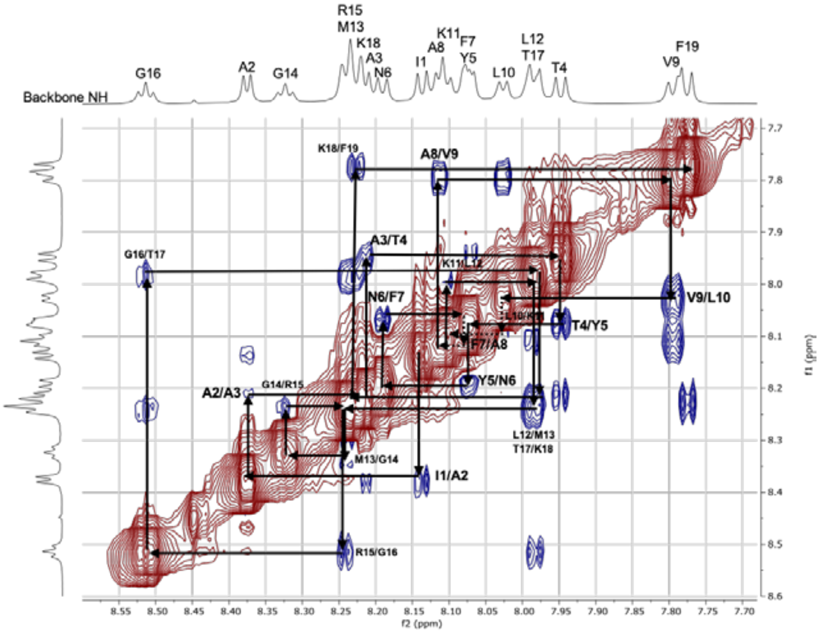

Figure S12) ROESY spectrum (NH/NH region) of the PLP peptide (1 mM) in 90%- H_2O/10%-D_2O (v/v) recorded at 600 MHz at 298 K. Sequential connectivities are shown by arrows from I1 to F19. ROESY cross-peaks between F7→A8 and L10→K11 are not observed (too close to diagonal) and are indicated by dashed arrows.

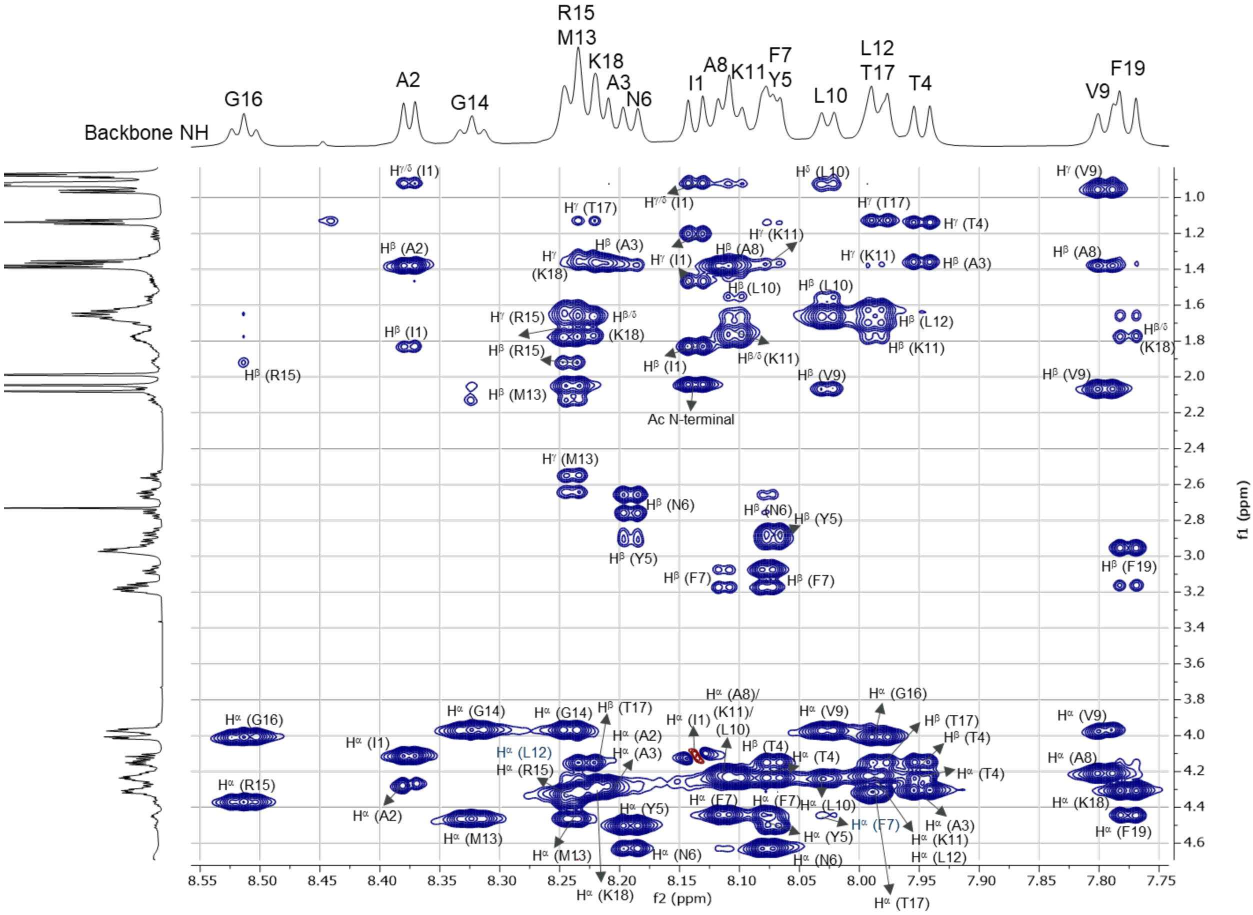

Figure S13) ROESY spectrum (NH/aliphatic ^{1}H region, normalised by largest peak, value: 100) of the PLP peptide (1 mM) in 90%- H_2O/10%-D_2O (v/v) recorded at 600 MHz at 298 K. In the expansion, just ROEs due to sequential distances are observed. ROEs due to medium-range backbone distances are not present, except for few and weak correlations for some central residues.

Residue	NH	H^{α} (C^{α})	H^{β} (C^{β})	Others
**I1**	8.14	4.11(61.4)	1.83(38.7)	1.46-1.21 (27.1) $C^{\gamma}H_2$<br>0.92** (17.4) $C^{\gamma}H_3$<br>0.87** (12.9) $C^{\delta}H_3$
**A2**	8.38	4.27(52.4***)	1.38*(18.7***)	--
**A3**	8.21	4.30*(52.6***)	1.36*(18.9)	--
**T4**	7.95	4.23*(61.7***)	4.14*(69.8***)	1.14**(21.5***) $C^{\gamma}H_3$
**Y5**	8.07	4.50(57.8)	2.93-2.85(38.7)	7.04-6.79(133.3-118.2) H_{Ar}
**N6**	8.19	4.63(52.7)	2.75-2.66(38.5)	7.54-6.86 NH_2
**F7**	8.08	4.44*(58.8)	3.16-3.08(39.1)	7.38/7.24** (129.6/132.1***) H_{Ar}
**A8**	8.11	4.22*(53.3)	1.38*(18.7***)	--
**V9**	7.80	3.97(63.1)	2.07(32.2)	0.97-0.91**(20.9-21.1***) $C^{\gamma}H_3$
**L10**	8.03	4.24*(55.9)	1.69-1.55*(42.0***)	--**(--***) $C^{\gamma}H$<br>0.93-0.85**(23.2-24.8***) $C^{\delta}H_3$
**K11**	8.10	4.22*(56.7***)	1.78-1.65*(33.0***)	1.37**(24.6***) $C^{\gamma}H_2$<br>1.65**(28.9***) $C^{\delta}H_2$<br>2.97**(42.0***) $C^{\epsilon}H_2$
**L12**	7.98*	4.31*(55.3)	1.71-1.62*(42.0***)	1.63**(26.8***) $C^{\gamma}H$<br>0.93-0.85**(23.2-24.8***) $C^{\gamma}H_3$
**M13**	8.24	4.47(55.7)	2.13-2.05(32.8)	2.65-2.54(31.8) $C^{\gamma}H_2$<br>2.08(16.6) $C^{\epsilon}H_3$
**G14**	8.32	3.97(45.3)	--	--
**R15**	8.24	4.37(56.1)	1.92-1.79(30.7)	3.19(43.2) $C^{\delta}H_2$<br>1.67**(26.9***) $C^{\gamma}H_2$<br>7.18 $N^{\epsilon}H$
**G16**	8.51	4.01(45.1)	--	--
**T17**	7.98*	4.31*(61.7***)	4.15*(69.8***)	1.12**(21.5***) $C^{\gamma}H_3$
**K18**	8.23	4.30*(56.1***)	1.8-1.78*(33.0)	1.37**(24.6***) $C^{\gamma}H_2$<br>1.65**(28.9***) $C^{\delta}H_2$<br>2.97**(42.0***) $C^{\epsilon}H_2$
**F19**	7.78	4.44(58.8***)	3.15-2.95(40.2)	7.38/7.24** (129.6/132.1***) H_{Ar}

Table S4) ^{1}H and ^{13}C resonance assignments (ppm) of the PLP peptide (1 mM) in 90%-H_2O/10%-D_2O (v/v) recorded at 600 MHz at 298 K.

*, ** overlapping of the ^{1}H δ, ^{13}C δ were assigned, as most probable, on the base of "random coil" values[1]

*** ^{13}C δ overlapping

-- not applicable or not possible to prove due to overlapping

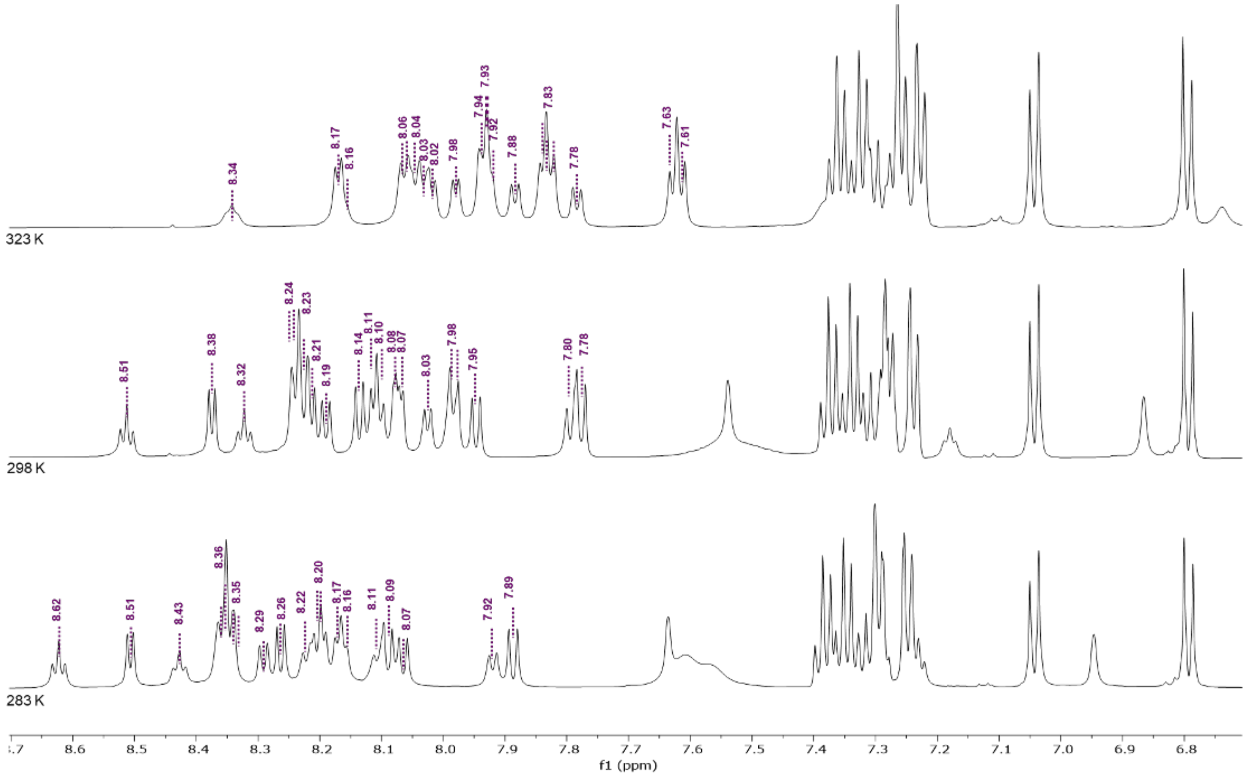

Figure S14) 1D ^1H spectra (expansion of NH and aromatic protons region) of the PLP peptide (1 mM) in 90%-H_2O/10%-D_2O (v/v) recorded at 600 MHz at three different temperatures: 283 K, 298 K and 323 K

Residue	283 K	298 K	323 K	Δδ/ΔT (ppb/K)
**I1**	8.26	8.14	7.94	-8.00
**A2**	8.51	8.38	8.17	-8.50
**A3**	8.35	8.21	8.02	-8.25
**T4**	8.07	7.95	7.78	-7.25
**Y5**	8.20	8.07	7.88	-8.00
**N6**	8.29	8.19	8.03	-6.50
**F7**	8.17	8.08	7.92	-6.25
**A8**	8.20	8.11	7.98	-5.50
**V9**	7.92	7.80	7.63	-7.25
**L10**	8.16	8.03	7.83	-8.25
**K11**	8.22	8.10	7.93	-7.25
**L12**	8.11	7.98*	7.83	-7.00
**M13**	8.36	8.24	8.06	-7.50
**G14**	8.43	8.32	8.16	-6.75
**R15**	8.36	8.24	8.06	-7.50
**G16**	8.62	8.51	8.34	-7.00
**T17**	8.09	7.98*	7.83	-6.50
**K18**	8.35	8.23	8.04	-7.75
**F19**	7.89	7.78	7.61	-7.00

Table S5) δ of the amide protons of the PLP peptide (1 mM) in 90%-H_2O/10%-D_2O (v/v) at 298 K and their corresponding temperature coefficients are reported.

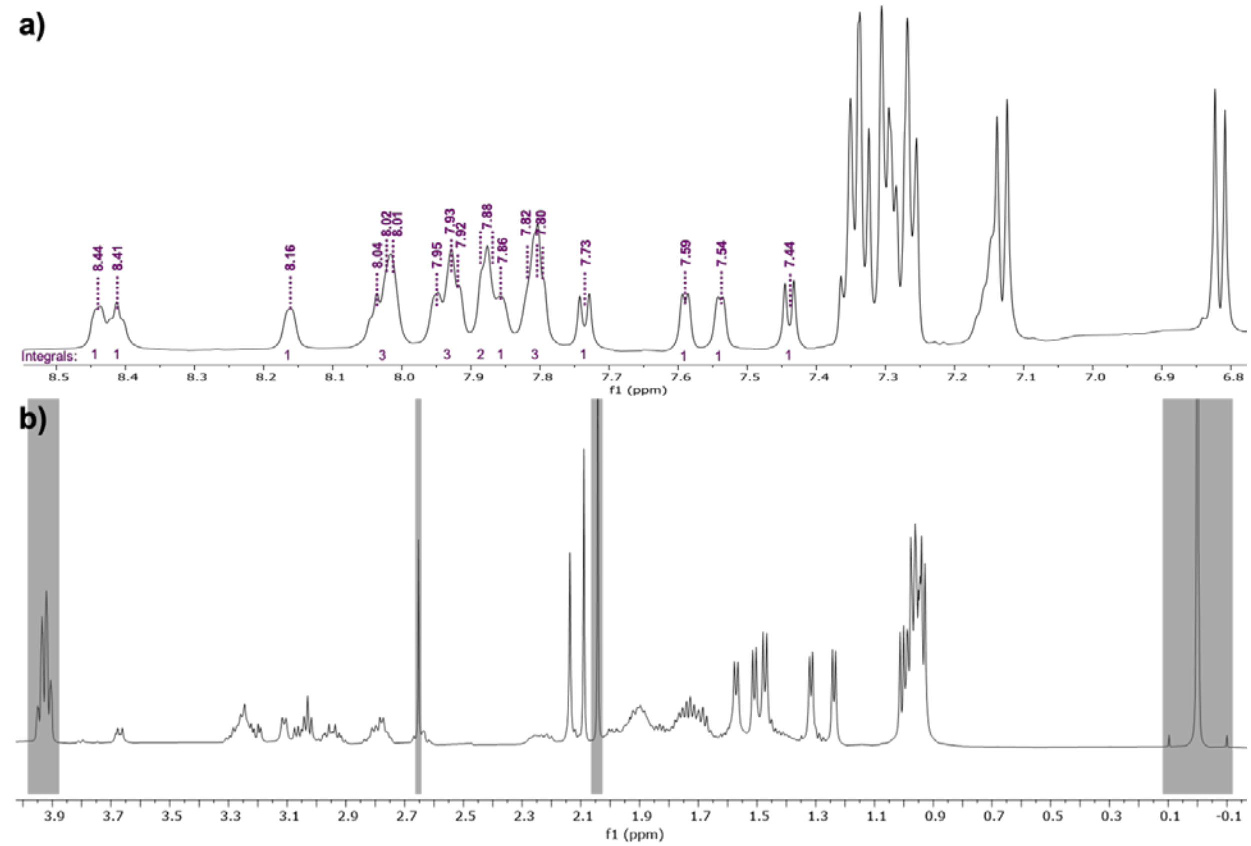

Figure S15) a) Expanded region (amide and aromatic protons) and **b)** expanded region (backbone and side chain aliphatic protons) of the ^{1}H NMR spectrum of the PLP peptide (1 mM) in 80%-TFE/20%-H_2O (v/v) recorded at 600 MHz at 298 K. Impurities, solvent signals and reference are highlighted in gray.

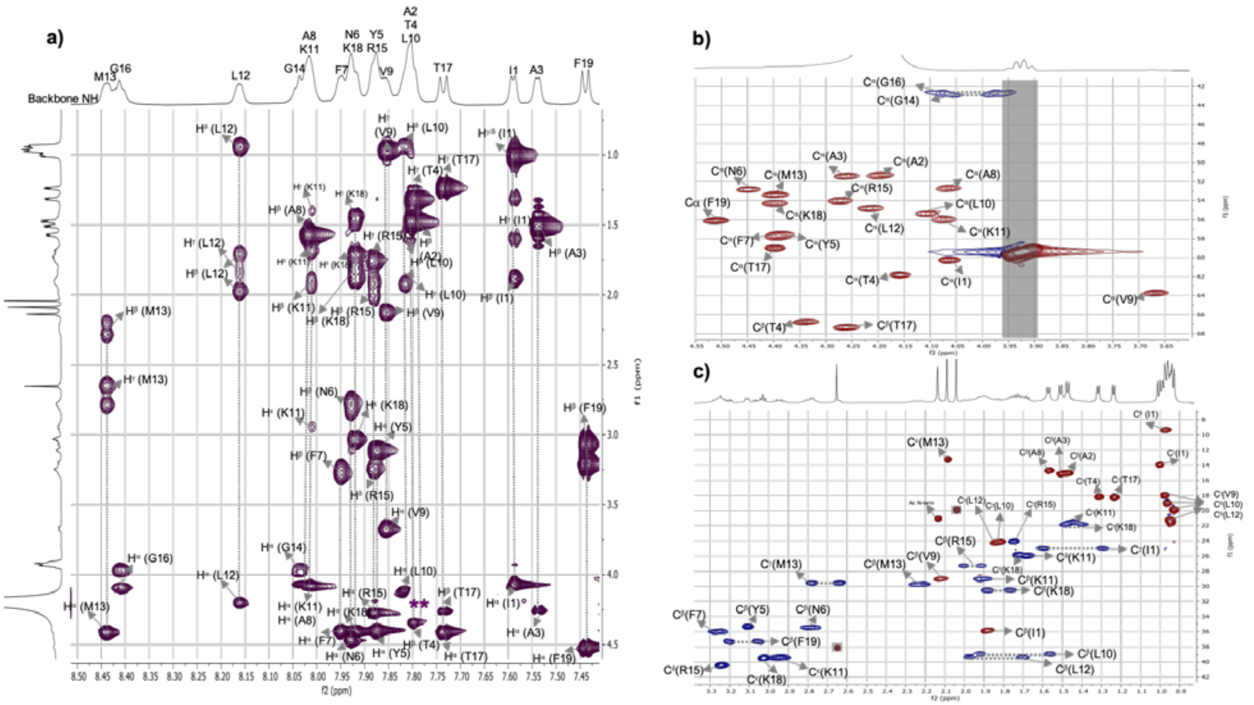

Figure S16) a) Expanded region (backbone NH) of the TOCSY spectrum with assigned spin systems for each amino acid; ** H$^{\alpha}$ under water signal, observed in COSY spectrum; **b)** expanded region (C$^{\alpha}$) and **c)** expanded region (side chain C) of HSQC spectrum of the PLP peptide (1 mM) in 80%-TFE/20%-H_2O (v/v) recorded at 600 MHz at 298 K.

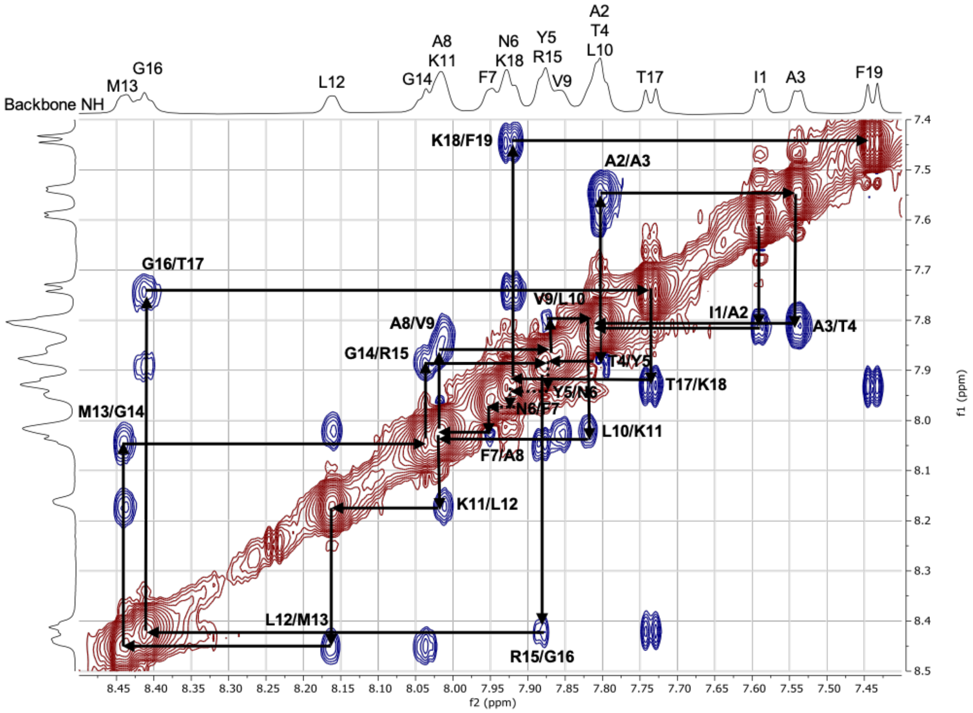

Figure S17) ROESY spectrum (NH/NH region) of the PLP peptide (1 mM) in 80%-TFE/20%-H_2O (v/v) recorded at 600 MHz at 298 K. Sequential connectivities are shown by arrows from I1 to F19. ROESY cross-peaks between Y5→N6→F7 are not observed (too close to diagonal) and are indicated by dashed arrows**.**

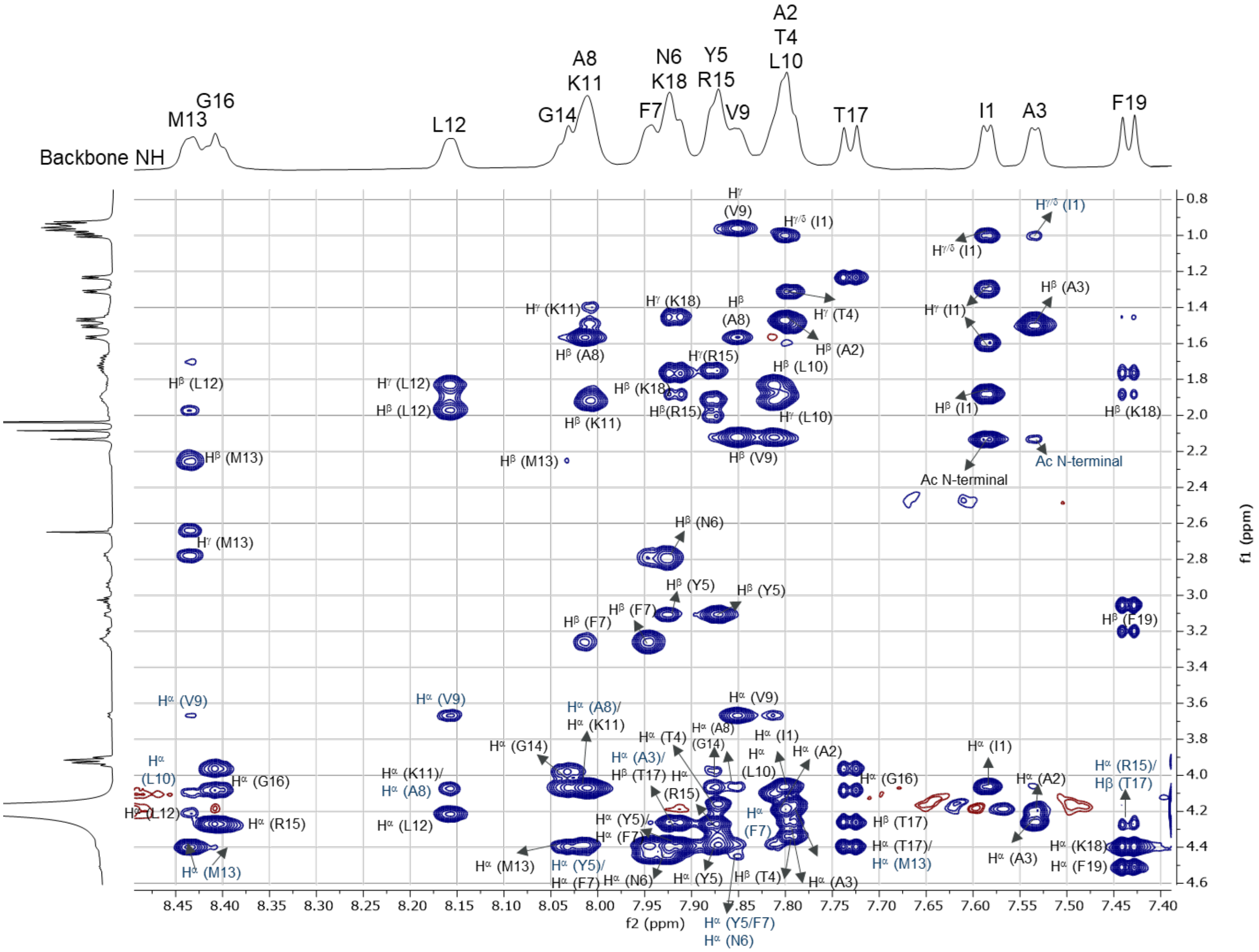

Figure S18) ROESY spectrum (NH/aliphatic ^{1}H region, normalised by largest peak, value: 100) of the PLP peptide (1 mM) in 80%-TFE/20%-H_2O (v/v) recorded at 600 MHz at 298 K. In the expansion, ROEs due to sequential and medium-range distances, including dαN (i, i+3) and dαN (i, i+4), are present through all the sequence (blue).

Residue	NH	H^{α} (C^{α})	H^{β} (C^{β})	Others
**I1**	7.59	4.07* (60.2)	1.89 (35.9)	1.60-1.30 (25.1) $C^{\gamma}H_2$<br>1.02** (14.0) $C^{\gamma}H_3$<br>0.98** (9.4) $C^{\delta}H_3$
**A2**	7.80	4.21*(51.34)	1.48(14.9)	--
**A3**	7.54	4.26*(51.4)	1.51(15.1)	--
**T4**	7.80	4.16(61.8)	4.34(66.8)	1.32(18.2) $C^{\gamma}H_3$
**Y5**	7.88	4.40*(57.7***)	3.11(35.3)	7.13-6.82(130.0-115.3) H_{Ar}
**N6**	7.93	4.47(52.9)	2.83-2.77(35.4)	7.15-6.35 NH_2
**F7**	7.95	4.40*(57.7***)	3.29-3.24(36.0)	7.34/7.26** (126.6/129.3***) H_{Ar}
**A8**	8.02	4.08*(52.7)	1.58(14.7)	--
**V9**	7.86	3.68(63.7)	2.12(29.0)	0.97**(18.0) $C^{\gamma}H_3$
**L10**	7.82	4.12(55.4)	1.92-1.57(39.0)	1.83**(weak)(24.2) $C^{\gamma}H$<br>1.00-0.91*(19.0/19.9/21.4) $C^{\delta}H_3$
**K11**	8.01	4.08*(56.0)	1.92-1.88(29.0)	1.43(21.6) $C^{\gamma}H_2$<br>1.68(26.0***) $C^{\delta}H_2$<br>2.95(39.4) $C^{\varepsilon}H_2$
**L12**	8.16	4.21*(54.9)	1.97-1.70(39.3)	1.83**(24.2***) $C^{\gamma}H$<br>0.93**(19.0/19.9/21.4) $C^{\delta}H_3$
**M13**	8.44	4.41*(53.4)	2.29-2.20(29.6)	2.79-2.65(29.6) $C^{\gamma}H_2$<br>2.09(13.3) $C^{\varepsilon}H_3$
**G14**	8.04	4.05-4.00(43.0***)	--	--
**R15**	7.88	4.28*(54.1)	2.01-1.92(27.3)	3.25(40.4) $C^{\delta}H_2$<br>1.76(24.1) $C^{\gamma}H_2$<br>7.16 $N^{\varepsilon}H$
**G16**	8.44	4.10-3.96(43.0***)	--	--
**T17**	7.73	4.40*(59.0)	4.26*(67.3)	1.24(18.2) $C^{\gamma}H_3$
**K18**	7.92	4.40*(54.3)	1.88-1.77(30.5)	1.47-1.43(21.8***) $C^{\gamma}H_2$<br>1.74(25.9***) $C^{\delta}H_2$<br>3.04(39.5) $C^{\varepsilon}H_2$
**F19**	7.44	4.52(56.1)	3.20-3.07(37.3)	7.34/7.26**(126.6/129.3***) H_{Ar}

Table S6) ^{1}H and ^{13}C resonance assignments (ppm) of the PLP peptide (1 mM) in 80%-TFE/20%-H_2O (v/v) recorded at 600 MHz at 298 K.

*, ** overlapping of the ^{1}H δ, ^{13}C δ were assigned, as most probable, on the base of ”random coil” values[1]

*** ^{13}C δ overlapping

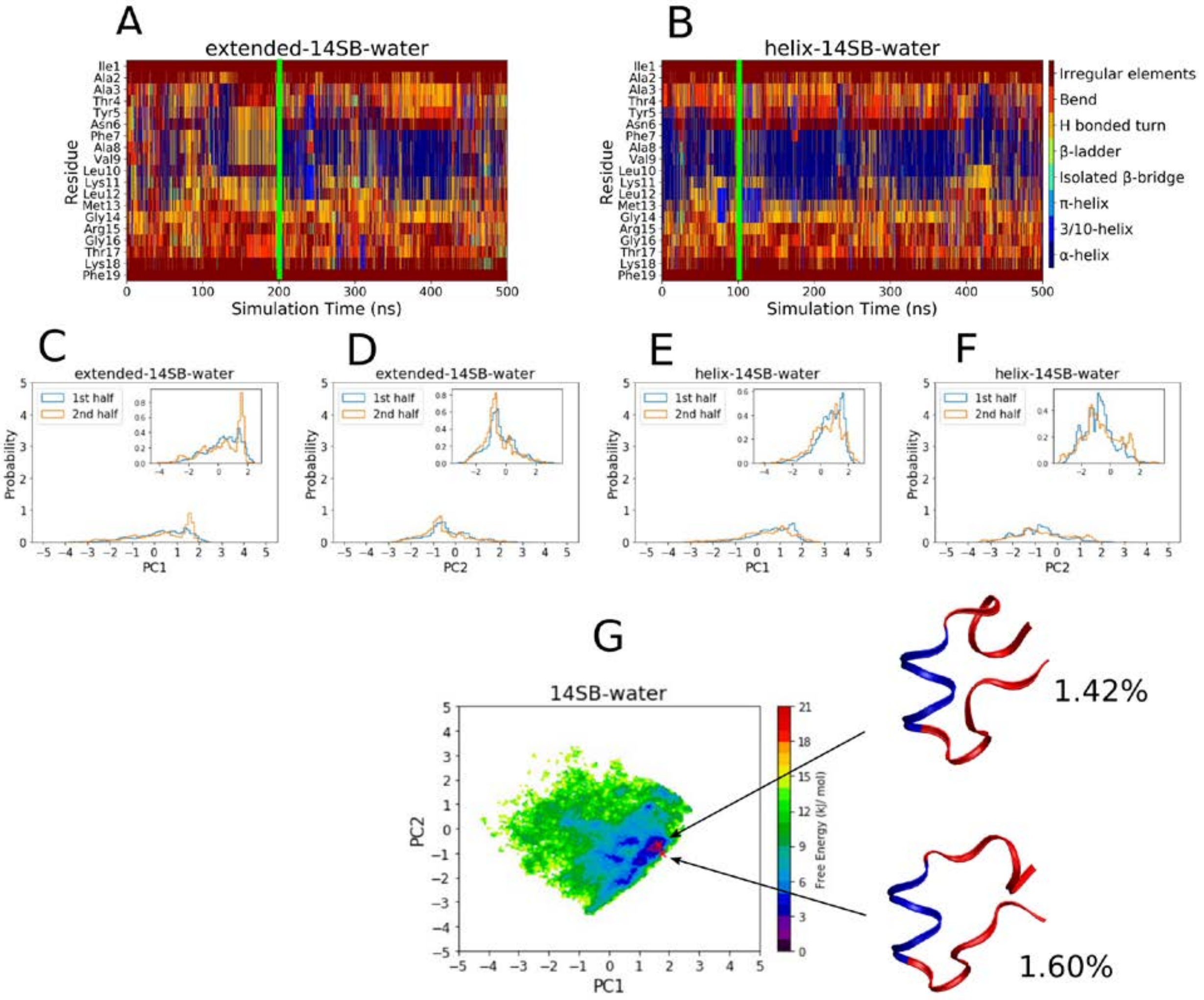

Figure S19) Results from the PLP peptide ff14SB water simulations. **A-B)** DSSP analysis of the simulations starting from **A)** an extended conformation and **B)** a helical conformation. The green bar on each plot indicates the time from which equilibrium analysis was performed. **C-F)** Histograms of the projections of the simulation coordinates onto PC1 (**C** and **E**) and PC2 (**D** and **F**) built from the first and second halves of the equilibrated part of the extended (**C** and **D**) and helical (**E** and **F**) trajectories. The y axes maxima are set to 5 to allow comparison between all of the PLP peptide histograms (**Figures 1-2 C-F** and **Figures S19-S28 C-F**), however zoomed in inserts are provided when the largest peak is less than 2. **G)** The FES with respect to PC1 and PC2 built from the combined equilibrated parts of the two trajectories. The energy minimum is set to 0 and the colourbar range is fixed at 0-21 kJ mol^{-1} to allow comparison between all of the PLP peptide FESs (**Figure 2 G** and **Figures S19-S28 G**). The DASH clusters are overlayed on the surface as red crosses, and those that occupy a similar PC space are grouped into macrostates with the corresponding structural representatives shown as a superposition of the cluster centroids. The structures are shown as ribbons with random coil, turn and bend residues shown in red, α-helical residues shown in dark blue, 3_{10}-helical residues shown in light blue, β-bridge residues shown in cyan and β-ladder residues shown in lime. The percentage of trajectory frames occupied by each macrostate is also shown.

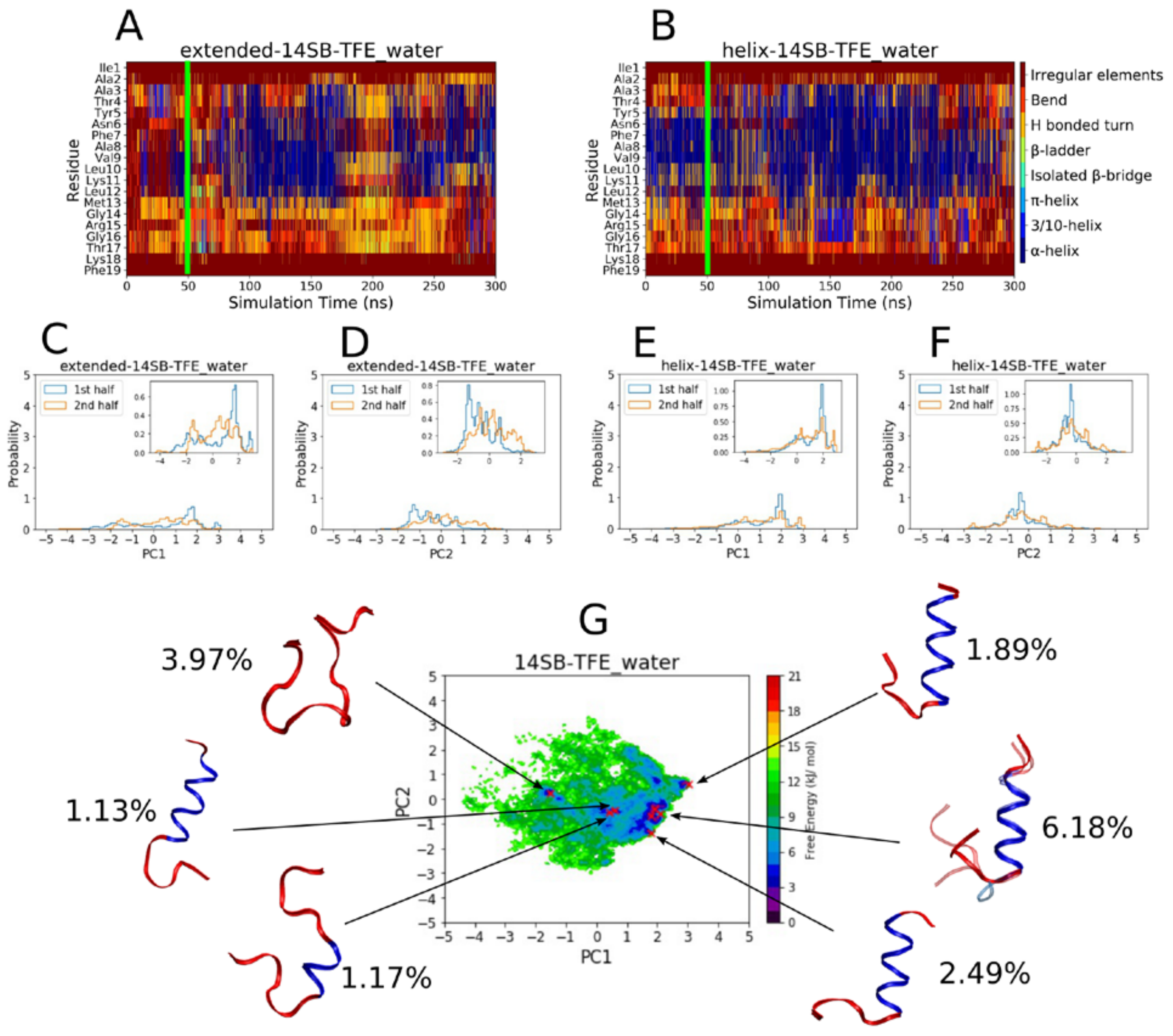

Figure S20) Results from the PLP peptide ff14SB TFE:water simulations. **A-B)** DSSP analysis of the simulations starting from **A)** an extended conformation and **B)** a helical conformation. The green bar on each plot indicates the time from which equilibrium analysis was performed. **C-F)** Histograms of the projections of the simulation coordinates onto PC1 (**C** and **E**) and PC2 (**D** and **F**) built from the first and second halves of the equilibrated part of the extended (**C** and **D**) and helical (**E** and **F**) trajectories. The y axes maxima are set to 5 to allow comparison between all of the PLP peptide histograms (**Figures 1-2 C-F** and **Figures S19-S28 C-F**), however zoomed in inserts are provided when the largest peak is less than 2. **G)** The FES with respect to PC1 and PC2 built from the combined equilibrated parts of the two trajectories. The energy minimum is set to 0 and the colourbar range is fixed at 0-21 kJ mol^{-1} to allow comparison between all of the PLP peptide FESs (**Figure 2 G** and **Figures S19-S28 G**). The DASH clusters are overlayed on the surface as red crosses, and those that occupy a similar PC space are grouped into macrostates with the corresponding structural representatives shown as a superposition of the cluster centroids. The structures are shown as ribbons with random coil, turn and bend residues shown in red, α-helical residues shown in dark blue, 3_{10}-helical residues shown in light blue, β-bridge residues shown in cyan and β-ladder residues shown in lime. The percentage of trajectory frames occupied by each macrostate is also shown.

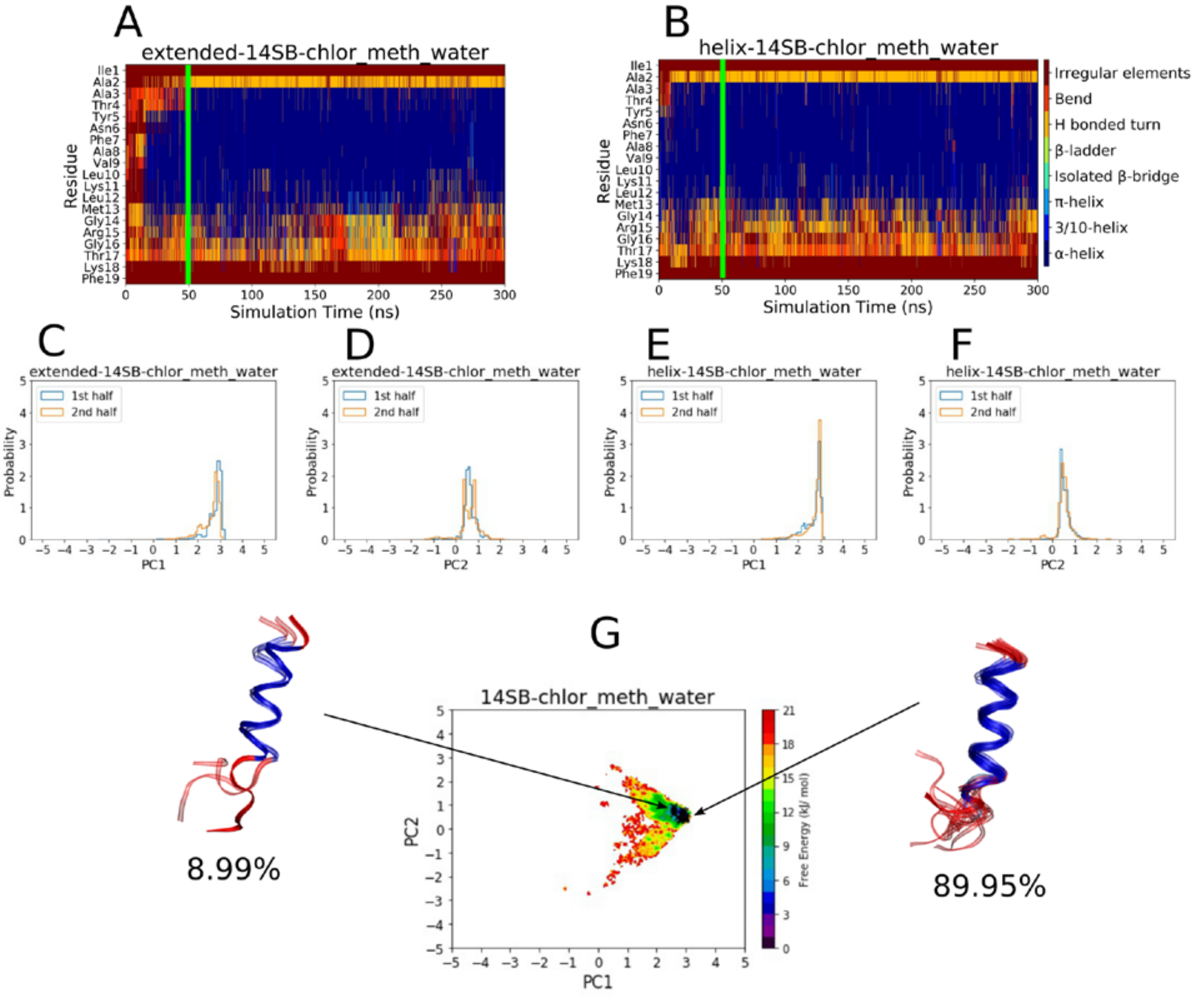

Figure S21) Results from the PLP peptide ff14SB chlorodorm:methanol:water simulations. **A-B)** DSSP analysis of the simulations starting from **A)** an extended conformation and **B)** a helical conformation. The green bar on each plot indicates the time from which equilibrium analysis was performed. **C-F)** Histograms of the projections of the simulation coordinates onto PC1 (**C** and **E**) and PC2 (**D** and **F**) built from the first and second halves of the equilibrated part of the extended (**C** and **D**) and helical (**E** and **F**) trajectories. The y axes maxima are set to 5 to allow comparison between all of the PLP peptide histograms (**Figures 1-2 C-F** and **Figures S19-S28 C-F**), however zoomed in inserts are provided when the largest peak is less than 2. **G)** The FES with respect to PC1 and PC2 built from the combined equilibrated parts of the two trajectories. The energy minimum is set to 0 and the colourbar range is fixed at 0-21 kJ mol^{-1} to allow comparison between all of the PLP peptide FESs (**Figure 2 G** and **Figures S19-S28 G**). The DASH clusters are overlayed on the surface as black crosses, and those that occupy a similar PC space are grouped into macrostates with the corresponding structural representatives shown as a superposition of the cluster centroids. The structures are shown as ribbons with random coil, turn and bend residues shown in red, α-helical residues shown in dark blue, 3_{10}-helical residues shown in light blue, β-bridge residues shown in cyan and β-ladder residues shown in lime. The percentage of trajectory frames occupied by each macrostate is also shown.

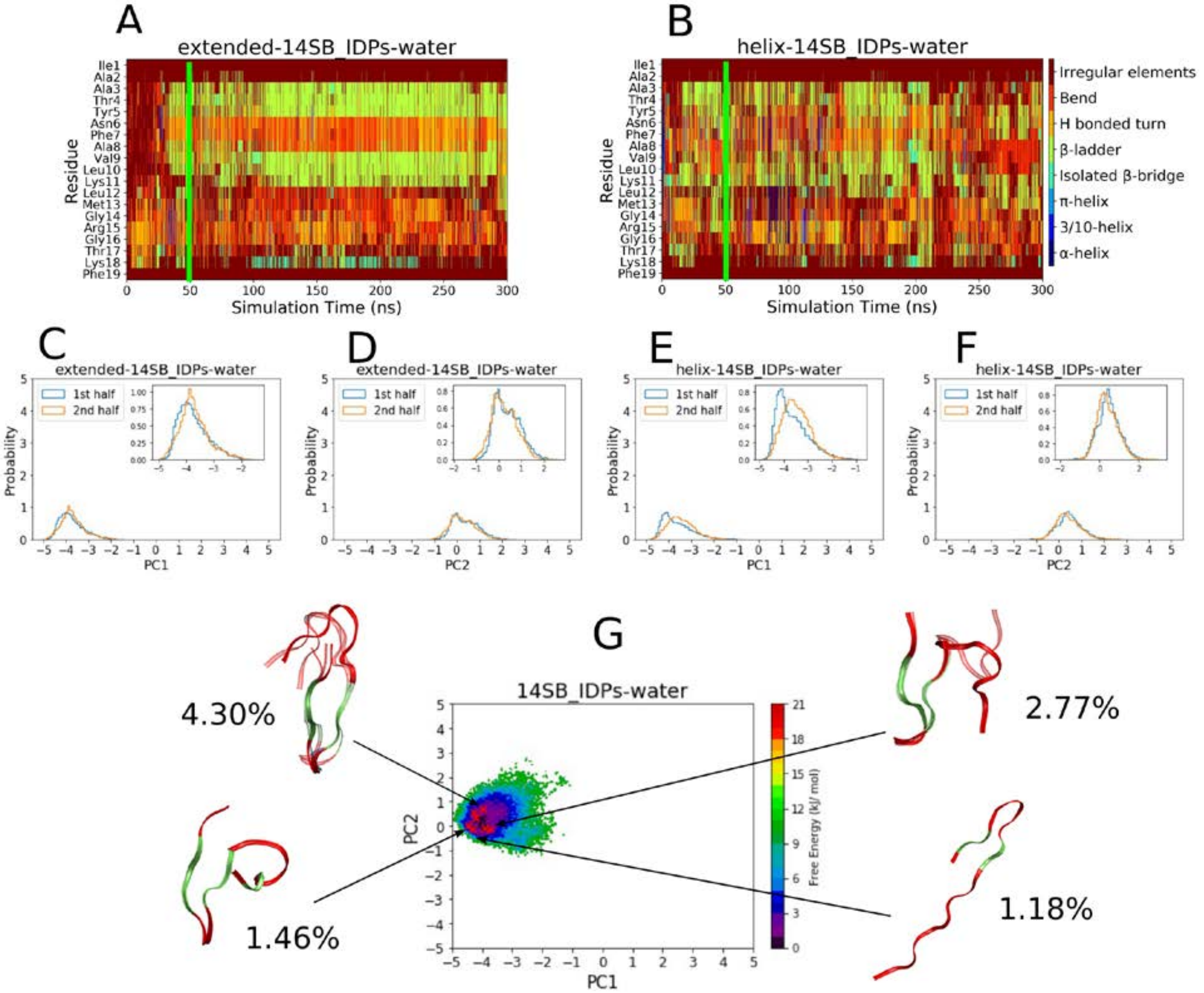

Figure S22) Results from the PLP peptide ff14IDPSFF water simulations. **A-B)** DSSP analysis of the simulations starting from **A)** an extended conformation and **B)** a helical conformation. The green bar on each plot indicates the time from which equilibrium analysis was performed. **C-F)** Histograms of the projections of the simulation coordinates onto PC1 (**C** and **E**) and PC2 (**D** and **F**) built from the first and second halves of the equilibrated part of the extended (**C** and **D**) and helical (**E** and **F**) trajectories. The y axes maxima are set to 5 to allow comparison between all of the PLP peptide histograms (**Figures 1-2 C-F** and **Figures S19-S28 C-F**), however zoomed in inserts are provided when the largest peak is less than 2. **G)** The FES with respect to PC1 and PC2 built from the combined equilibrated parts of the two trajectories. The energy minimum is set to 0 and the colourbar range is fixed at 0-21 kJ mol^{-1} to allow comparison between all of the PLP peptide FESs (**Figure 2 G** and **Figures S19-S28 G**). The DASH clusters are overlayed on the surface as red crosses, and those that occupy a similar PC space are grouped into macrostates with the corresponding structural representatives shown as a superposition of the cluster centroids. The structures are shown as ribbons with random coil, turn and bend residues shown in red, α-helical residues shown in dark blue, 3_{10}-helical residues shown in light blue, β-bridge residues shown in cyan and β-ladder residues shown in lime. The percentage of trajectory frames occupied by each macrostate is also shown.

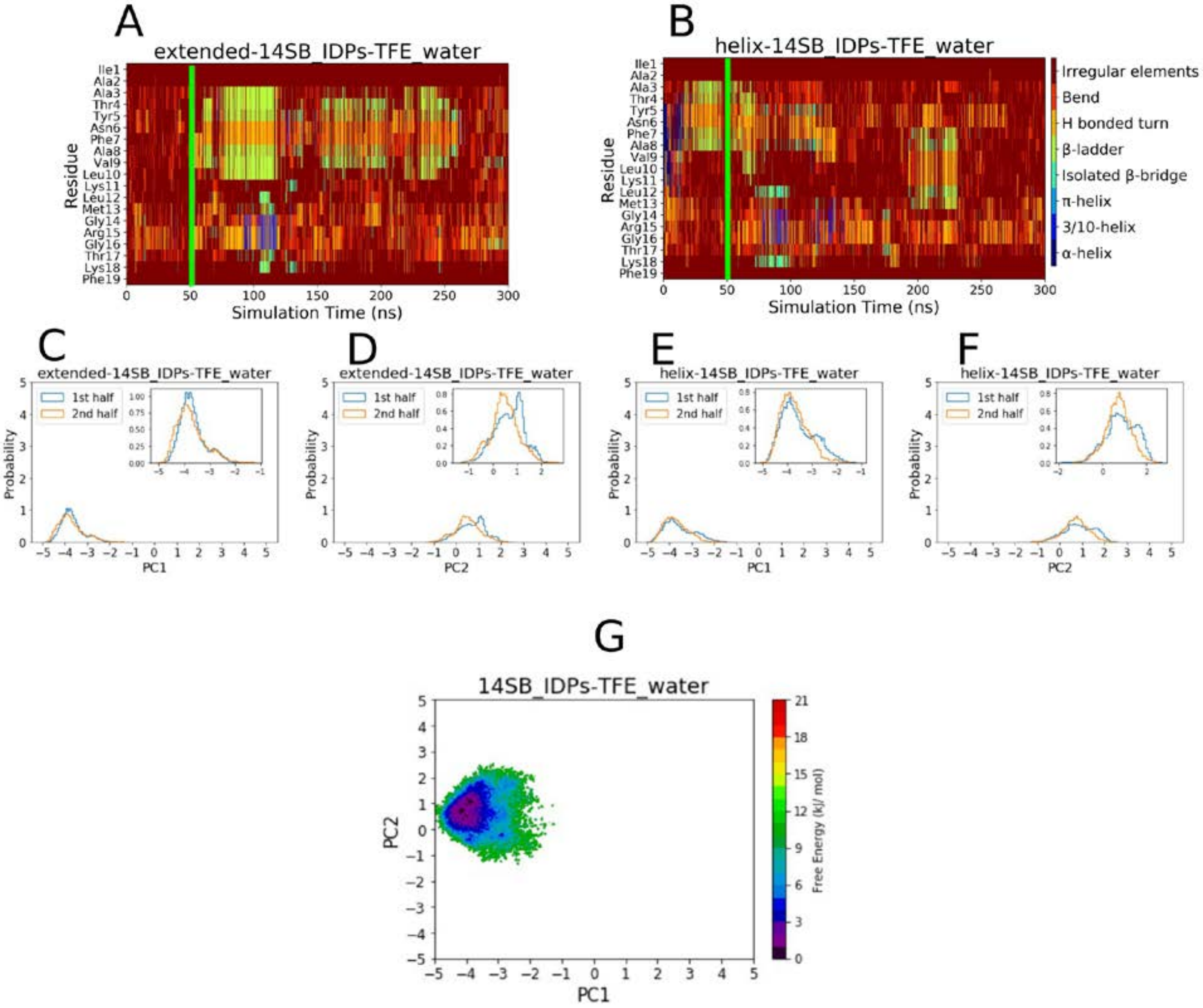

Figure S23) Results from the PLP peptide ff14IDPSFF TFE:water simulations. **A-B)** DSSP analysis of the simulations starting from **A)** an extended conformation and **B)** a helical conformation. The green bar on each plot indicates the time from which equilibrium analysis was performed. **C-F)** Histograms of the projections of the simulation coordinates onto PC1 (**C** and **E**) and PC2 (**D** and **F**) built from the first and second halves of the equilibrated part of the extended (**C** and **D**) and helical (**E** and **F**) trajectories. The y axes maxima are set to 5 to allow comparison between all of the PLP peptide histograms (**Figures 1-2 C-F** and **Figures S19-S28 C-F**), however zoomed in inserts are provided when the largest peak is less than 2. **G)** The FES with respect to PC1 and PC2 built from the combined equilibrated parts of the two trajectories. The energy minimum is set to 0 and the colourbar range is fixed at 0-21 kJ mol^{-1} to allow comparison between all of the PLP peptide FESs (**Figure 2 G** and **Figures S19-S28 G**). No DASH clusters were identified.

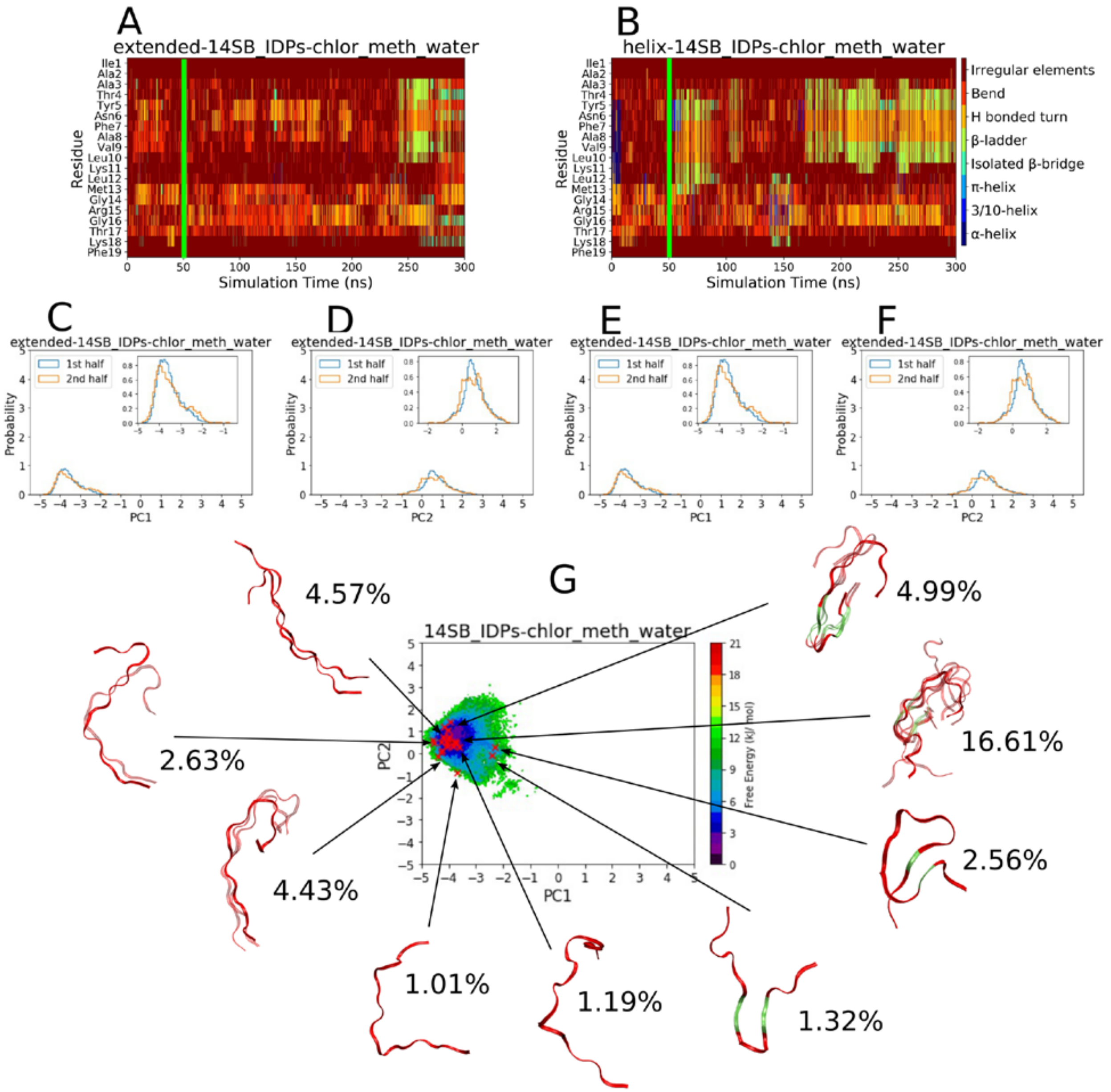

Figure S24) Results from the PLP peptide ff14IDPSFF chloroform:methanol:water simulations. **A-B)** DSSP analysis of the simulations starting from **A)** an extended conformation and **B)** a helical conformation. The green bar on each plot indicates the time from which equilibrium analysis was performed. **C-F)** Histograms of the projections of the simulation coordinates onto PC1 (**C** and **E**) and PC2 (**D** and **F**) built from the first and second halves of the equilibrated part of the extended (**C** and **D**) and helical (**E** and **F**) trajectories. The y axes maxima are set to 5 to allow comparison between all of the PLP peptide histograms (**Figures 1-2 C-F** and **Figures S19-S28 C-F**), however zoomed in inserts are provided when the largest peak is less than 2. **G)** The FES with respect to PC1 and PC2 built from the combined equilibrated parts of the two trajectories. The energy minimum is set to 0 and the colourbar range is fixed at 0-21 kJ mol^{-1} to allow comparison between all of the PLP peptide FESs (**Figure 2 G** and **Figures S19-S28 G**). The DASH clusters are overlayed on the surface as red crosses, and those that occupy a similar PC space are grouped into macrostates with the corresponding structural representatives shown as a superposition of the cluster centroids. The structures are

shown as ribbons with random coil, turn and bend residues shown in red, α-helical residues shown in dark blue, 3_{10}-helical residues shown in light blue, β-bridge residues shown in cyan and β-ladder residues shown in lime. The percentage of trajectory frames occupied by each macrostate is also shown.

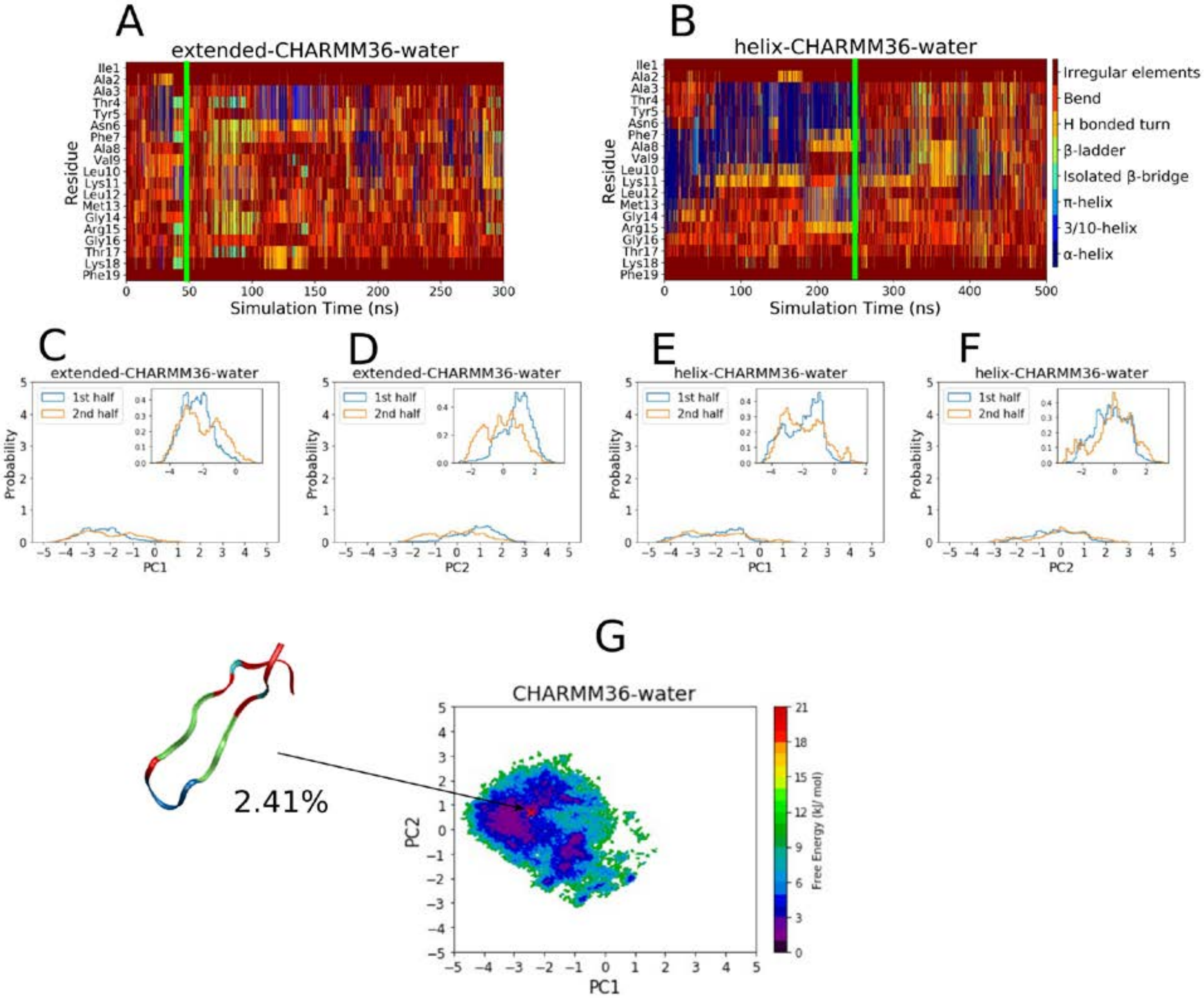

Figure S25) Results from the PLP peptide CHARMM36 water simulations. **A-B)** DSSP analysis of the simulations starting from **A)** an extended conformation and **B)** a helical conformation. The green bar on each plot indicates the time from which equilibrium analysis was performed. **C-F)** Histograms of the projections of the simulation coordinates onto PC1 (**C** and **E**) and PC2 (**D** and **F**) built from the first and second halves of the equilibrated part of the extended (**C** and **D**) and helical (**E** and **F**) trajectories. The y axes maxima are set to 5 to allow comparison between all of the PLP peptide histograms (**Figures 1-2 C-F** and **Figures S19-S28 C-F**), however zoomed in inserts are provided when the largest peak is less than 2. **G)** The FES with respect to PC1 and PC2 built from the combined equilibrated parts of the two trajectories. The energy minimum is set to 0 and the colourbar range is fixed at 0-21 kJ mol^{-1} to allow comparison between all of the PLP peptide FESs (**Figure 2 G** and **Figures S19-28 G**). The DASH clusters are overlayed on the surface as red crosses, and those that occupy a similar PC space are grouped into macrostates with the corresponding structural representatives shown as a superposition of the cluster centroids. The structures are shown as ribbons with

random coil, turn and bend residues shown in red, α-helical residues shown in dark blue, 3_{10}-helical residues shown in light blue, β-bridge residues shown in cyan and β-ladder residues shown in lime. The percentage of trajectory frames occupied by each macrostate is also shown.

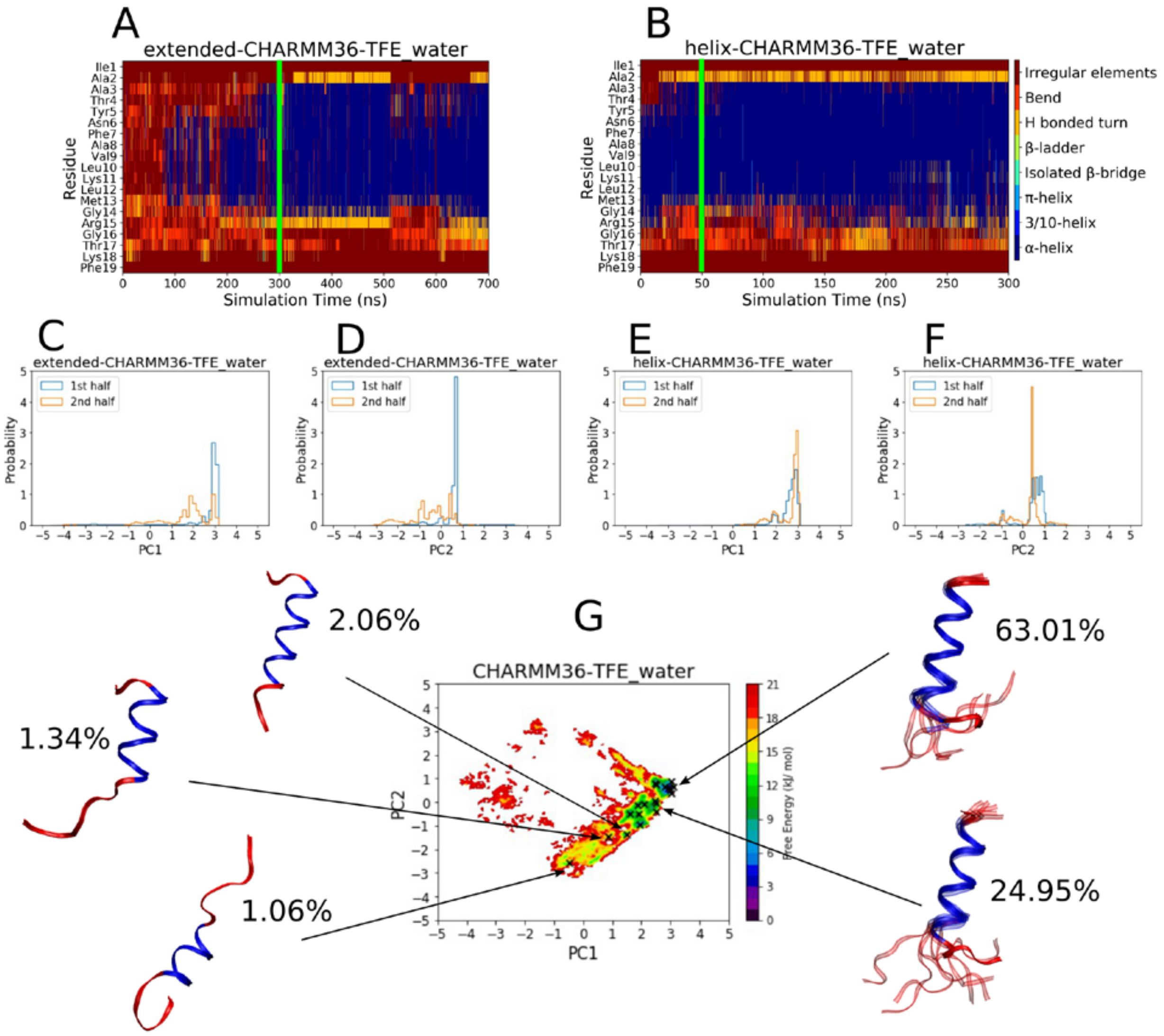

Figure S26) Results from the PLP peptide CHARMM36 TFE:water simulations. **A-B)** DSSP analysis of the simulations starting from **A)** an extended conformation and **B)** a helical conformation. The green bar on each plot indicates the time from which equilibrium analysis was performed. **C-F)** Histograms of the projections of the simulation coordinates onto PC1 (**C** and **E**) and PC2 (**D** and **F**) built from the first and second halves of the equilibrated part of the extended (**C** and **D**) and helical (**E** and **F**) trajectories. The y axes maxima are set to 5 to allow comparison between all of the PLP peptide histograms (**Figures 1-2 C-F** and **Figures S19-S28 C-F**), however zoomed in inserts are provided when the largest peak is less than 2. **G)** The FES with respect to PC1 and PC2 built from the combined equilibrated parts of the two

trajectories. The energy minimum is set to 0 and the colourbar range is fixed at 0-21 kJ mol^{-1} to allow comparison between all of the PLP peptide FESs (**Figure 2 G** and **Figures S19-S28 G**). The DASH clusters are overlayed on the surface as black crosses, and those that occupy a similar PC space are grouped into macrostates with the corresponding structural representatives shown as a superposition of the cluster centroids. The structures are shown as ribbons with random coil, turn and bend residues shown in red, α-helical residues shown in dark blue, 3_{10}-helical residues shown in light blue, β-bridge residues shown in cyan and β-ladder residues shown in lime. The percentage of trajectory frames occupied by each macrostate is also shown.

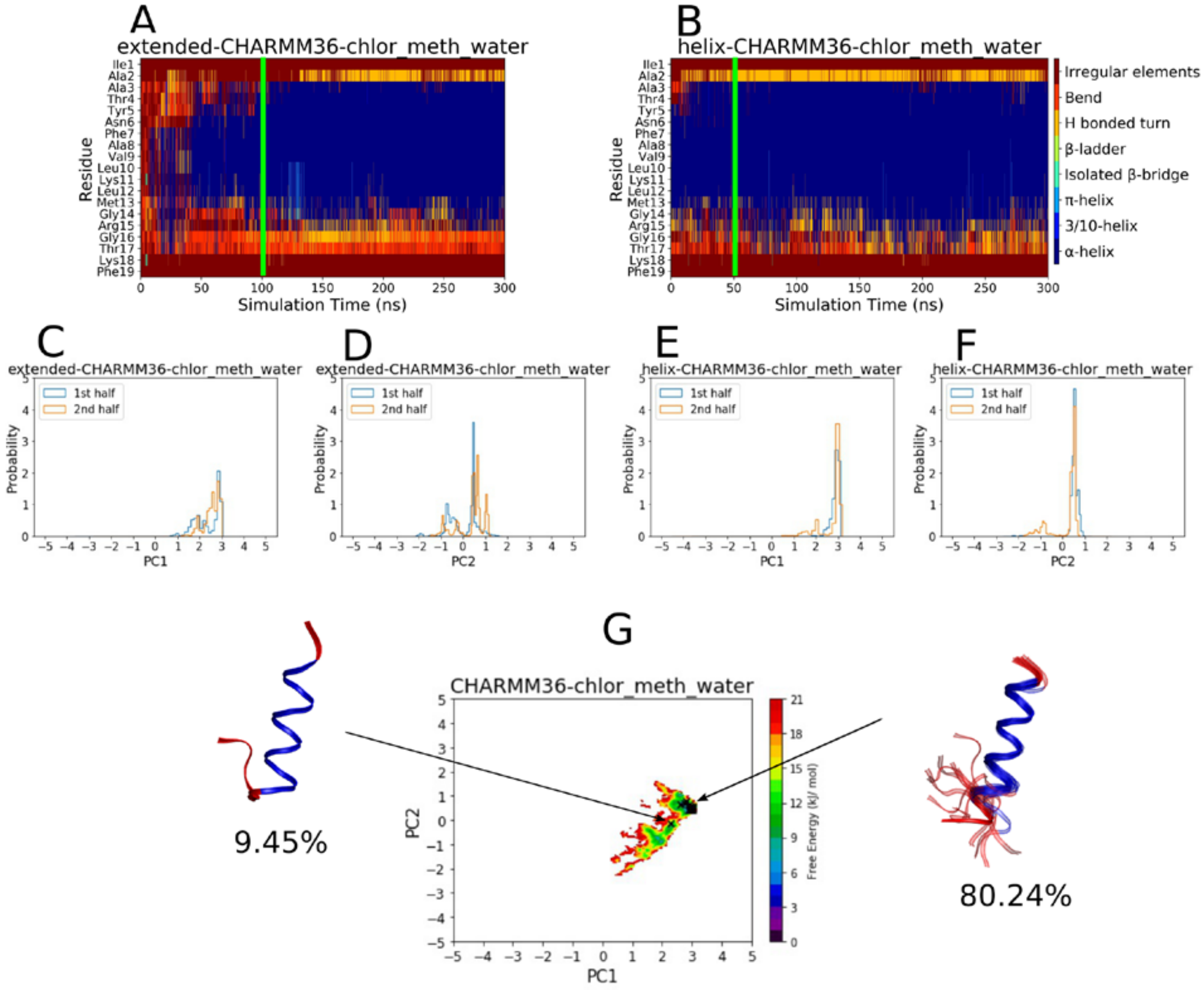

Figure S27) Results from the PLP peptide CHARMM36 chloroform:methanol:water simulations. **A-B)** DSSP analysis of the simulations starting from **A)** an extended conformation and **B)** a helical conformation. The green bar on each plot indicates the time from which equilibrium analysis was performed. **C-F)** Histograms of the projections of the simulation coordinates onto PC1 (**C** and **E**) and PC2 (**D** and **F**) built from the first and second halves of the equilibrated part of the

extended (**C** and **D**) and helical (**E** and **F**) trajectories. The y axes maxima are set to 5 to allow comparison between all of the PLP peptide histograms (**Figures 1-2 C-F** and **Figures S19-S28 C-F**), however zoomed in inserts are provided when the largest peak is less than 2. **G)** The FES with respect to PC1 and PC2 built from the combined equilibrated parts of the two trajectories. The energy minimum is set to 0 and the colourbar range is fixed at 0-21 kJ mol^{-1} to allow comparison between all of the PLP peptide FESs (**Figure 2 G** and **Figures S19-S28 G**). The DASH clusters are overlayed on the surface as black crosses, and those that occupy a similar PC space are grouped into macrostates with the corresponding structural representatives shown as a superposition of the cluster centroids. The structures are shown as ribbons with random coil, turn and bend residues shown in red, α-helical residues shown in dark blue, 3_{10}-helical residues shown in light blue, β-bridge residues shown in cyan and β-ladder residues shown in lime. The percentage of trajectory frames occupied by each macrostate is also shown.

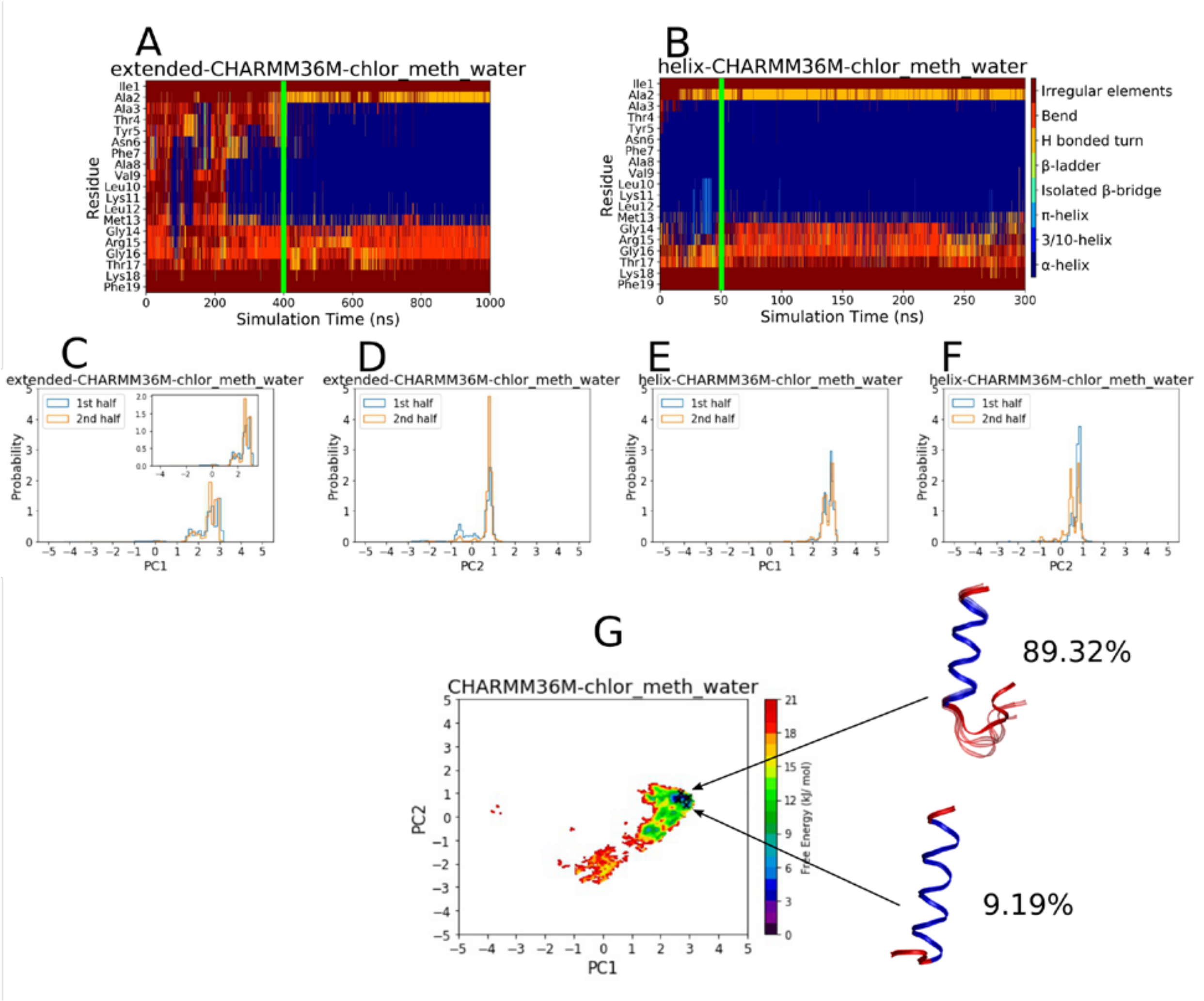

Figure S28) Results from the PLP peptide CHARMM36m chloroform:methanol:water simulations. **A-B)** DSSP analysis of the simulations starting from **A)** an extended conformation and **B)** a helical conformation. The green bar on each plot indicates the time from which equilibrium analysis was performed. **C-F)** Histograms of the projections of the simulation coordinates onto PC1 (**C** and **E**) and PC2 (**D** and **F**) built from the first and second halves of the equilibrated part of the extended (**C** and **D**) and helical (**E** and **F**) trajectories. The y axes maxima are set to 5 to allow comparison between all of the PLP peptide histograms (**Figures 1-2 C-F** and **Figures S19-S28 C-F**), however zoomed in inserts are provided

when the largest peak is less than 2. **G)** The FES with respect to PC1 and PC2 built from the combined equilibrated parts of the two trajectories. The energy minimum is set to 0 and the colourbar range is fixed at 0-21 kJ mol^{-1} to allow comparison between all of the PLP peptide FESs (**Figure 2 G** and **Figures S19-S28 G**). The DASH clusters are overlayed on the surface as black crosses, and those that occupy a similar PC space are grouped into macrostates with the corresponding structural representatives shown as a superposition of the cluster centroids. The structures are shown as ribbons with random coil, turn and bend residues shown in red, α-helical residues shown in dark blue, 3_{10}-helical residues shown in light blue, β-bridge residues shown in cyan and β-ladder residues shown in lime. The percentage of trajectory frames occupied by each macrostate is also shown.

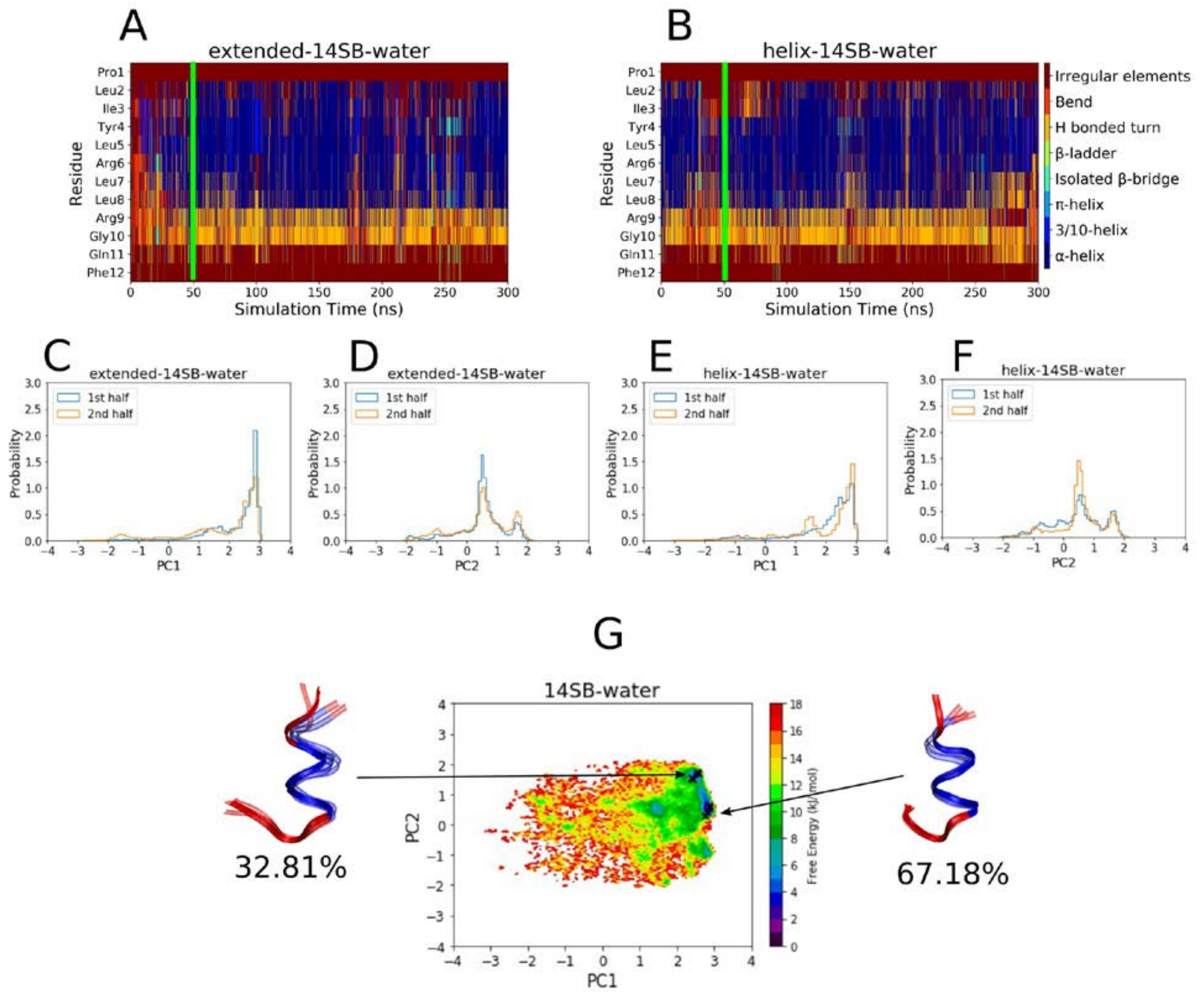

Figure S29) Results from the TP2 ff14SB water simulations. **A-B)** DSSP analysis of the simulations starting from **A)** an extended conformation and **B)** a helical conformation. The green bar on each plot indicates the time from which equilibrium analysis was performed. **C-F)** Histograms of the projections of the simulation coordinates onto PC1 (**C** and **E**) and PC2 (**D** and **F**) built from the first and second halves of the equilibrated part of the extended (**C** and **D**) and helical (**E** and **F**) trajectories. The y axes maxima are set to 3 to allow comparison between all of the TP2 and ONEG histograms (**Figures S29-S40 C-F**), however zoomed in inserts are provided when the largest peak is less than 1. **G)**

The FES with respect to PC1 and PC2 built from the combined equilibrated parts of the two trajectories. The energy minimum is set to 0 and the colourbar range is fixed at 0-18 kJ mol^{-1} to allow comparison between all of the TP2 and ONEG FESs (**Figures S29-S40 G**). The DASH clusters are overlayed on the surface as black crosses, and those that occupy a similar PC space are grouped into macrostates with the corresponding structural representatives shown as a superposition of the cluster centroids. The structures are shown as ribbons with random coil, turn and bend residues shown in red, α-helical residues shown in dark blue, 3_{10}-helical residues shown in light blue, β-bridge residues shown in cyan and β-ladder residues shown in lime. The percentage of trajectory frames occupied by each macrostate is also shown.

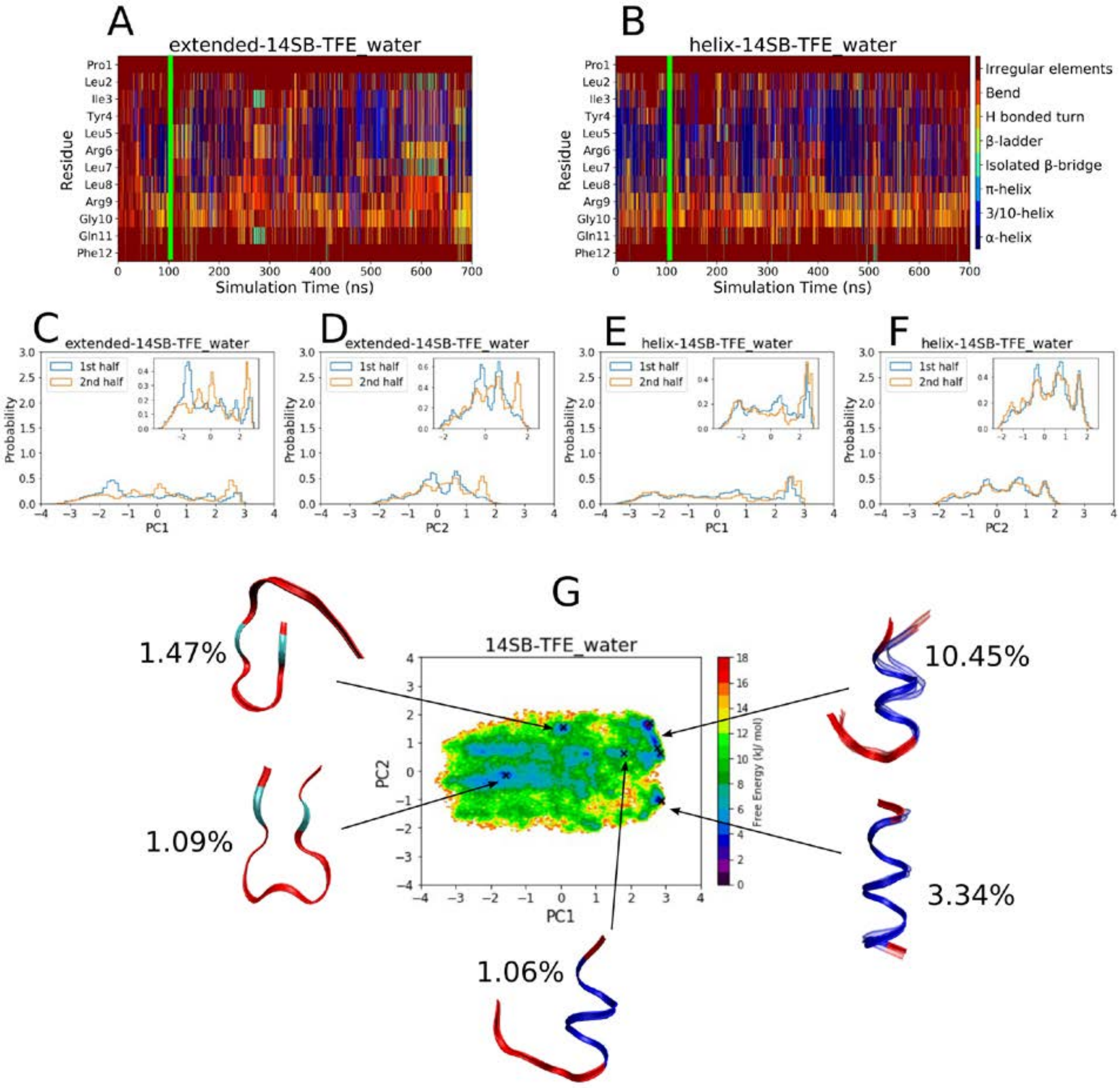

Figure S30) Results from the TP2 ff14SB TFE:water simulations. **A-B)** DSSP analysis of the simulations starting from **A)** an extended conformation and **B)** a helical conformation. The green bar on each plot indicates the time from which equilibrium analysis was performed. **C-F)** Histograms of the projections of the simulation coordinates onto PC1 (**C** and **E**) and PC2 (**D** and **F**) built from the first and second halves of the equilibrated part of the extended (**C** and **D**) and helical (**E** and **F**) trajectories. The y axes maxima are set to 3 to allow comparison between all of the TP2 and ONEG histograms (**Figures S29-S40 C-F**), however zoomed in inserts are provided when the largest peak is less than 1. **G)** The FES with respect to PC1 and PC2 built from the combined equilibrated parts of the two trajectories. The energy minimum is set to 0 and the colourbar range is fixed at 0-18 kJ mol^{-1} to allow comparison between all of the TP2 and ONEG FESs (**Figures S29-S40 G**). The DASH clusters are overlayed on the surface as black crosses, and those that occupy a similar PC space are grouped into macrostates with the corresponding structural representatives shown as a superposition of the cluster centroids. The structures are shown as ribbons with random coil, turn and bend residues shown in red, α-helical residues shown in dark blue, 3_{10}-helical residues shown in light blue, β-bridge residues shown in cyan and β-ladder residues shown in lime. The percentage of trajectory frames occupied by each macrostate is also shown.

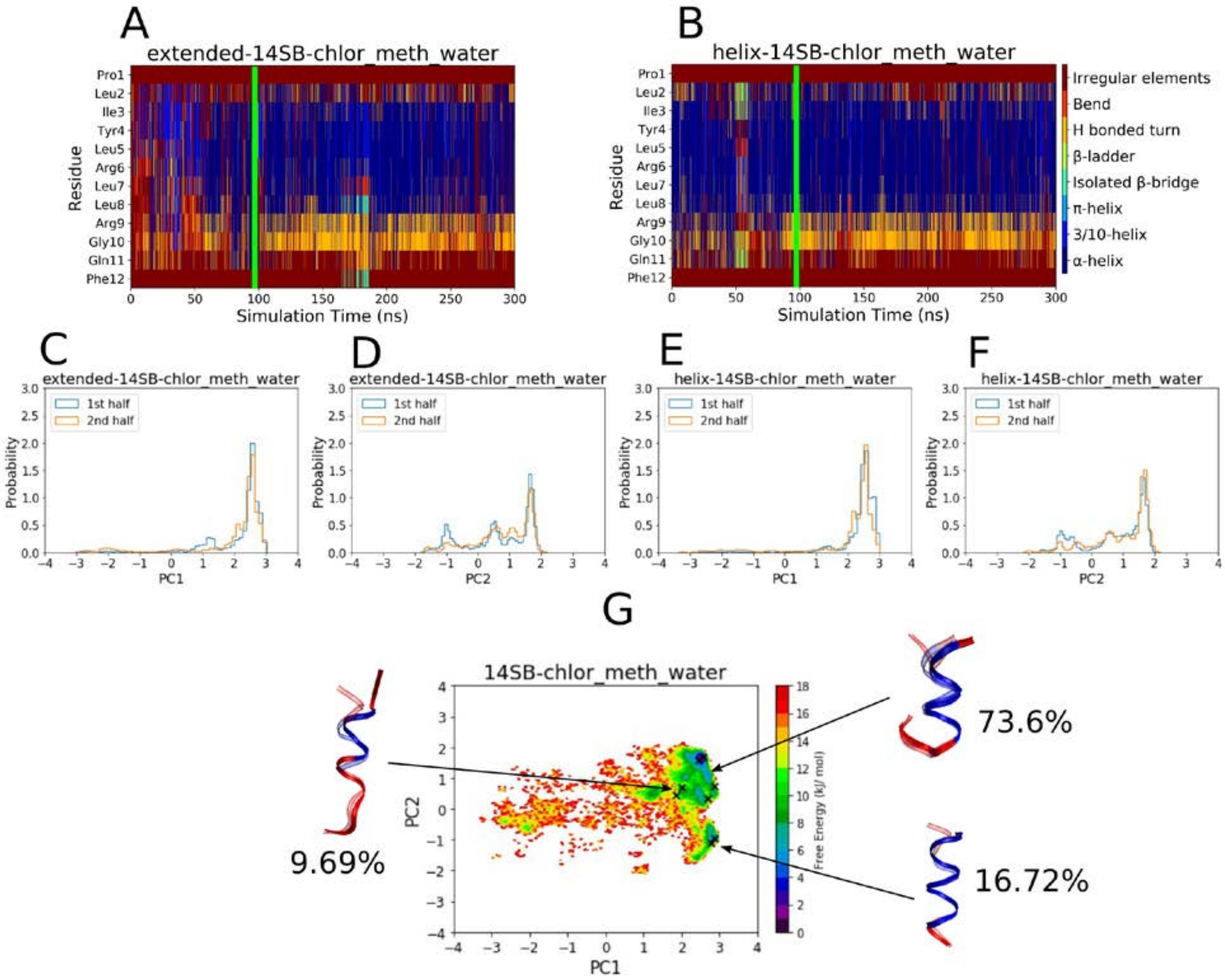

Figure S31) Results from the TP2 ff14SB chloroform:methanol:water simulations. **A-B)** DSSP analysis of the simulations starting from **A)** an extended conformation and **B)** a helical conformation. The green bar on each plot indicates the time from which equilibrium analysis was performed. **C-F)** Histograms of the projections of the simulation coordinates onto PC1 (**C** and **E**) and PC2 (**D** and **F**) built from the first and second halves of the equilibrated part of the extended (**C** and **D**) and helical (**E** and **F**) trajectories. The y axes maxima are set to 3 to allow comparison between all of the TP2 and ONEG histograms (**Figures S29-S40 C-F**), however zoomed in inserts are provided when the largest peak is less than 1. **G)** The FES with respect to PC1 and PC2 built from the combined equilibrated parts of the two trajectories. The energy minimum is set to 0 and the colourbar range is fixed at 0-18 kJ mol^{-1} to allow comparison between all of the TP2 and ONEG FESs (**Figures S29-S40 G**). The DASH clusters are overlayed on the surface as black crosses, and those that occupy a similar PC space are grouped into macrostates with the corresponding structural representatives shown as a superposition of the cluster centroids. The structures are shown as ribbons with random coil, turn and bend residues shown in red, α-helical residues shown in dark blue, 3_{10}-helical residues shown in light blue, β-bridge residues shown in cyan and β-ladder residues shown in lime. The percentage of trajectory frames occupied by each macrostate is also shown.

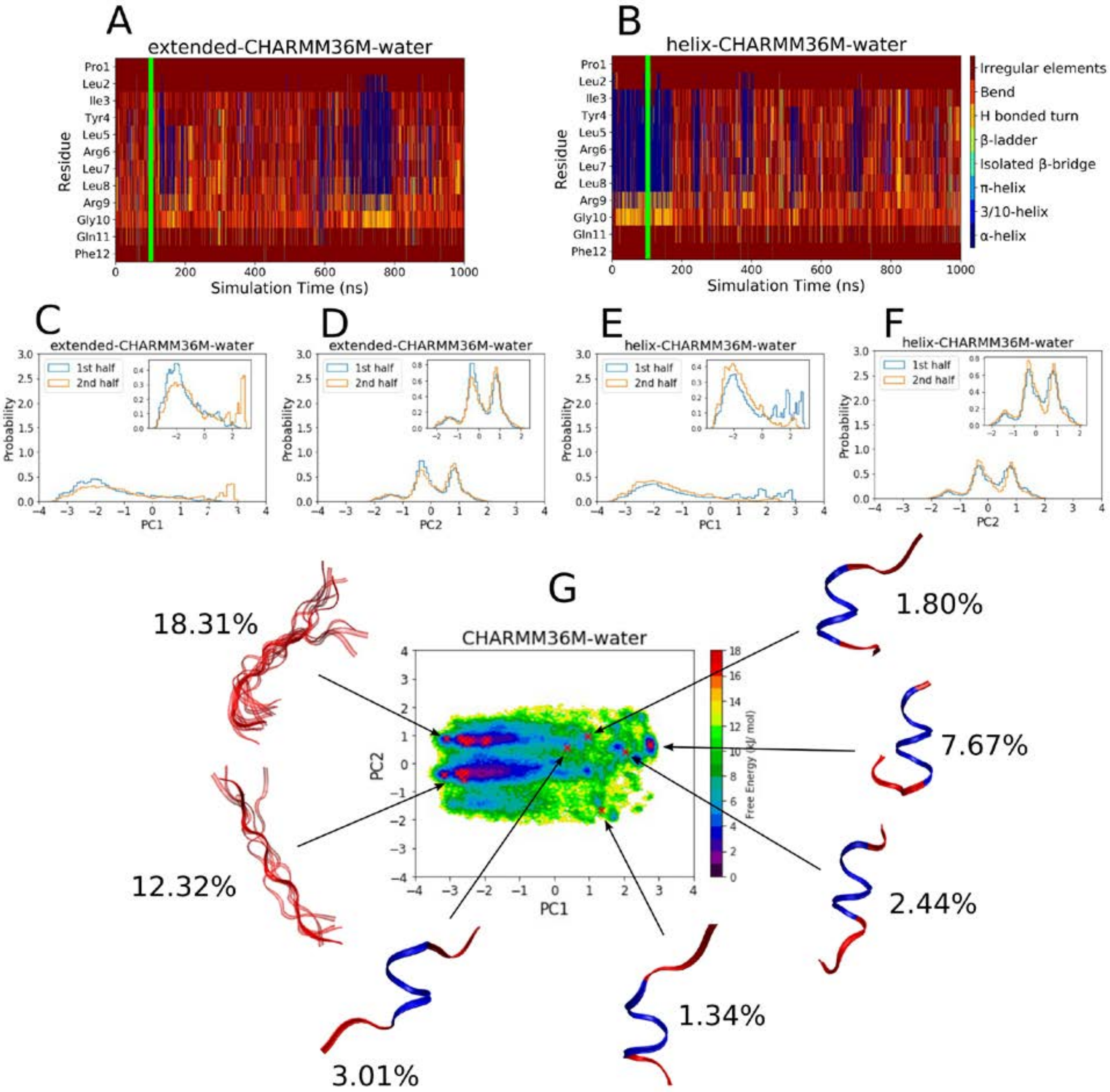

Figure S32) Results from the TP2 CHARMM36m water simulations. **A-B)** DSSP analysis of the simulations starting from **A)** an extended conformation and **B)** a helical conformation. The green bar on each plot indicates the time from which equilibrium analysis was performed. **C-F)** Histograms of the projections of the simulation coordinates onto PC1 (**C** and **E**) and PC2 (**D** and **F**) built from the first and second halves of the equilibrated part of the extended (**C** and **D**) and helical (**E** and **F**) trajectories. The y axes maxima are set to 3 to allow comparison between all of the TP2 and ONEG histograms (**Figures S29-S40 C-F**), however zoomed in inserts are provided when the largest peak is less than 1. **G)** The FES with respect to PC1 and PC2 built from the combined equilibrated parts of the two trajectories. The energy minimum is set to 0 and the colourbar range is fixed at 0-18 kJ mol^{-1} to allow comparison between all of the TP2 and ONEG FESs (**Figures S29-S40 G**). The DASH clusters are overlayed on the surface as red crosses, and those that occupy a similar PC space are grouped into macrostates with the corresponding structural representatives shown as a superposition of the cluster centroids. The structures are shown as ribbons with random coil, turn and bend

residues shown in red, α-helical residues shown in dark blue, 3_{10}-helical residues shown in light blue, β-bridge residues shown in cyan and β-ladder residues shown in lime. The percentage of trajectory frames occupied by each macrostate is also shown.

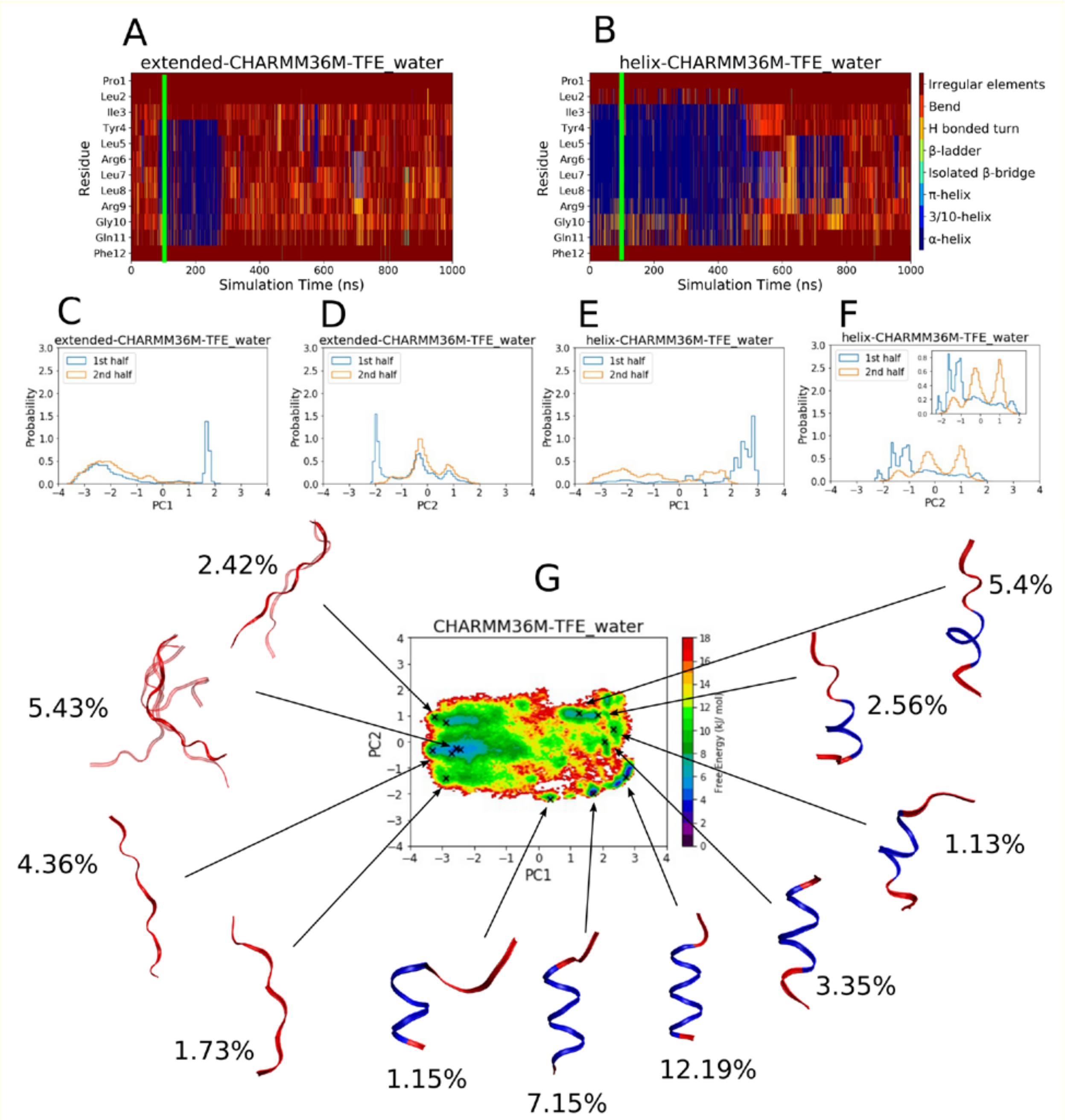

Figure S33) Results from the TP2 CHARMM36m TFE:water simulations. **A-B)** DSSP analysis of the simulations starting from **A)** an extended conformation and **B)** a helical conformation. The green bar on each plot indicates the time from which equilibrium analysis was performed. **C-F)** Histograms of the projections of the simulation coordinates onto PC1 (**C** and **E**) and PC2 (**D** and **F**) built from the first and second halves of the equilibrated part of the extended (**C** and **D**) and helical (**E** and **F**) trajectories. The y axes maxima are set to 3 to allow comparison between all of the TP2 and

ONEG histograms (**Figures S29-S40 C-F**), however zoomed in inserts are provided when the largest peak is less than 1. **G)** The FES with respect to PC1 and PC2 built from the combined equilibrated parts of the two trajectories. The energy minimum is set to 0 and the colourbar range is fixed at 0-18 kJ mol^{-1} to allow comparison between all of the TP2 and ONEG FESs (**Figures S29-S40 G**). The DASH clusters are overlayed on the surface as black crosses, and those that occupy a similar PC space are grouped into macrostates with the corresponding structural representatives shown as a superposition of the cluster centroids. The structures are shown as ribbons with random coil, turn and bend residues shown in red, α-helical residues shown in dark blue, 3_{10}-helical residues shown in light blue, β-bridge residues shown in cyan and β-ladder residues shown in lime. The percentage of trajectory frames occupied by each macrostate is also shown.

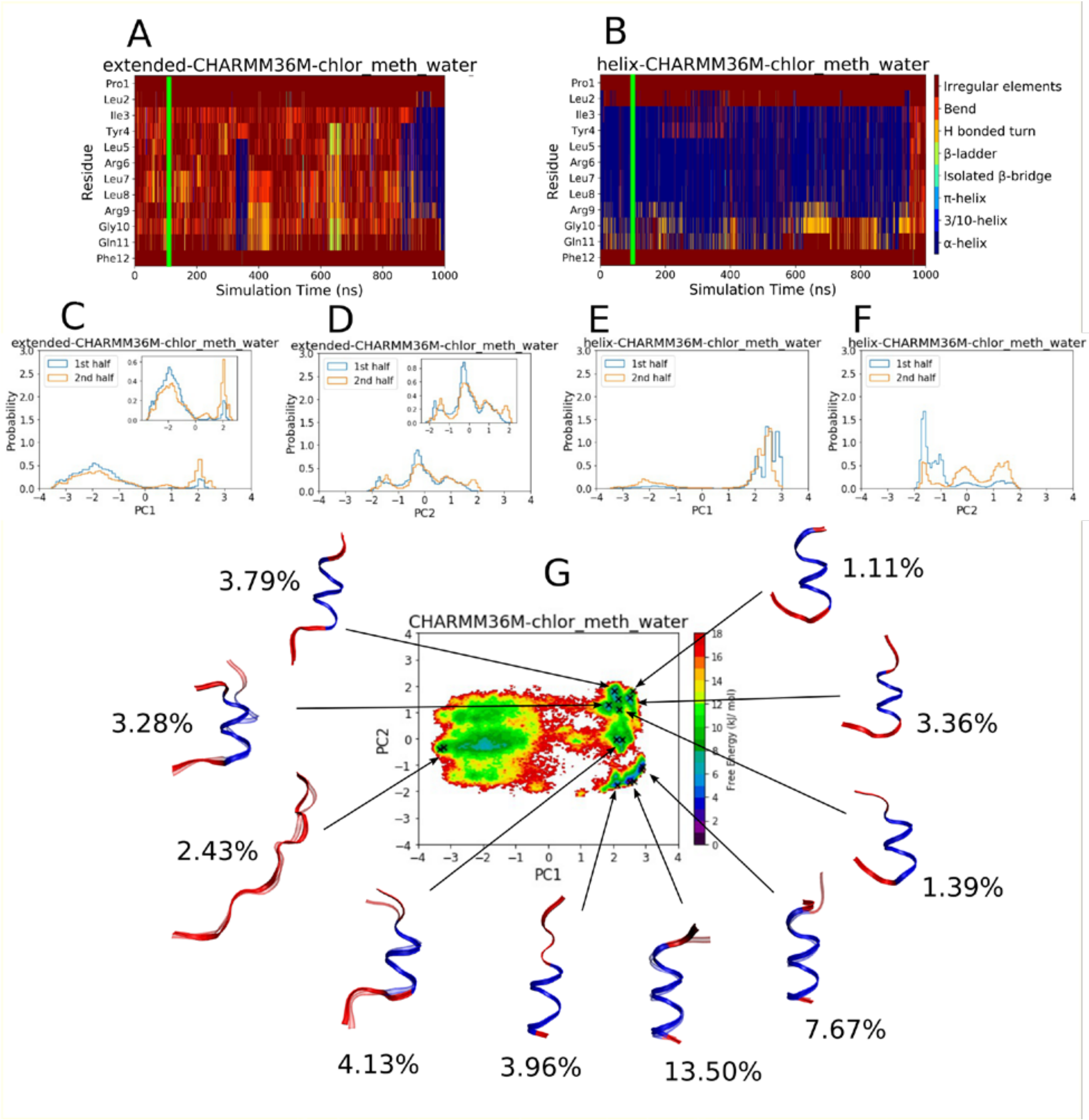

Figure S34) Results from the TP2 CHARMM36m chloroform:methanol:water simulations. **A-B)** DSSP analysis of the simulations starting from **A)** an extended conformation and **B)** a helical conformation. The green bar on each plot indicates the time from which equilibrium analysis was performed. **C-F)** Histograms of the projections of the simulation coordinates onto PC1 (**C** and **E**) and PC2 (**D** and **F**) built from the first and second halves of the equilibrated part of the extended (**C** and **D**) and helical (**E** and **F**) trajectories. The y axes maxima are set to 3 to allow comparison between all of the TP2 and ONEG histograms (**Figures S29-S40 C-F**), however zoomed in inserts are provided when the largest peak is less than 1. **G)** The FES with respect to PC1 and PC2 built from the combined equilibrated parts of the two trajectories. The energy minimum is set to 0 and the colourbar range is fixed at 0-18 kJ mol^{-1} to allow comparison between all of the TP2 and ONEG FESs (**Figures S29-S40 G**). The DASH clusters are overlayed on the surface as black crosses, and those that occupy a similar PC space are grouped into macrostates with the corresponding structural representatives shown as a superposition of the cluster centroids. The structures are shown as ribbons with random coil, turn and bend residues shown in red, α-helical residues shown in dark blue, 3_{10}-helical residues shown in light blue, β-bridge residues shown in cyan and β-ladder residues shown in lime. The percentage of trajectory frames occupied by each macrostate is also shown.

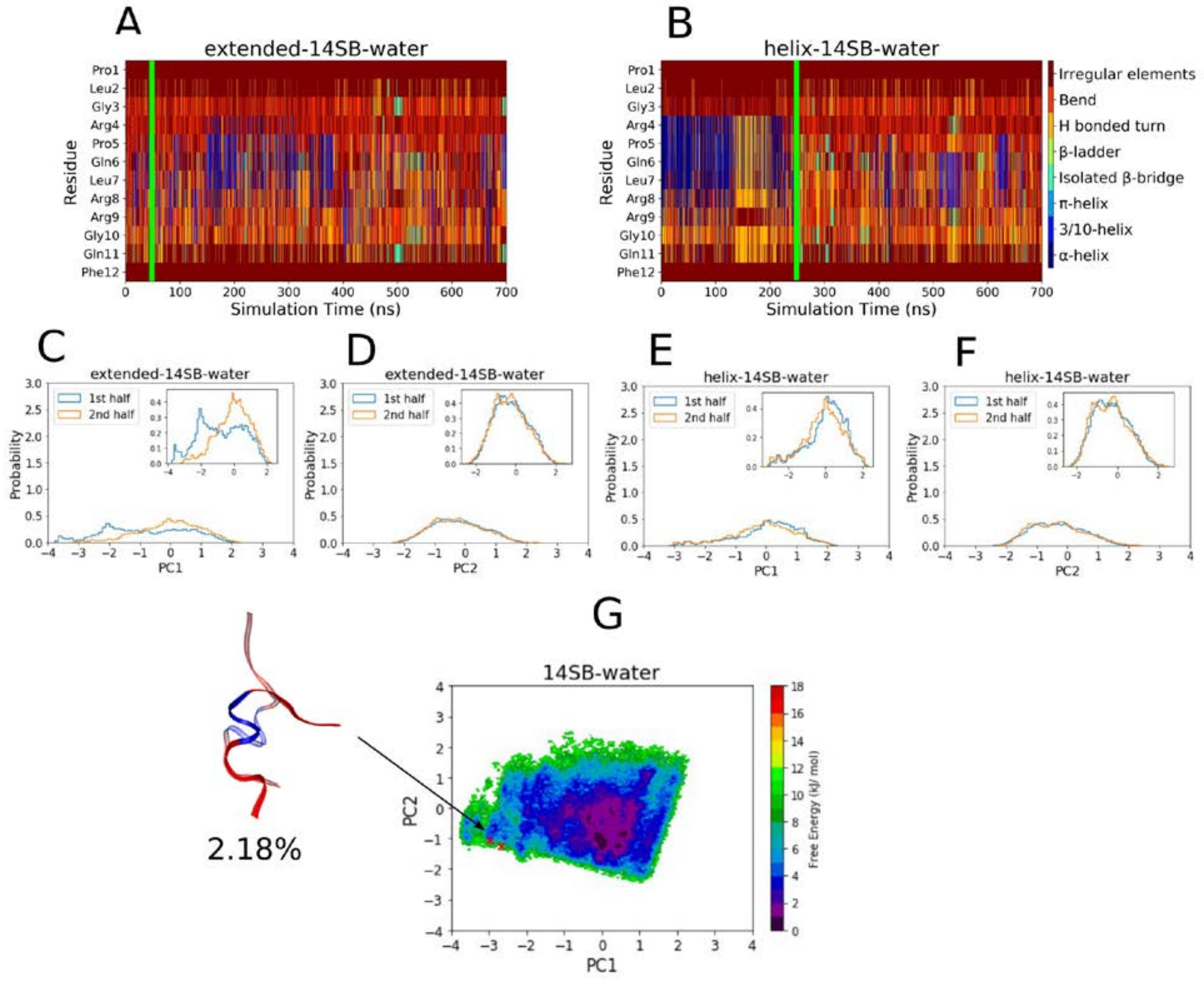

Figure S35) Results from the ONEG ff14SB water simulations. **A-B)** DSSP analysis of the simulations starting from **A)** an extended conformation and **B)** a helical conformation. The green bar on each plot indicates the time from which equilibrium analysis was performed. **C-F)** Histograms of the projections of the simulation coordinates onto PC1 (**C** and **E**) and PC2 (**D** and **F**) built from the first and second halves of the equilibrated part of the extended (**C** and **D**) and helical (**E** and **F**) trajectories. The y axes maxima are set to 3 to allow comparison between all of the TP2 and ONEG histograms (**Figures S29-S40 C-F**), however zoomed in inserts are provided when the largest peak is less than 1. **G)** The FES with respect to PC1 and PC2 built from the combined equilibrated parts of the two trajectories. The energy minimum is set to 0 and the colourbar range is fixed at 0-18 kJ mol^{-1} to allow comparison between all of the TP2 and ONEG FESs (**Figures S29-S40 G**). The DASH clusters are overlayed on the surface as red crosses, and those that occupy a similar PC space are grouped into macrostates with the corresponding structural representatives shown as a superposition of the cluster centroids. The structures are shown as ribbons with random coil, turn and bend residues shown in red, α-helical residues shown in dark blue, 3_{10}-helical residues shown in light blue, β-bridge residues shown in cyan and β-ladder residues shown in lime. The percentage of trajectory frames occupied by each macrostate is also shown.

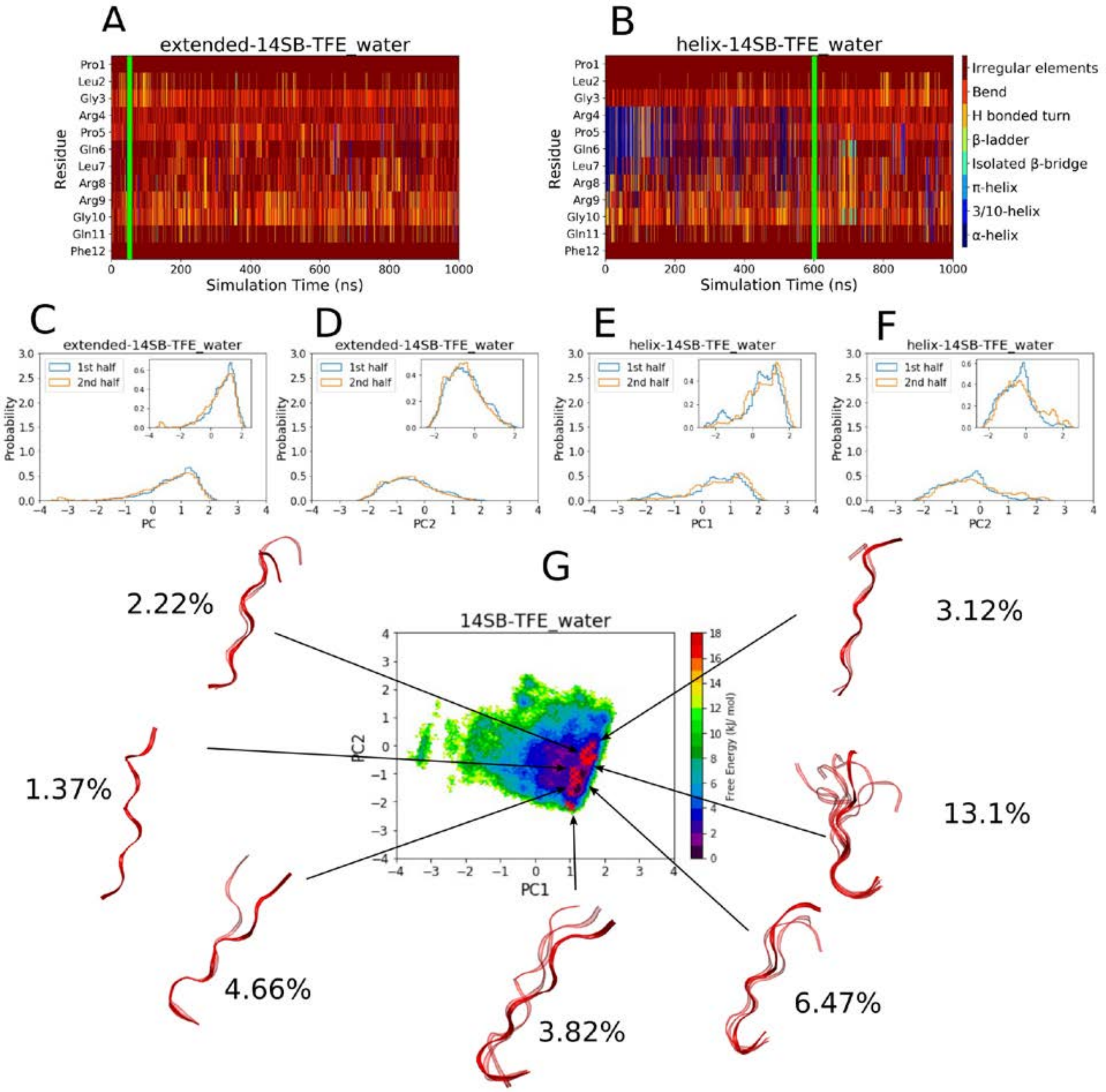

Figure S36) Results from the ONEG ff14SB TFE:water simulations. **A-B)** DSSP analysis of the simulations starting from **A)** an extended conformation and **B)** a helical conformation. The green bar on each plot indicates the time from which equilibrium analysis was performed. **C-F)** Histograms of the projections of the simulation coordinates onto PC1 (**C** and **E**) and PC2 (**D** and **F**) built from the first and second halves of the equilibrated part of the extended (**C** and **D**) and helical (**E** and **F**) trajectories. The y axes maxima are set to 3 to allow comparison between all of the TP2 and ONEG histograms (**Figures S29-S40 C-F**), however zoomed in inserts are provided when the largest peak is less than 1. **G)** The FES with respect to PC1 and PC2 built from the combined equilibrated parts of the two trajectories. The energy minimum is set to 0 and the colourbar range is fixed at 0-18 kJ mol^{-1} to allow comparison between all of the TP2 and ONEG FESs (**Figures S29-S40 G**). The DASH clusters are overlayed on the surface as red crosses, and those that occupy a similar PC space are grouped into macrostates with the corresponding structural representatives shown as a superposition of the cluster centroids. The structures are shown as ribbons with random coil, turn and bend residues shown in red, α-helical residues shown in dark blue, 3_{10}-helical residues shown in light blue, β-bridge residues

shown in cyan and β-ladder residues shown in lime. The percentage of trajectory frames occupied by each macrostate is also shown.

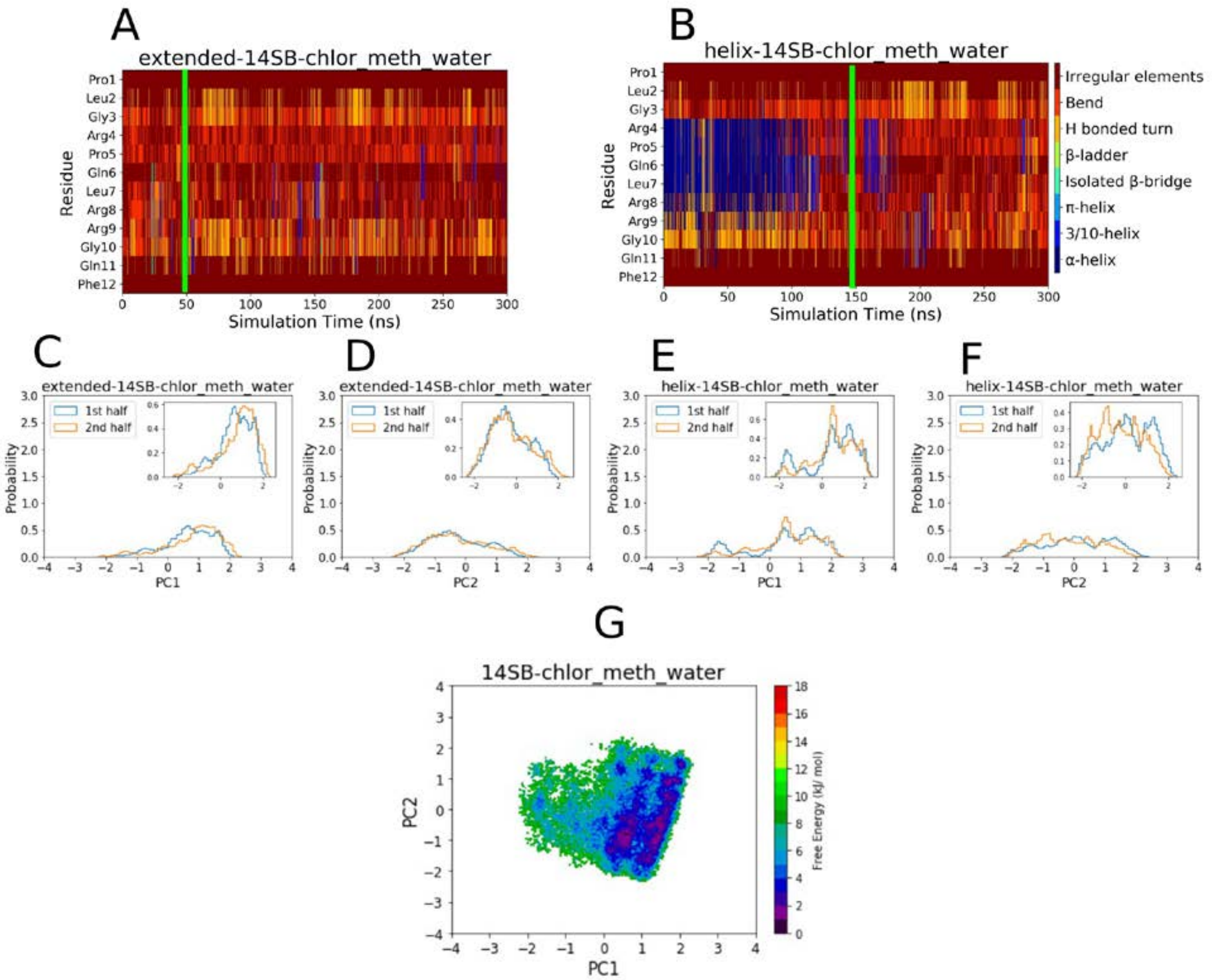

Figure S37) Results from the ONEG ff14SB chloroform:methanol:water simulations. **A-B)** DSSP analysis of the simulations starting from **A)** an extended conformation and **B)** a helical conformation. The green bar on each plot indicates the time from which equilibrium analysis was performed. **C-F)** Histograms of the projections of the simulation coordinates onto PC1 (**C** and **E**) and PC2 (**D** and **F**) built from the first and second halves of the equilibrated part of the extended (**C** and **D**) and helical (**E** and **F**) trajectories. The y axes maxima are set to 3 to allow comparison between all of the TP2 and ONEG histograms (**Figures S29-S40 C-F**), however zoomed in inserts are provided when the largest peak is less than 1. **G)** The FES with respect to PC1 and PC2 built from the combined equilibrated parts of the two trajectories. The energy minimum is set to 0 and the colourbar range is fixed at 0-18 kJ mol^{-1} to allow comparison between all of the TP2 and ONEG FESs (**Figures S29-S40 G**). No DASH clusters were identified.

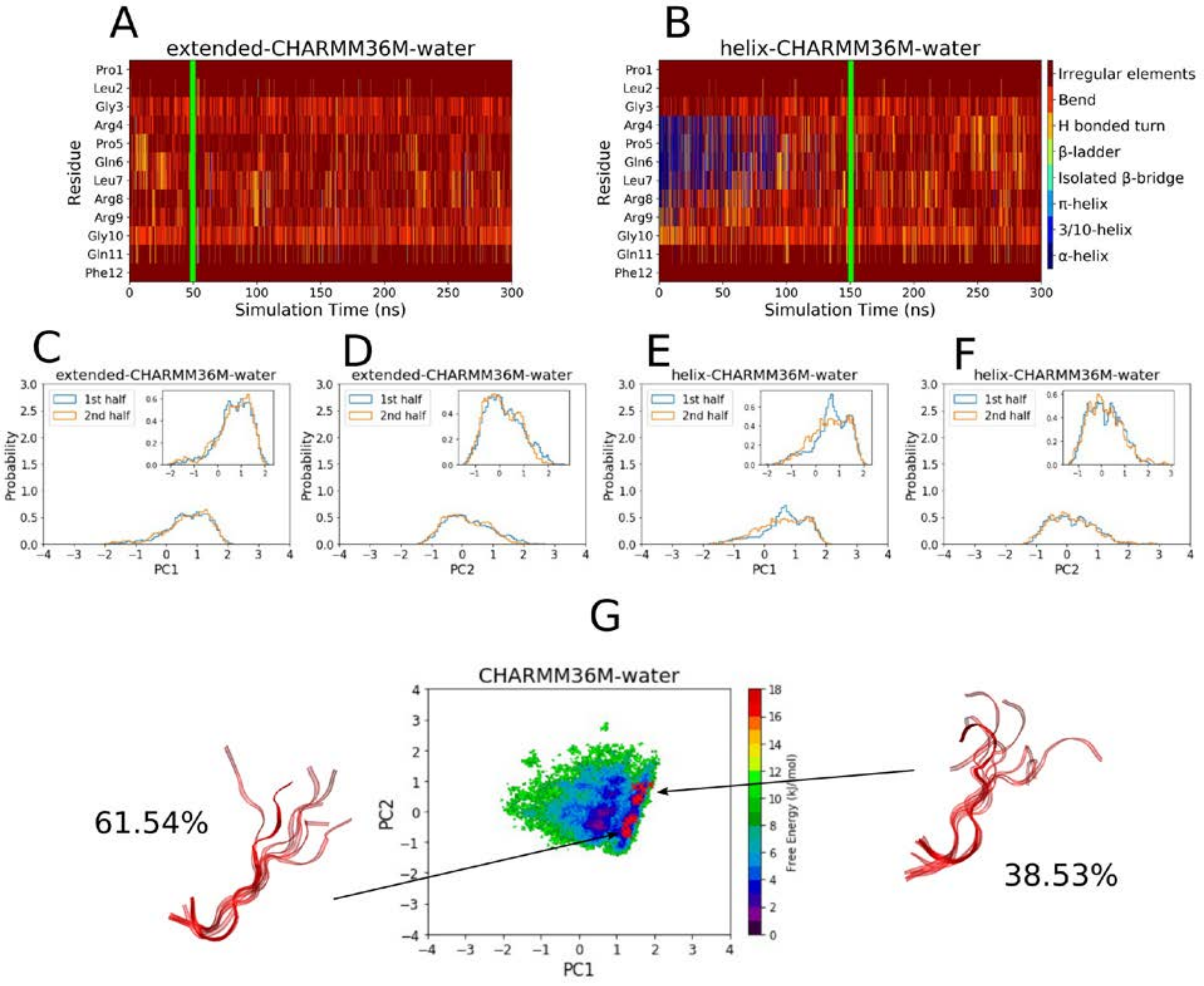

Figure S38) Results from the ONEG CHARMM36m water simulations. **A-B)** DSSP analysis of the simulations starting from **A)** an extended conformation and **B)** a helical conformation. The green bar on each plot indicates the time from which equilibrium analysis was performed. **C-F)** Histograms of the projections of the simulation coordinates onto PC1 (**C** and **E**) and PC2 (**D** and **F**) built from the first and second halves of the equilibrated part of the extended (**C** and **D**) and helical (**E** and **F**) trajectories. The y axes maxima are set to 3 to allow comparison between all of the TP2 and ONEG histograms (**Figures S29-S40 C-F**), however zoomed in inserts are provided when the largest peak is less than 1. **G)** The FES with respect to PC1 and PC2 built from the combined equilibrated parts of the two trajectories. The energy minimum is set to 0 and the colourbar range is fixed at 0-18 kJ mol^{-1} to allow comparison between all of the TP2 and ONEG FESs (**Figures S29-S40 G**). The DASH clusters are overlayed on the surface as red crosses, and those that occupy a similar PC space are grouped into macrostates with the corresponding structural representatives shown as a superposition of the cluster centroids. The structures are shown as ribbons with random coil, turn and bend residues shown in red, α-helical residues shown in dark blue, 3_{10}-helical residues shown in light blue, β-bridge residues shown in cyan and β-ladder residues shown in lime. The percentage of trajectory frames occupied by each macrostate is also shown.

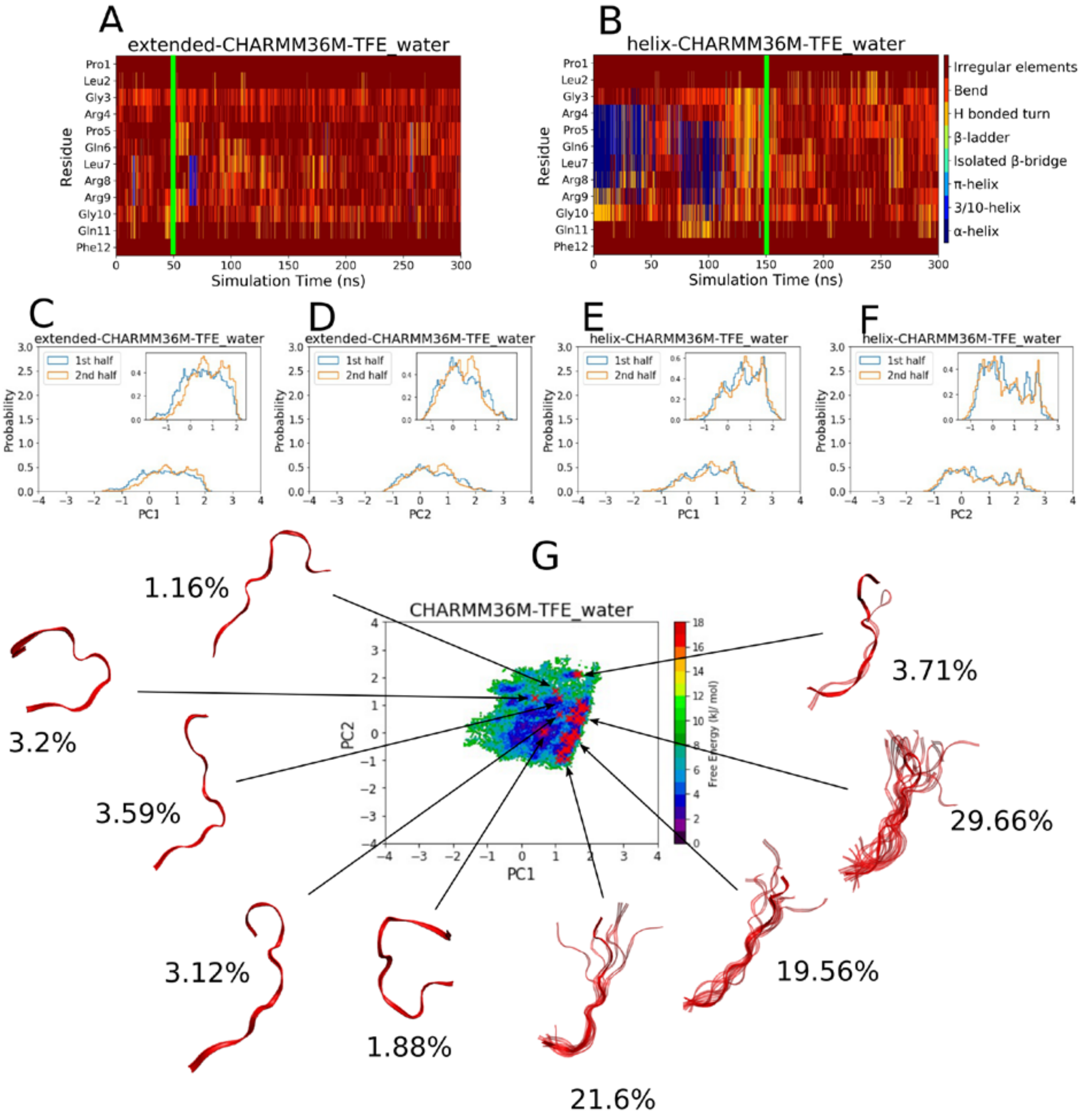

Figure S39) Results from the ONEG CHARMM36m TFE:water simulations. **A-B)** DSSP analysis of the simulations starting from **A)** an extended conformation and **B)** a helical conformation. The green bar on each plot indicates the time from which equilibrium analysis was performed. **C-F)** Histograms of the projections of the simulation coordinates onto PC1 (**C** and **E**) and PC2 (**D** and **F**) built from the first and second halves of the equilibrated part of the extended (**C** and **D**) and helical (**E** and **F**) trajectories. The y axes maxima are set to 3 to allow comparison between all of the TP2 and ONEG histograms (**Figures S29-S40 C-F**), however zoomed in inserts are provided when the largest peak is less than 1. **G)** The FES with respect to PC1 and PC2 built from the combined equilibrated parts of the two trajectories. The energy minimum is set to 0 and the colourbar range is fixed at 0-18 kJ mol^{-1} to allow comparison between all of the TP2 and ONEG FESs (**Figures S29-S40 G**). The DASH clusters are overlayed on the surface as red crosses, and those that occupy a similar PC space are grouped into macrostates with the corresponding structural representatives shown as a superposition of the cluster centroids. The structures are shown as ribbons with random coil, turn and bend

residues shown in red, α-helical residues shown in dark blue, 3_{10}-helical residues shown in light blue, β-bridge residues shown in cyan and β-ladder residues shown in lime. The percentage of trajectory frames occupied by each macrostate is also shown.

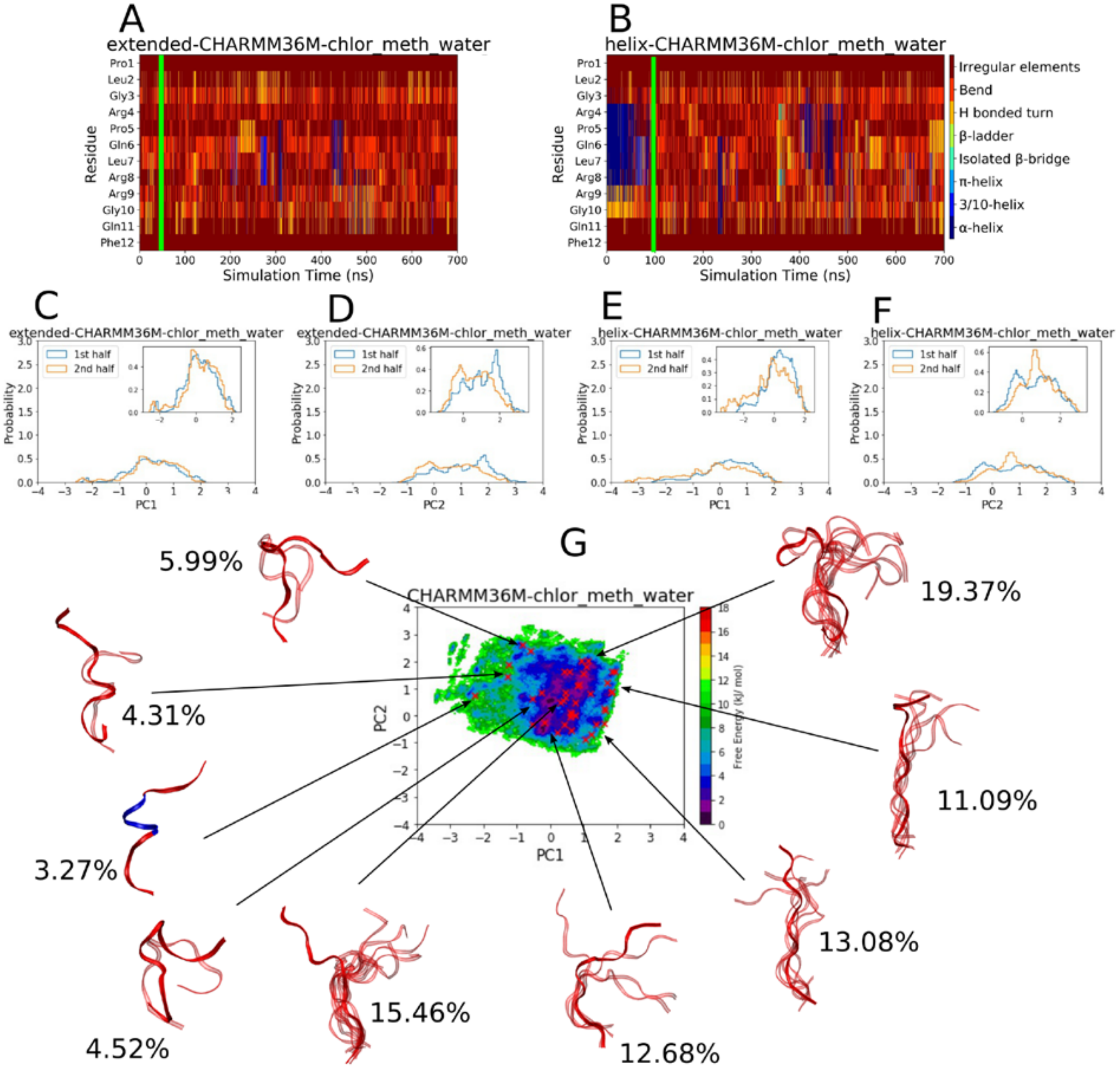

Figure S40) Results from the ONEG CHARMM36m chloroform:methanol:water simulations. **A-B)** DSSP analysis of the simulations starting from **A)** an extended conformation and **B)** a helical conformation. The green bar on each plot indicates the time from which equilibrium analysis was performed. **C-F)** Histograms of the projections of the simulation coordinates onto PC1 (**C** and **E**) and PC2 (**D** and **F**) built from the first and second halves of the equilibrated part of the extended (**C** and **D**) and helical (**E** and **F**) trajectories. The y axes maxima are set to 3 to allow comparison between all of the TP2 and ONEG histograms (**Figures S29-S40 C-F**), however zoomed in inserts are provided when the largest peak is less than 1. **G)** The FES with respect to PC1 and PC2 built from the combined equilibrated parts of the two trajectories. The energy minimum is set to 0 and the colourbar range is fixed at 0-18 kJ mol^{-1} to allow comparison between all of the TP2 and ONEG FESs (**Figures S29-S40 G**). The DASH clusters are overlayed on the surface as

red crosses, and those that occupy a similar PC space are grouped into macrostates with the corresponding structural representatives shown as a superposition of the cluster centroids. The structures are shown as ribbons with random coil, turn and bend residues shown in red, α-helical residues shown in dark blue, 3_{10}-helical residues shown in light blue, β-bridge residues shown in cyan and β-ladder residues shown in lime. The percentage of trajectory frames occupied by each macrostate is also shown.

Replica Trapping Effect

In replica exchange simulations, it is important that the potential energy probability distributions of adjacent replicas overlap sufficiently in order to pass the Metropolis acceptance criterion. In the REST2 protocol the Metropolis acceptance criterion is dependent on the delta term:

$$\Delta_{mn} = (\beta_m - \beta_n)\left[(E_{pp}(X_n) - E_{pp}(X_m)) + \frac{\sqrt{\beta_0}}{\sqrt{\beta_m} + \sqrt{\beta_n}}(E_{ps}(X_n) - E_{ps}(X_m))\right]$$

where β_m and β_n are $1/k_BT_m$ and $1/k_BT_n$, with T_m and T_n being the effective temperatures of replicas m and n, respectively, and k_B being the Boltzmann constant; β_0 is $1/k_BT_0$, with T_0 being the effective temperature of replica 0; E_{pp} and E_{ps} are the energy terms for the protein-protein and protein-solvent interactions, respectively; and X_m and X_n are the coordinates of replicas m and n, respectively (the REST2 protocol is described in more detail in the supporting information and in reference [2]). This means that the potential energy distributions that are being assessed include the intramolecular peptide energy (E_{pp}) plus a scaled peptide-solvent energy ($0.5(\beta_0/\beta_m)^{0.5}E_{ps}$) [2]. In order to select an appropriate replica spacing, short preliminary simulations were performed trialling different numbers of replicas exponentially spaced between effective temperatures 300-600 K. This led to the decision to use ten exponentially spaced replicas for the PLP peptide (**Table S2**) and eight exponentially spaced replicas for TP2 and ONEG (**Table S3**).

However, for some of the PLP peptide simulations we observed conformational-dependent replica trapping, where the base replica was unable to access all of the replica space once the peptide had found the folded conformation. This effect was more significant for the PLP peptide in the membrane-mimicking solvents using the CHARMM force fields, compared to any of the Amber force field simulations or the CHARMM water simulations. The effect was also less significant for the TP2 and ONEG simulations.

This issue can be seen in **Figure S41** that shows replica exchange analysis for the PLP peptide CHARMM36m simulation in chloroform:methanol:water (~ 4.1:5.5:0.4 vol%). **Figure S41 A** shows the travel of replica 0 across the replica space throughout the simulation. For the first 300 ns, replica 0 is able to visit all of the replica Hamiltonians but after 300 ns the system becomes trapped and is unable to access the higher order replicas (except between around 430-500 ns when the replica flips into the higher order replica space and is unable to access the lower order replicas). It is obvious from the replica 0 DSSP assignment plot in **Figure S41 B** that the trapping effect arises when the peptide folds into a helix. Inspection of the first four replica DSSP plots reveals that there is a big structural difference between replicas 1 and 2 between 300-700 ns (**Figure S41 B**). This results in the potential energy distributions shown in **Figure S41 C**, which overlap well for the unfolded structures before 300 ns (top plot) but do not overlap when the peptide folds between 300-700 ns (middle plot). After 700 ns however, replica 2 can be seen to fold into the helical structure and its potential energy distribution shifts closer to replicas 0 and 1 (**Figure S41 C** bottom plot). Nevertheless, the distributions of potential energies after 700 ns do not overlap enough to allow replica 0 to travel freely between the replicas, as the first three replica distributions are shifted, and the replica trapping remains.

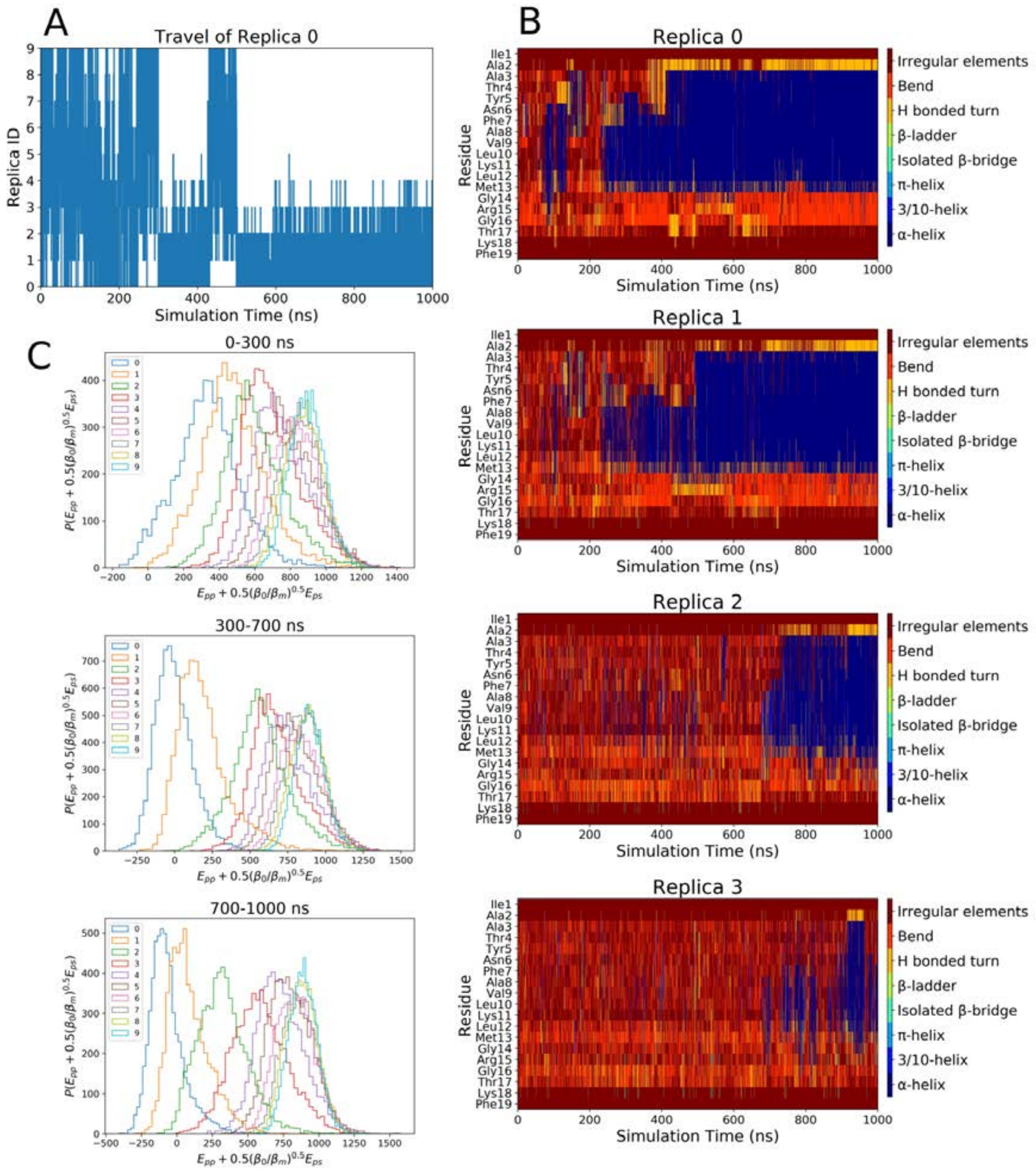

Figure S41) Analysis of the PLP peptide CHARMM36m REST2 simulation starting from an extended conformation in chloroform:methanol:water. A) The travel of replica 0 across the replica space throughout the simulation. B) DSSP assignment plots for replicas 0-3. C) The distribution of the sum of the peptide intramolecular potential energy (Epp) and the scaled potential energy between the peptide and solvent ($0.5(\beta_0/\ \beta_m)^{0.5}E_{ps}$ for each replica, for the first 300 ns (top), from 300-700 ns (middle) and from 700-1000 ns (bottom).

Although it is unclear why this behaviour occurs more significantly with the CHARMM force fields and in the membrane-mimicking solvents, our analyses suggest that the trapping occurs because

at any given time, the CHARMM PLP peptide is folded below a given effective temperature and unfolded above it. This results in a big difference in intramolecular peptide potential energy due to the difference in backbone hydrogen bonding. For the Amber force field simulations, the change from folded to unfolded structures as the replica order increases is more incremental and the potential energy distributions are more evenly spaced (see **Figure S42** for an example).

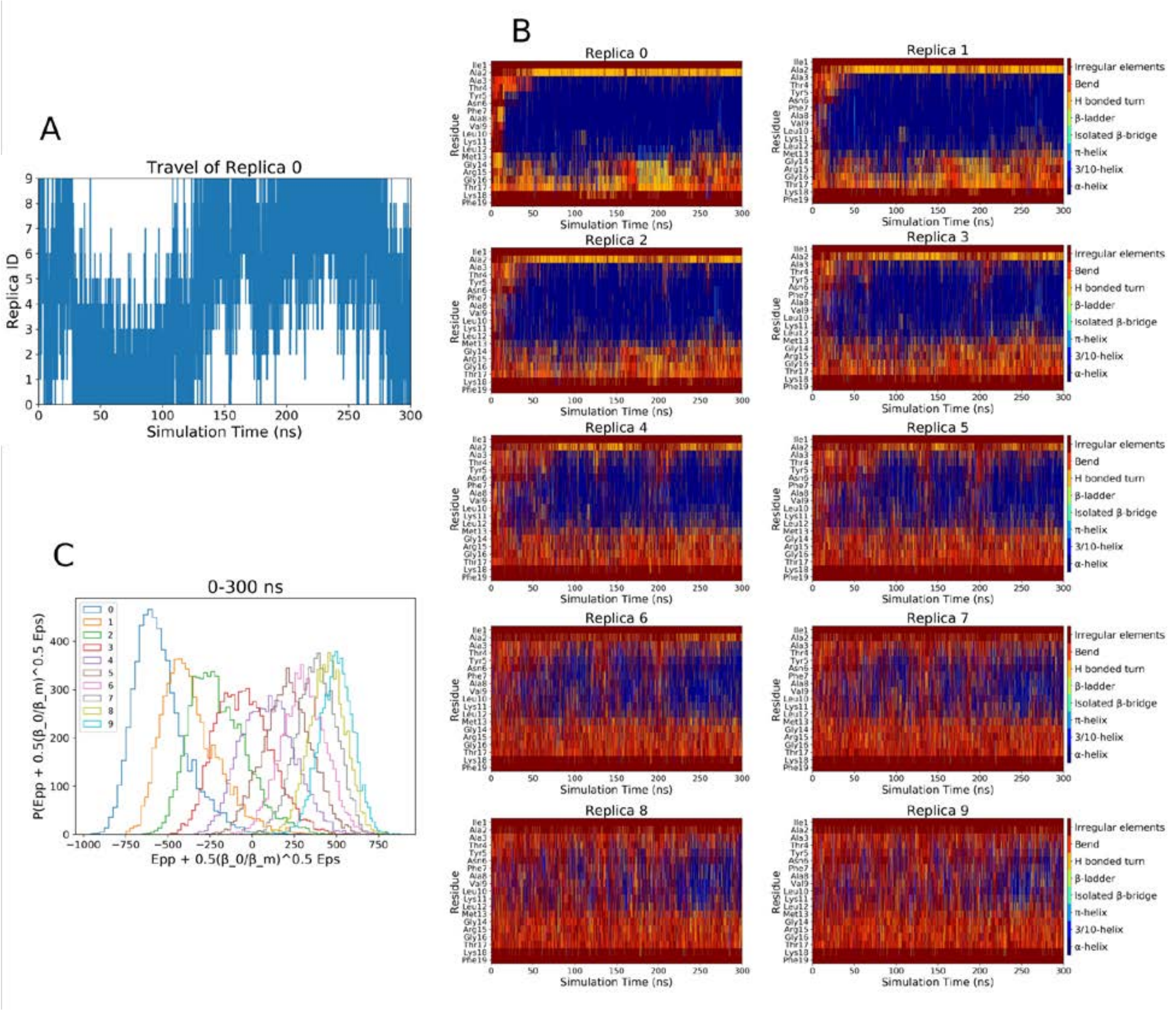

Figure S42) Analysis of the PLP peptide ff14SB REST2 simulation starting from an extended conformation in chloroform:methanol:water. A) The travel of replica 0 across the replica space throughout the simulation. B) DSSP assignment plots for replicas 0-9. C) The distribution of the peptide intramolecular potential energy (Epp) plus the scaled potential energy between the peptide and solvent (0.5 (β_0/β_m)^0.5 Eps) for each replica.

Although the trapping behaviour could mean that the peptide is unable to escape a local energy minimum, we are cautiously optimistic that the sampling procedure is still useful because the

simulation was able to visit many unfolded states before 300 ns and then relaxed into the helical state without bias.

Radius of Gyration and RMSD analysis

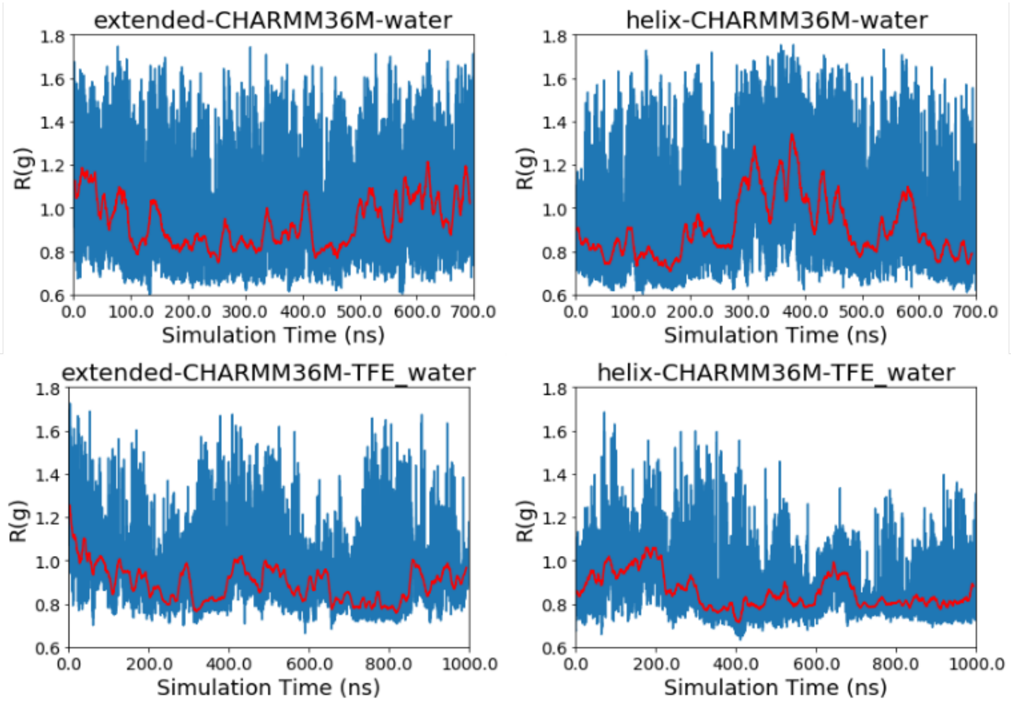

Figure S43) Radius of Gyration (R(g)) of the PLP peptide backbone throughout the CHARMM36M simulations of the peptide in water and TFE-water, starting from extended and helical conformations. A running average is shown in red.

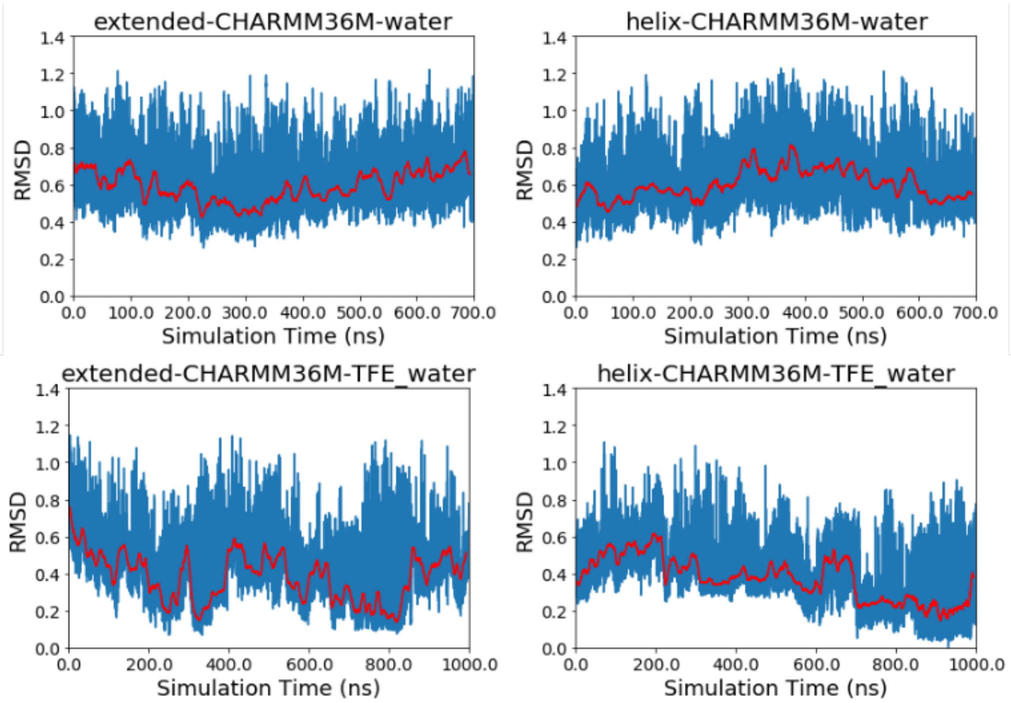

Figure S44) Root mean square deviation (RMSD) of atom positions of the PLP peptide backbone in comparison to a helical structure (the same structure used to seed the helical simulation) throughout the CHARMM36M simulations of the peptide in water and TFE-water, starting from extended and helical conformations. A running average is shown in red.

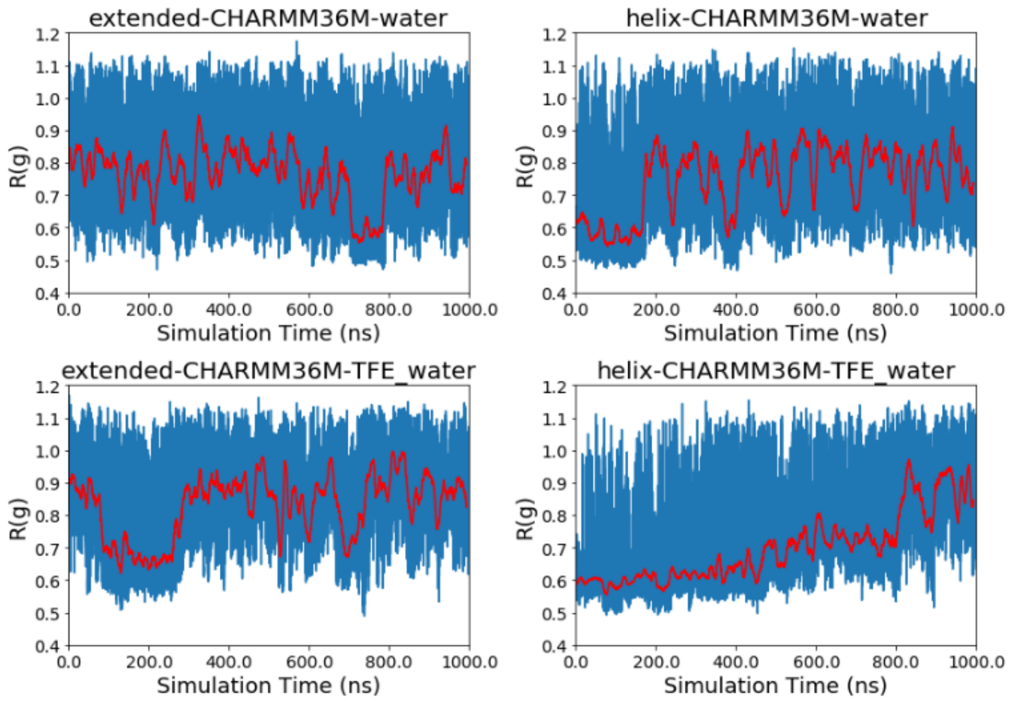

Figure S45) Radius of gyration (R(g)) of the TP2 backbone throughout the CHARMM36M simulations of the peptide in water and TFE-water, starting from extended and helical conformations. A running average is shown in red.

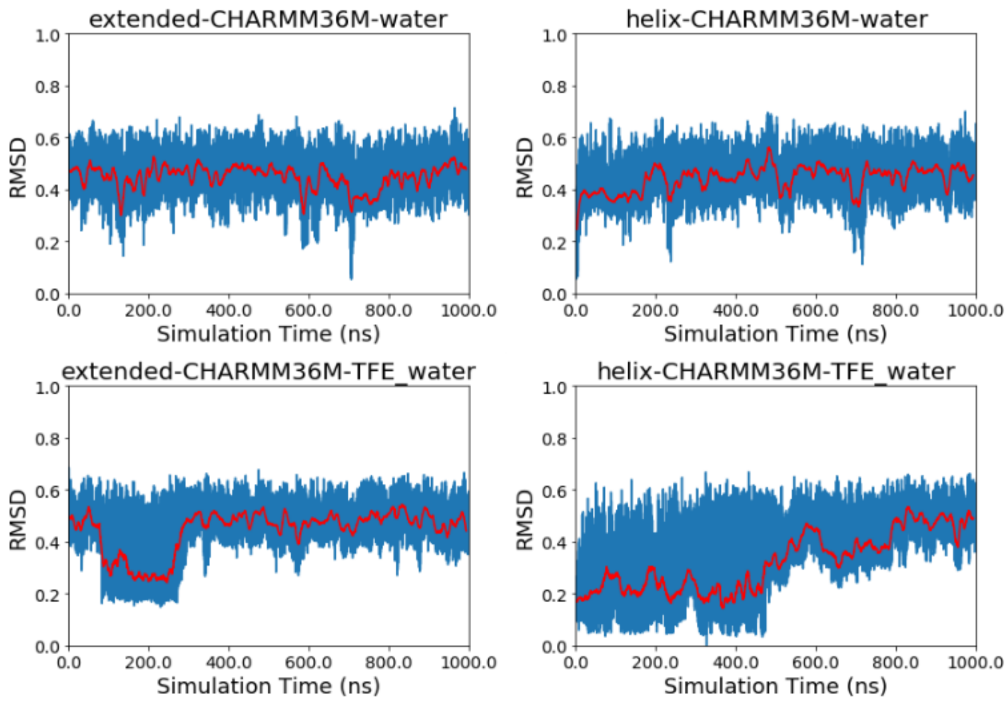

Figure S46) Root mean square deviation (RMSD) of atom positions of the TP2 backbone in comparison to a helical structure (the same structure used to seed the helical simulation) throughout the CHARMM36M simulations of the peptide in water and TFE-water, starting from extended and helical conformations. A running average is shown in red.

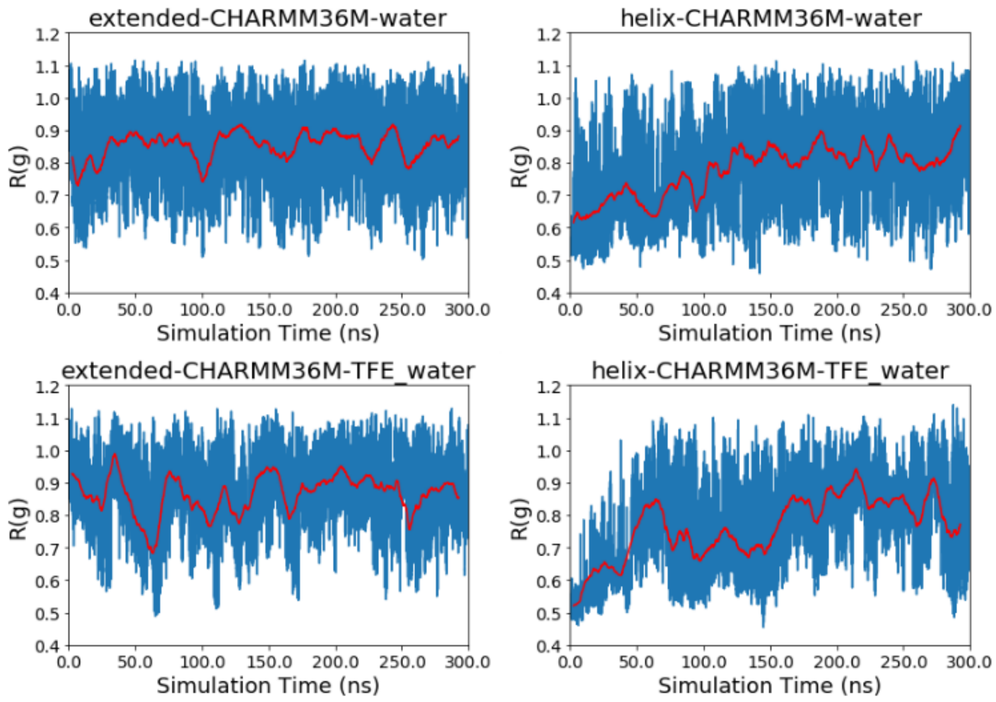

Figure S47) Radius of gyration (R(g)) of the ONEG backbone throughout the CHARMM36M simulations of the peptide in water and TFE-water, starting from extended and partially helical conformations. A running average is shown in red.

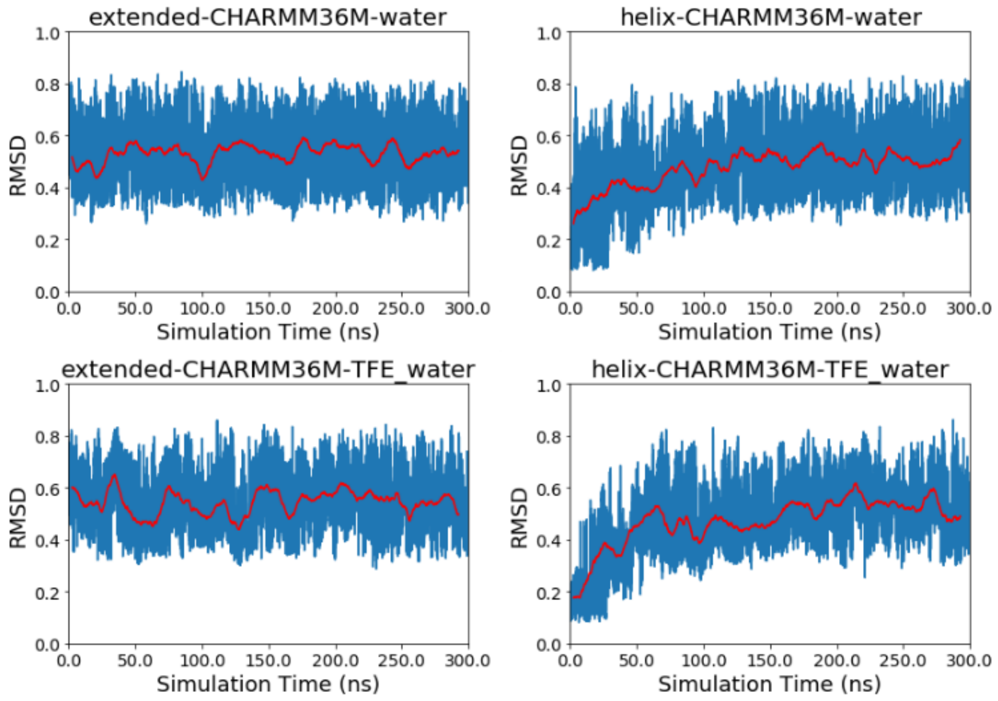

Figure S48) Root mean square deviation (RMSD) of atom positions of the ONEG backbone in comparison to a helical structure (the same structure used to seed the helical simulation) throughout the CHARMM36M simulations of the peptide in water and TFE-water, starting from extended and partially helical conformations. A running average is shown in red.

Comparing the R(g) and RMSD analyses of the PLP peptide, TP2 and ONEG with their respective DSSP analyses reveals that, generally, the R(g) and RMSD tends to fluctuate more in disordered systems (e.g the PLP peptide in water, TP2 in water and ONEG in both solvents). It can also be noted that the R(g) and RMSD from a helical structure decrease when helical structures are formed.

1 D. S. Wishart, C. G. Bigam, J. Yao, F. Abildgaard, H. J. Dyson, E. Oldfield, J. L. Markley and B. D. Sykes, *J. Biomol. NMR*, 1995, **6**, 135–140.

2 L. Wang, R. Friesner and B. J. Berne, *J. Phys. Chem. B*, 2011, **115**, 9431–9438.
